# Deciphering hierarchical regulatory network of cell fate via an epigenetics-informed heterogeneous graph transformer on single-cell multi-omics data

**DOI:** 10.1093/bib/bbaf664

**Published:** 2025-12-12

**Authors:** Yuhong Huang, Chao Liu, Zhiling Yang, Bo Liu, Xiao Zhai, Jiajin Zheng, Jing Xiao, Tao Song

**Affiliations:** Department of Oral Pathology, School of Stomatology, Dalian Medical University, No. 9, Western Section of Lushun South Road, Dalian, Liaoning 116044, China; Department of Oral Pathology, School of Stomatology, Dalian Medical University, No. 9, Western Section of Lushun South Road, Dalian, Liaoning 116044, China; Department of Biomedical Engineering, College of Basic Medical Sciences, Dalian Medical University, No. 9, Western Section of Lushun South Road, Dalian, Liaoning 116044, China; Institute for Genome Engineered Animal Models of Human Diseases, Dalian Medical University, No. 9, Western Section of Lushun South Road, Dalian, Liaoning 116044, China; Department of System Technology, Library, Dalian Medical University, No. 9, Western Section of Lushun South Road, Dalian, Liaoning 116044, China; Department of Biomedical Engineering, College of Basic Medical Sciences, Dalian Medical University, No. 9, Western Section of Lushun South Road, Dalian, Liaoning 116044, China; Department of Oral Pathology, School of Stomatology, Dalian Medical University, No. 9, Western Section of Lushun South Road, Dalian, Liaoning 116044, China; Department of Biomedical Engineering, College of Basic Medical Sciences, Dalian Medical University, No. 9, Western Section of Lushun South Road, Dalian, Liaoning 116044, China

**Keywords:** single-cell multi-omics, heterogeneous graph transformer, hierarchical regulatory network, driver regulators, *in silico* perturbations

## Abstract

The precise control of cell fate is driven by a hierarchical regulatory network (HRNet) where transcription factors (TFs) and cis-regulatory elements (CREs) orchestrate the expression of target genes (TGs) through complex causal actions. While single-cell multi-omics technologies provide multi-dimensional data to resolve regulatory networks, existing methods often fail to capture their hierarchical and causal properties. We propose SMOGT (Single-cell Multi-Omics Graph Transformer), a graph representation learning method to decipher HRNet. SMOGT embeds epigenetic mechanism into Heterogeneous Graph Transformer (HGT) by structuring information flow along a hierarchical-guided meta-path (TF-TF → TF-CRE → CRE-CRE → CRE-TG), and employs a semi-supervised strategy to ensure network accuracy. Validated against ChIP-seq and HiC-seq benchmarked datasets, SMOGT showed significantly higher accuracy in predicting transcriptional regulation (TF-CRE) and long-range chromatin conformation (CRE-CRE). The HRNet scaffolds downstream modules that mechanistically link network architecture to cell fate. The multi-layer random walk (MRWR) module identifies driver regulators and their TGs. The BioStreamNet module predicts shifts in cell fate trajectories following *in silico* perturbations within gene-specific HRNet formed by extracting regulatory weights during TG expression prediction. In hematopoietic stem cell differentiation, SMOGT elucidated the hierarchical causal cascade from driver TFs that governs lineage commitment. In melanoma epithelial-to-mesenchymal transition (EMT), it revealed a critical therapeutic window for reversing the process, and in Acute Myeloid Leukemia (AML), it uncovered hub-CREs with significant prognostic value. By accurately modeling hierarchical causality, SMOGT provides a robust tool to dissect and predict cell fate dynamics in both development and disease.

## Introduction

Modeling cell fate specification and determination for constructing virtual cells represents a foundational challenge in biomedicine [1, 2]. Cell fate is driven by a hierarchical regulatory network (HRNet) mediated by core transcription factors (TFs) and CREs [3–5]. This network originates from interactions between TFs and their cofactors (TF/co-TF), subsequently transmitting signals through the binding of TF-CRE modules, and then amplifying and integrating these signals via long-range CRE-CRE interactions to ultimately achieve specific spatiotemporal regulation of target gene (TG) expression. In the fields of single-cell multi-omics, integration algorithms have provided a solid methodological foundation for the multi-dimensional dissection of cell states [6, 7]. Deep generative models, such as totalVI [8] and MultiVI [9], achieve high-precision mapping and denoising of different omics profiles by constructing a shared latent space across modalities. Matrix factorization models, like MOFA+ [10] and MCSL-LTC [11], achieve integration by extracting cross-omics shared factors that capture conserved biological patterns between modalities. Manifold alignment models, such as Seurat [12], and graph embedding models, such as NIC [13], focus on aligning and optimizing inter-cellular relationships to generate smooth integrated atlases. Building upon systematic single-cell multi-omics integration algorithms, a more profound problem arises: how to identify the hierarchical regulatory logic underlying cell state transitions and fate determination. This requires constructing a hierarchical regulatory model that goes beyond identification of core driver regulators to elucidate the logic behind cell fate.

To infer regulatory network from single-cell multi-omics data that are suitable for downstream driver regulators identification and perturbation analysis, early studies relied on statistical correlation (SC) and linear regression (LR) to establish links among TF expression, CRE accessibility and TG expression. Representative methods include correlation coefficient-based FigR [14], TRIPOD [15] and multiple regression-based SCENIC+ [16]. The biological basis for SC and LR models is that regulatory logic can be captured by expression correlation. However, these models are limited in distinguishing direct from indirect effects and fail to capture the nonlinear, long-range interactions between CREs that are critical for epigenetic regulation [4, 17]. To better delineate causality, subsequent approaches evolved in two directions: one employing probabilistic graphical models (PGMs), such as scMTNI and MAGICAL [18, 19], and the other integrating prior knowledge with machine learning strategies. STREAM, for instance, modeled TF-CRE regulatory inference as a Steiner Forest problem [20], and the later REUNION utilized semi-supervised learning with a small set of high-confidence interactions as pseudo-labels to guide genome-wide predictions on TF-bindings [21]. Despite these advances, such methods often face a balance between model complexity and biological completeness. PGMs frequently model CREs implicitly or omit them to reduce complexity, while the efficiency constraints of traditional machine learning restrict STREAM and REUNION to modeling only a subset of TF-CRE-TG relationships.

To simulate driver signals propagate more accurately, recent deep learning models like regX and LINGER have integrated protein–protein interaction (PPI) networks [22, 23]. This strategy demonstrated that an idea model must extend beyond direct TF-TG regulation to include the downstream interactions of gene products within the PPI network [24, 25], thus offering a more complete picture of the signaling cascade. However, the simple architectures of these models lead to insufficient integration of prior knowledge, thereby limiting their accuracy in capturing complex network relationships [26–28]. Graph neural network (GNN) integrating complex prior networks have emerged to overcome this limitation. However, existing models like CEFCON [29] remain confined to single-omic data, thus failing to incorporate epigenetic information. DeepMAPS [30] and scMultiomeGRN [31] were the first to apply the Heterogeneous Graph Transformer (HGT) to single-cell multi-omics data [32], achieving SOTA performance in regulatory network inference and cell classification. Nevertheless, these advanced HGT models have limitations in their application of the attention mechanism, attention in DeepMAPS is constrained to a bipartite graph of genes and cells, while scMultiomeGRN is confined to two omics, chromatin accessibility and gene expression. In both cases, the attention mechanism does not conform to the logic of hierarchical epigenetic regulation.

At the resolution of single-cell multi-omics, despite algorithmic efforts to infer functional regulatory networks, the field returns to a core biological question: which TFs and CREs causally govern cell fate. Tackling it requires not only identifying these elements but also validating their functional impact through perturbation modeling. SCENIC+ introduced the use of perturbation prediction to benchmark network performance. This strategy was subsequently advanced by CellOracle [33] and regX, which integrated with cell fate modeling. However, their groundbreaking work still relies on single-layer network [34, 35], which are confined to TF-CRE or TF-TG interactions. By overlooking TF-TF synergy and the long-range relationships among CREs, the information flow in perturbation simulations diverges from true epigenetic mechanisms, thus reducing the accuracy of perturbation modeling.

In summary, a key limitation of current methods is their reliance on single-layer networks, failing to model the complete hierarchical flow of epigenetic information (TF-TF → TF-CRE → CRE-CRE → CRE-TG), thereby compromising the accuracy of driver regulator identification and perturbation modeling. To address this, we propose Single-cell Multi-Omics Graph Transformer (SMOGT), a HGT framework that constructs HRNet by focusing attention along an epigenetically guided meta-path. Its downstream modules then decode cell fate regulation by identifying driver regulators, modeling perturbation responses and discovering co-CRE modules. We demonstrate its biomedical utility by identifying prognostic CREs in the Acute Myeloid Leukemia (AML) microenvironment, pinpointing lineage-defining TFs in hematopoiesis, and accurately predicting perturbation responses in both melanoma and hematopoietic systems.

## Materials and Methods

### Datasets

We collected paired scRNA-seq and scATAC-seq datasets from GEO, 10x Genomics, and ENCODE (Supplementary Table S1), including: (i) Bone marrow (BM); (ii) healthy peripheral blood mononuclear cells (PBMC); (iii) human cerebral cortex data; (iv) leukemia stem cells (LSCs); (v) K562 and HCT116 cell line data; (vi) A549 and GM12878 cell line data; (vii) K562 perturb-seq data.

Ground-truth regulatory networks for the BM, K562, HCT116, A549, and GM12878 are derived from ChIP-seq (Supplementary Table S2) and Hi-C seq (Supplementary Table S3) (see details on in Supplementary Note S1). We adopted the same text mining approach as eNet2 [36] to obtain GWAS SNPs data for BM, PBMC and CC from public databases: open GWAS (https://gwas.mrcieu.ac.uk/) and GWAS Catalog (https://www.ebi.ac.uk/gwas/home), retaining only processed SNPs significantly associated with diseases. PBMC eQTL data are derived from the Blood eQTL project (https://www.eqtlgen.org/), retaining only loci with FDR < 1 × 10^−150^ and not located within gene bodies. CC eQTLs were obtained from the GTEx database (https://www.gtexportal.org/home). SE data for BM, PBMC, CC, K562, HCT116, A549, and GM12878 were acquired from the SEanalysis database (http://licpathway.net/SEanalysis/home).

Then, we performed data quality control, dimensionality reduction, and clustering following the Seurat pipeline (https://satijalab.org/seurat/). We used SEACells to convert Seurat objects into metacells [37], and annotated BM cells as previously described [38], which was followed by Monocle3 (https://cole-trapnell-lab.github.io/monocle3/) and scVelo (https://scvelo.readthedocs.io/en/stable/) analyses with HSC as the root node (see details on data preprocessing and model input in Supplementary Note S1).

### The single-cell multi-omics graph transformer framework

**Figure 1:**
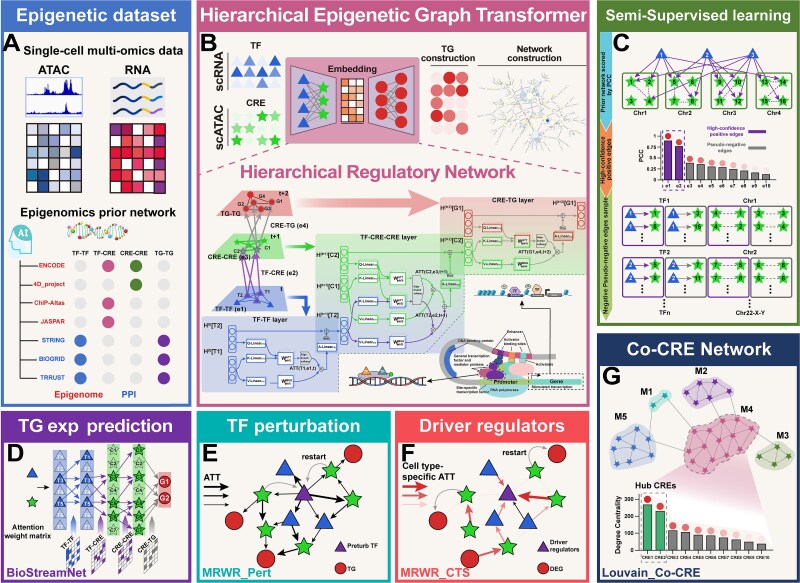
The framework of SMOGT. (A) The data input consists of two components: (i) paired single-cell multi-omics data (scATAC-seq + scRNA-seq), and (ii) curated prior network datasets, totally seven types of resources including epigenomic databases (e.g. ENCODE) and PPIs databases (e.g. STRING, the source of TG-TG interactions in this study). (B) The core architecture of SMOGT is a hierarchical epigenetic HGT, which central principle updates for three node types through iterative representation (TFs, CREs and TGs) by following the hierarchical sequence of TF-TF → TF-CRE → CRE-CRE → CRE-TG interactions, thereby mapping transcription profiles and regulatory network information of multi-omics into a low-dimensional space. (C) SMOGT adopts a semi-supervised learning with adversarial training, which selectively samples the high-confidence positive edges from both TF-CRE and CRE-CRE relationships, while strategically incorporates pseudo-negative edges at controlled ratios. SMOGT leverages the learned node embeddings to construct a HRNet, which yields four downstream modules by combining with three network algorithms. (D) The BioStreamNet module builds a 4-layer biologically constrained neural network based on HRNet’s knowledge flow to predict TG expression from TF and CRE profiles. (E) The MRWR_Pert module constructs node transition matrices using attention weights and performs RWR to predict TF perturbation effects. (F) The MRWR_CLS module incorporates cell-type-specific differential expression values to weight the transition matrices, enabling context-aware identification of driver regulators. (G) The Co-CRE network module applies the Louvain algorithm to identify co-regulatory CRE modules within the HRNet.

SMOGT was utilized to construct context-specific TF-CRE-TG networks via meta-paths guided by epigenetic mechanism. Specifically, SMOGT adopted the paired scATAC-seq and scRNA-seq data as initial features for TFs, CREs, and TGs (Fig. 1A), and optimized node representations and topology of the HRNet—comprising TF-TF, TF-CRE, CRE-CRE, and CRE-TG edges—via a Hierarchical Epigenetic Graph Transformer (Fig. 1B). A semi-supervised learning strategy balanced the contradiction between the sparsity of known edges and the complexity of HRNet during this optimization (Fig. 1C). The BioStreamNet module leveraged the learned TF-CRE-TG relationships to predict TG expression (Fig. 1D), and the multi-layer random walk with restart (MRWR)_Pert and MRWR_CTS modules to identify TF-perturbed genes and driver regulators, respectively (Fig. 1E and F). The Louvain_Co-CRE module discovered co-CRE subnetworks using learned CRE representations (Fig. 1G).

#### Hierarchical epigenetic graph transformer

Hierarchical Epigenetic Graph Transformer is the core architecture of SMOGT, which follows the central rule (TF-CRE-TG) and employs three types of GNN-based feature encoders: (i) TF-TF Encoder based on GCN (Graph Convolutional Networks); (ii) TF-CRE, CRE-CRE, and CRE-TG encoders based on HGT; (iii) Loop Encoder based on GraphSAGE.

### Graph neural network

Given a sampled heterogeneous sub-graph, feature encoder extracts all linked node pairs and aggregates information from source nodes to get a contextualized representation for the target node. We denote the embedding of ${v}_s$ and ${v}_t$ on the $l$-th encoder layer as ${H}_s^l$ and ${H}_t^l$, $l=1,2,\dots, L$.

The first step is to calculate the mutual attention between source node $s$ and target node $t$. We first give a brief introduction to the general attention-based GNN as follows:


(1) \begin{equation*} {H}_t^l\leftarrow \frac{\mathrm{Aggregate}}{\forall{v}_s\in N\left({v}_t\right),\kern0.5em \forall e\in E\left(s,t\right)}\left(\mathrm{Attention}\left(s,t\right)\cdot \mathrm{Message}(s)\right) \end{equation*}

There are three basic operators: Attention, which estimates the importance of each source node; Message, which extracts the message by using only the source nodes; and Aggregate, which aggregates the neighborhood message by the attention weight.

### GCN


(2) \begin{equation*} {H}_t^l=\sigma \left({\overset{\sim }{D}}^{-\frac{1}{2}}\overset{\sim }{A}{\overset{\sim }{D}}^{-\frac{1}{2}}{H}_s^{l-1}{W}^{l-1}\right) \end{equation*}

where $\overset{\sim }{A}=A+I$, $A$ is the adjacency matrix and $I$ is the identity matrix ensuring that each target node includes its own features during aggregation, ${\overset{\sim }{D}}_{ii}={\sum}_j{\overset{\sim }{A}}_{ij}$ indicates the degree of node $i$, ${W}^{l-1}$ is a layer-specific trainable weight matrix. $\sigma \left(\cdotp \right)$ denotes an activation function (e.g. ReLU, Sigmoid). ${H}_s^{l-1}$ is the activation matrix of ${v}_S$ in the $l-1$th layer, ${H}^0={X}^{\mathrm{TF}}$ (feature matrix of TFs).

GCN was specifically employed to encode the TF-TF interaction layer. This selection was empirically driven, as the GCN architecture yielded superior prediction accuracy and stability when compared to GAT and HGT.

### GraphSAGE


(3) \begin{equation*} {H}_t^l={W}^{l-1}\cdot CONCAT\left({H}_s^{l-1}, AGGREGATE\left(\left\{{H}_s^{l-1},\forall{v}_S\in N\left({v}_t\right)\right\}\right)\right) \end{equation*}

where $AGGREGATE\left(\cdot \right)$ is the neighbor feature aggregation function which can be operations such as averaging, pooling, or LSTM; $CONCAT\left(\cdot \right)$ is the feature concatenation function which preserves the target node’s own information and the global information of its neighbors.

### HGT


(4) \begin{equation*} {H}_t^l=\theta \left( ReLU\left({\overset{\sim }{H}}_t^l\right)\right){\overset{\sim }{H}}_t^l+\left(\theta -1\right){H}_t^{l-1} \end{equation*}

where $\theta$ is a trainable parameter and *ReLU* is the activation function, ${\overset{\sim }{H}}_t^l={}_{\forall{v}_s\in N(t)}{}^{\oplus}\left( Att-{HGT}_{\left(s,e,t\right)}\cdot MSG-{HGT}_{\left(s,e,t\right)}\right)$ is the updated vector obtained by aggregating heterogeneous mutual attention $Att-{HGT}_{\left(s,e,t\right)}$ weighted information $MSG-{HGT}_{\left(s,e,t\right)}$ to the target node ${v}_t$ from all its neighbors (see details in Supplementary Note S2).

#### Dual-task decoder

The network reconstruction decoder uses a dot product followed by a sigmoid activation function, forming a binary classifier. The gene expression prediction decoder is a 4-layer Fully Connected Neural Network (FCNN) for regression. The layer dimensions are: embedding_dim, hidden_dim, hidden_dim2, hidden_dim3, raw_dim. A ReLU activation function is used between layers. In our study, embedding_dim and hidden_dim were set to 16 and 32, respectively.

#### Loss function and training process

Our goal is to simultaneously learn node feature representations and network topological structure, thus adopting a weighted combined loss function (see details in Supplementary Note S3). The Mean Squared Error (MSE) quantifies the average squared error between predicted gene expression values $\hat{x}$ and ground-truth values $x$ across all $N$ nodes.


(5) \begin{equation*} {L}_{\mathrm{MSE}}=\frac{1}{N}{\sum}_{i=1}^N{\left({x}_i-{\hat{x}}_i\right)}^2 \end{equation*}

The Balanced Binary Cross-Entropy (BLCE), an improved variant of Binary Cross-Entropy (BCE), introduces a class weight α to address class imbalance (where positive edges are significantly fewer than negative ones). It quantifies the cross-entropy loss between predicted values $ \hat{y} $ and ground-truth labels $ y $ for all $ L $ edges. 


(6) \begin{equation*} {L}_{\mathrm{BLCE}}=-\frac{1}{L}{\sum}_{k=1}^L\left[{\alpha y}_k\cdot \log{\hat{y}}_k+\left(1-\alpha \right)\left(1-{y}_k\right)\cdot \log \left(1-{\hat{y}}_k\right)\right] \end{equation*}

Then the total loss function is:


(7) \begin{equation*} {L}_{\mathrm{total}}=\lambda{L}_{\mathrm{MSE}}+\left(1-\lambda \right){L}_{\mathrm{BLCE}} \end{equation*}

### Downstream tasks

The adjacency matrices for TF-TF, TF-CRE, CRE-CRE, and CRE-TG were updated using learned attention weights, and this new HRNet will serve as the basis for downstream task analysis.

### BioStreamNet for target gene expression prediction

BioStreamNet is a sparse FCNN module in SMOGT that employs an attention masking mechanism to predict TG expression based on TF expression and CRE accessibility (see details in Supplementary Note S4).

### BioStreamNet-based perturbation Modeling

We develop a perturbation modeling module based on BioStreamNet to validate the roles of identified regulators in cell fate. This module predicts expression changes in downstream TG, following the simulated knockout or overexpression of specific TFs and CREs (or combinations). (see details in Supplementary Note S5).

### MRWR_Pert for predicting transcription factor-perturbed genes

Inspired by the HuMMuS algorithm [17], we develop the MRWR_Pert module based on MRWR for predicting TF-perturbed TGs. The score of node ${v}_j$ at the $\left(t+1\right)$-th step, ${s}_j^{\left(t+1\right)}$ is defined as:


(8) \begin{equation*} {s}_j^{\left(t+1\right)}=\left(1-\beta \right)\mathrm{W}{s}_i^{(t)}+\beta{s}_0 \end{equation*}

where, ${v}_i,{v}_j\in V$,${s}_i^{(t)}$ is the node score at step $t$, $\mathbf{W}$ is the normalized network transition matrix (see details in Supplementary Note S6), and $\beta$ is the restart probability.

### MRWR_CTS for identifying driver regulators

MRWR_CTS is developed for identifying Driver regulators, including: (i) Driver TFs: Using differentially expressed genes (DEGs) as seed nodes, random walks start from the TG layer and end at the TF layer. The restart probability and transition probabilities are set identical to MRWR_Pert, and finally Driver scores for all TFs are obtained; (ii) Driver CREs: Using DEGs as seed nodes, random walks start from the TG layer and end at the CRE layer, with other settings consistent with Driver TFs.

### Identification of Co-CRE modules based on Louvain

Louvain_Co-CRE is based on the Louvain algorithm from igraph (R package) with default parameters. Following eNet 2.0 [36]: (i) Network hub CREs: CREs from the top N CRE-CRE pairs were selected as network hubs; (ii) Module hub CREs: In each CRE-CRE subnetwork, CREs from the top N pairs within the subnetwork were designated as module hubs.

### Evaluation metrics

Edge prediction accuracy was assessed via AUPR/AUC using ENCODE-derived TF-CRE and CRE-CRE interactions. GWAS, eQTL, SE, and TF perturbation enrichment scores were calculated following eNet2.0 methodology (see details in Supplementary Note S7).

## Results

### SMOGT accurately predicted transcriptional regulation and long-range CRE interactions by embedding epigenetic mechanism

Motivated by the observation that true regulatory relationships within a specific cellular context exhibit higher expression correlation [4, 5] (Supplementary Tables S4 and S5), we sought to establish a relation between statistical dependency and causal logic. SMOGT embedded epigenetic mechanisms into HGT and employed a semi-supervised strategy. This strategy relied on highly correlated TF-CRE and CRE-CRE pairs as pseudo-labels. We identified these pairs using a Pearson correlation coefficient (PCC) threshold that was optimized for each dataset (Supplementary Fig. S1 and Supplementary Note S8). This strategy proved effective, as SMOGT learned to assign significantly higher attention scores to validated interactions compared to other interactions in the prior network (Supplementary Fig. S2). To validate the advantage of the epigenetic mechanism-informed design, we compared SMOGT against a homogeneous version identical in architecture but omitting node-type and the epigenetic meta-path, observing SMOGT’s markedly higher accuracy in predicting true TF-CRE interactions (Supplementary Fig. S3). This result validated that a hierarchical epigenetic design is crucial for accuracy.

**Figure 2:**
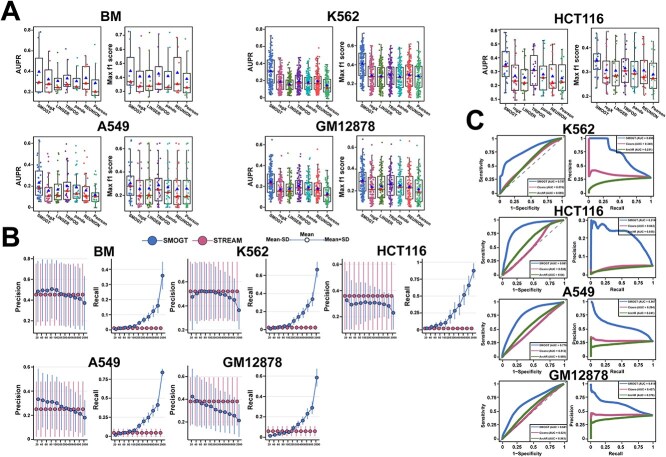
Comparison of SMOGT with other algorithms on network construction metrics across multiple datasets. (A) Comparison between SMOGT and other algorithms on AUPR of TF-CRE, where each point in the boxplot represents the performance of an algorithm on an individual TF. (B) Comparison of the accuracy and recall between SMOGT and STREAM in TF-CRE prediction. The x-axis represents different TopN pairs, and the y-axis shows the AUPR values across all TFs at each TopN threshold. The upper and lower ends of the bars indicate the mean ± standard deviation of AUPR values, respectively. (C) Comparison of SMOGT with other algorithms on AUC and AUPR metrics for CRE-CRE prediction across four cell line datasets. Red triangles represent the mean, and blue triangles indicate the median.

Using the TF-CRE relationships validated by ChIP-seq, we compared SMOGT with six algorithms (regX, LINGER, TRIPOD, Pando, REUNION, and Pearson; see details in Supplementary Note S9) via AUPR and maximal F1 score for each TF. The performance of each TF visualized in Supplementary Fig. S4 displayed the significant superiority of SMOGT in predicting TF-CRE binding (Fig. 2A). When the precision was calculated for top N pairs/TF, SMOGT outperformed STREAM in top200, top300, and top40 of BM, A549, and GM12878; in K562, SMOGT matched STREAM from top 60–200, but underperformed in HCT116. Notably, STREAM models only core motifs in JASPAR, leading to the high precision with the low recall due to the limited relationships (Fig. 2B).

Using CRE-CRE relations validated by Hi-C-seq, we also compared SMOGT with two sliding-window based co-CRE pair algorithms (Cicero and ArchR; see details in Supplementary Note S10) via AUC and AUPR across K562, HCT116, A549, and GM12878. Similarly, SMOGT outperformed both algorithms (Fig. 2C). These benchmarks confirm that epigenetical mechanism-guided HGT faithfully model both cis and trans regulations.

### HRNet constructed by SMOGT predicted TG expression by decoding TF perturbation logic

**Figure 3:**
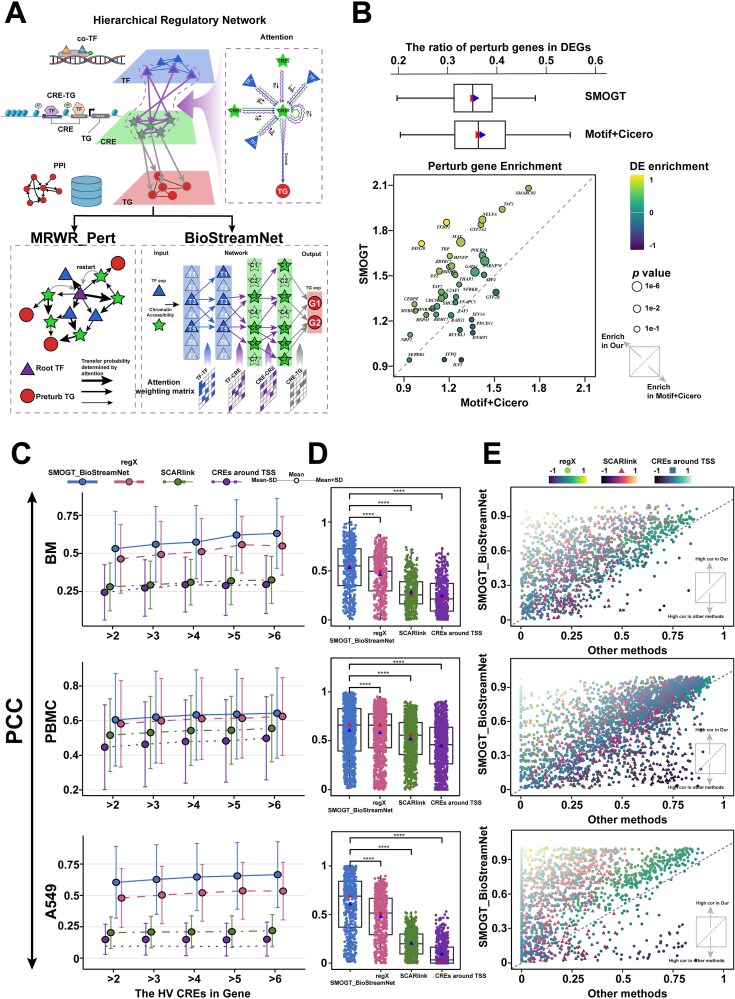
TF perturbation target identification and TG expression prediction based on SMOGT-constructed HRNet. (A) The framework of HRNet. (B) Enrichment of predicted perturbation genes (identified by MRWR) in DEGs, comparing the HRNet constructed by SMOGT to those by Motif+Cicero. Each point represents a TF perturbation, with x-axis for the enrichment values under Motif+Cicero conditions, and y-axis for SMOGT-based enrichment. Comparing the performance of TG expression prediction between SMOGT_BioStreamNet and other algorithms across BM, PBMC, and A549 datasets using PCC as the evaluation metric, presented in three formats. (C) Genes grouped by the number of HVCREs within ±25 Mb of their TSS, with bar plots showing mean PCC ± standard deviation per group. (D) PCC for genes with >2 HVCREs across all algorithms. (E) Scatter plots comparing SMOGT_BioStreamNet module with other algorithms, where each point represents a gene with x-axis for PCC of other method and y-axis for PCC of SMOGT_BioStreamNet.

Since TF perturbation (overexpression or deletion) not only directly regulates TG expression, but also exerts chromosomal long-range or even genomic-wide effects via 3D chromatin structure (CRE-CRE) and PPI [39, 40] [23, 24], SMOGT constructs HRNet by integrating attention-derived TF-CRE and CRE-CRE interactions with prior TF-TF and CRE-TG knowledge (Fig. 3A). In K562 CRISPR Perturb-seq data [41], when each perturbed TF was utilized as a seed node for MRWR_Pert on the HRNet, the consequent perturbed genes were significantly enriched in DEGs with the higher enrichment scores compared to the networks built from Motif-based TF-CRE or Cicero-based CRE-CRE interactions (Fig. 3B), indicating HRNet constructed by SMOGT could predict more potential perturbation targets.

Further, we used PCC to evaluate the capability of BioStreamNet in predicting TG expression (see details in Supplementary Note S11). Although the prediction performance changed insignificantly with the increasing network edges (Supplementary Fig. S5), the prediction accuracy was improved as the number of high variant-CREs directly connecting to TG was increased (Fig. 3C), indicating that the performance was impacted by feature selection rather than the number of network parameters. Using thresholds of TF-CRE: 0.8 and CRE-CRE: 2%, we compared SMOGT with regX and SCARlink across three datasets (BM, PBMC, and A549; see details in Supplementary Note S12), SMOGT showed the highest prediction accuracy in all datasets, followed by regX, SCARlink, and Transcription Start Site (TSS)-proximal CREs (PCC between TG expression and the average expression of CREs within 25 Mbp around the TSS of TGs; Fig. 3D and E). All the above results indicated that the SMOGT-constructed HRNet serves as a mechanistically-informed scaffold for predicting both TG expression and the logic of TF perturbations.

### SMOGT-constructed co-CRE module enriched disease loci and uncovered potential pathogenic mechanisms

**Figure 4:**
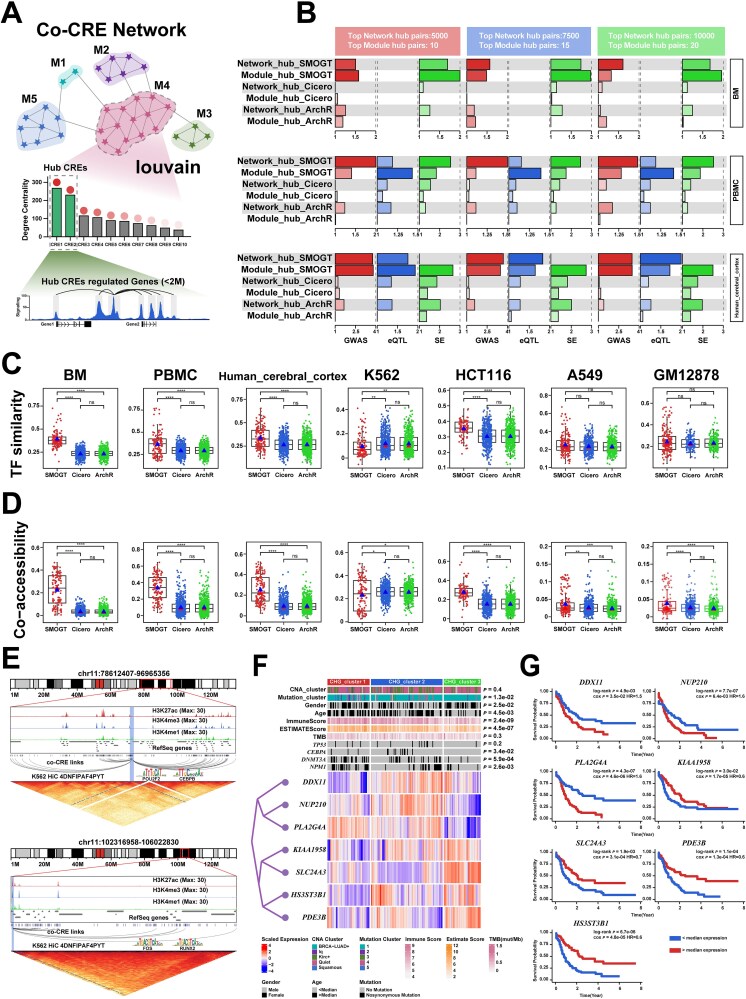
The framework of SMOGT co-CRE network and its biological applications in TCGA AML cohort. (A) The framework of SMOGT’s co-CRE network: Integrating module discovery and Hub CRE identification. (B) Comparative analysis on the enrichment of eNet2-defined Module_hub and Network_hub CREs in GWAS, eQTL and SE loci across SMOGT, Cicero and ArchR algorithms. (C) Comparison of TF-binding similarity in co-CRE subnetworks predicted by SMOGT, Cicero, and ArchR across seven datasets. (D) Comparison of co-accessibility in co-CRE subnetworks predicted by SMOGT, Cicero and ArchR across seven datasets. (E) Epigenomic and 3D genome browser visualization of long-range regulatory relationships and motif enrichment for two Hub_CREs. (F) Heatmap visualization of TCGA AML dataset clustering based on CHG. (G) The relationship between HCG and prognosis in the TCGA AML dataset.

CREs are recognized as hallmarks of diseases and tumors, because they enrich SNPs and eQTLs in noncoding regions [42–44]. Further studies have shown that disease-associated CREs were often clustered in networks [36]. Inspired by prior work using multi-view clustering to identify gene modules from multi-omics data [11], we focused on identifying co-CRE modules. Although Huang *et al.* proposed eNet 2.0 for co-CRE identification based on expression similarity [36], its application of fixed genomic windows constrained the accuracy on disease-associated CREs. Thus, we hypothesize that the context-specific CRE embeddings learned by SMOGT could identify co-CREs and Hub CREs more accurately (Fig. 4A). When compared to the top N globally correlated CRE-pairs (referred to as Network_Hub, consistent with eNet 2.0) identified by Cicero and ArchR, Network_Hubs in SMOGT showed the highest enrichment scores for GWAS and eQTL loci (Fig. 4B, red/blue bars), indicating that the Network_Hub in SMOGT was closer to the central hub of disease progression. Thereby, Network_Hub in SMOGT also showed the highest enrichment for SE (super enhancers) compared to Cicero and ArchR (Fig. 4B, green bars). Next, we used Louvain to detect co-CRE sub-networks in the global CRE-CRE networks constructed by above three algorithms. Because of the absence of window constraints, SMOGT exhibited the fewer sub-networks, the larger CRE intervals and the lower density (Supplementary Table S6), which benefited the prediction of long-range regulation. For Module_Hubs (top N pairs per subnetwork, as in eNet 2.0), SMOGT-derived Module_Hubs exhibited the highest GWAS, eQTL, and SE enrichment (Fig. 4B, second-row bars), suggesting the capability of both capturing long-range interactions and enriching disease loci. To uncover the biological significance, we compared TF binding site similarity in sub-networks across seven datasets (Fig. 4C). SMOGT sub-networks showed the significantly highest similarity in BM, PBMC, Human_cerebral_cortex, and HCT116. Although insignificant, similar trends were observed in A549 and GM12878, except the lower similarity in K562. Since the similarity in TF binding sites suggested a coordinated chromatin accessibility opening (co-accessibility) among CREs, we further compared co-accessibility within sub-networks to confirm the prediction accuracy on disease-associated CREs. Consistent with the TF binding site similarity patterns, SMOGT-identified sub-networks exhibited the significantly higher co-accessibility of CREs than those from Cicero and ArchR (Fig. 4D).

To determine whether SMOGT-identified CRE-CRE subnetworks could serve as prognostic markers in cancer, we examined leukemia [45], a disease marked by abundant noncoding mutations. Core CREs in subnetworks were selected by degree centrality ranking, with top N CREs recognized as Hub_CREs (Fig. 4A). Consistent with prior findings [46], two noncoding Hub_CREs on chr11 (chr11–88523348-88 524226 and chr11–102346711-102347590) with high histone signals were visualized, illustrating the regulation not only on the nearest neighbors, but also to distant locations (>2 M). Notably, these Hub_CREs showed the significant enrichment for oncogene (POU2F2, CEBPB, FOS, RUNX2) motifs (Fig. 4E).

Expanding to all Hub_CREs, genes within 2 Mbp around Hub_CREs are defined as Hub CRE-regulated Genes (HCGs). LASSO regression identified seven prognostic HCGs (DDX11, NUP210, PLA2G4A, KIAA1958, SLC24A3, HS3ST3B1, PDE3B). Hierarchical clustering of TCGA AML based on these HCGs yielded three clusters significantly overlapping with mutation-based clustering: Cluster 1 with elevated ImmuneScore (ESTIMATE) and enriched DNMT3A/NPM1 mutations; Cluster 2 with enriched TP53/CEBPA mutations (Fig. 4F). Survival analysis identified DDX11, NUP210, and PLA2G4A as risk factors, while KIAA1958, SLC24A3, PDE3B, and HS3ST3B1 as protective factors (Fig. 4G). These findings validate that HRNet generates biological CRE embeddings that are rich with 3D genomic and long-range context. This ability is foundational, as it ensures the reliability of cell fate modeling.

### SMOGT enabled precise and comprehensive identification of driver regulators in cell lineage branching

**Figure 5:**
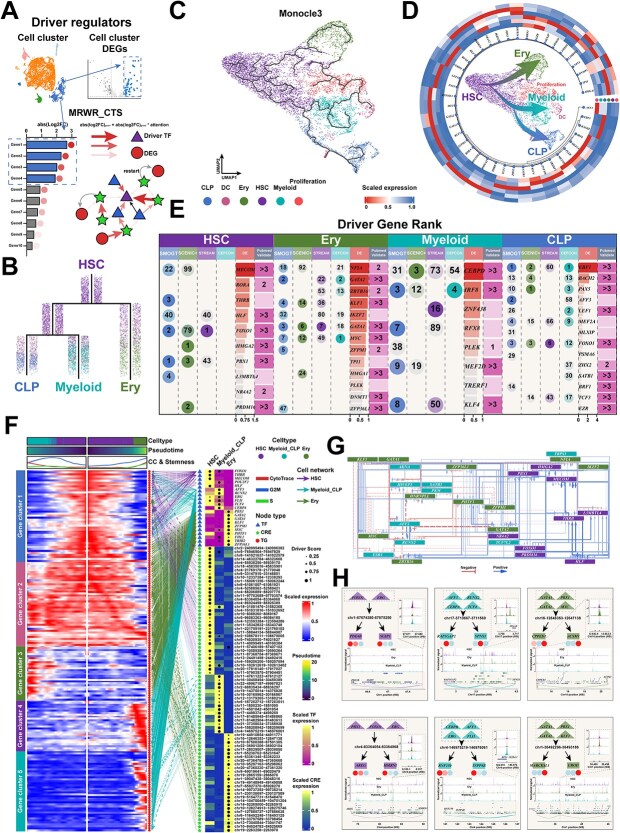
The SMOGT framework for driver regulator identification and its application in BM Lineage commitment. (A) The SMOGT framework for driver regulator identification consists of two key components: (i) cell type DEG identification, and (ii) cell type-specific MRWR. The BM lineage trajectories were constructed using two distinct approaches: (B) URD and (C) Monocle3. For visualization clarity, the trajectory lines in scVelo are uniformly rendered with consistent thickness to enhance prominence. (D) The outer circular diagram displays cell type-specific TFs, while the inner schematic illustrates lineage differentiation trajectories. (E) Comparative analysis of SMOGT, SCENIC+, STREAM and CEFCON in identifying lineage-driving regulators for BM differentiation. Numeric values represent the ranking scores of predicted driver regulators for each lineage, with the darker intensity indicating the higher ranking. (F) The trajectory composite plots of HSC built on lineage-specific gene expression bifurcated into Myeloid_CLP and Ery lineages. Top: Cell classification along pseudo time with associated stemness scores and cell cycle phase scores; bottom left: Pseudo temporal heatmap of gene expression; bottom center: Cell type-specific HRNet; bottom right: SMOGT-identified driver regulators and CRE driver scores (represented by dot size) with corresponding expression heatmaps (encoded by color intensity around dots). (G) Cis-regulatory network of the HSC bifurcation into Myeloid_CLP and Ery lineages, with all TFs. This panel focuses on the regulatory relationships among TFs, presenting a hierarchical network composed solely of TFs. It omits the CREs to clearly depict the complex cooperative and feedback circuits formed. (H) Epigenomic and 3D genome browser views of *trans*-regulatory relationships during HSC bifurcation into Myeloid_CLP and Ery lineages. This panel illustrates a concrete case where a TF, after binding to a CRE, regulates a distantly located target gene through 3D chromatin structure.

Using the BM niche dataset (with defined lineage trajectories and experimentally validated driver regulators), we compared the MRWR_CTS module of SMOGT with three SOTA algorithms (SCENIC+, CEFCON and STREAM; see details in Supplementary Note S13) in terms of their capability of inferring lineage-driving regulators (Fig. 5A). Differentiation trajectories of Hematopoietic Stem Cells (HSCs) in BM were reconstructed using URD algorithm. HSCs either maintained self-renewal (proliferation cluster) or differentiated into two branches: a common progenitor for myeloid and lymphoid cells (Myeloid_CLP) and erythroid lineage (Ery), with Myeloid_CLP further splitting into Myeloid and CLP (Fig. 5B). Such hierarchical lineage specification validated by previous studies [38, 47] was recapitulated using Monocle3 (Fig. 5C).

Cluster-specific TFs (log2FC > 0.5, FDR < 0.01, pct1-pct2 > 0.3) were visualized to provide the criteria for driver regulator identification (Fig. 5D). For HSC and its three offspring lineages (Ery, Myeloid, and CLP), lineage-specific TFs (namely, driver regulators) were ranked by log2FC as the benchmark metric. The four methods showed an intensive overlap in the predicted driver regulators across lineages, suggesting the conserved regulatory mechanisms in HSCs (Fig. 5E). Among the SMOGT’s top 5 driver TF scores in HSC lineage, four were HSC-specific TFs, and 2 were *ex vivo* validated by three PubMed studies. In contrast, SCENIC+ and STREAM had three and 1 specific TFs in their top 5, respectively, while others ranked lower. In Ery lineage, the SMOGT’s top 10 driver TFs included five specific TFs (4 validated by three PubMed studies), whereas only two HSC-specific TFs were included in the top 10 of CEFCON, 1 of STREAM and 1 of SCENIC+. Similarly, in Myeloid lineage, the SMOGT’s top 10 included four specific TFs (3 validated), and CEFCON and SCENIC+ only contained 1, respectively. In CLP lineage, the SMOGT’s top 10 included five specific TFs (3 validated), compared to the four in the top 10 by SCENIC+ and CEFCON (4 and three validated, respectively), and the only 1 by STREAM. These results robustly suggested that SMOGT accurately reconstructed lineage-specific regulatory networks and identified their driver regulators (Supplementary Note S14).

Using SMOGT’s node embeddings, we constructed and visualized the regulatory network governing HSC bifurcation into Ery and Myeloid_CLP lineages (Fig. 5F, top). Along the trajectory from HSC to Myeloid_CLP, S phase scores remained low while G2M scores were transiently increased, and stemness (CytoTrace) scores increased persistently, suggesting that Myeloid_CLP progressively exited cell cycle for differentiation. In contrast, the trajectory from HSC towards Ery showed the continuous increases in S/G2M phase and stemness scores, suggesting that Ery retained the proliferative capacity during differentiation. These consequences revealed the distinct regulatory modes controlling temporal gene expression, as k-means clustering segregated genes into five clusters: HSC-specific (cluster 1), Myeloid_CLP-specific (cluster 3), Ery-specific (clusters 4–5), and mixed pattern (cluster 2) (Fig. 5F, left).

We constructed a TF-CRE-TG regulatory network to comprehensively map lineage-specific regulations from HSC to Myeloid_CLP/Ery (Fig. 5F, right). Trans-regulatory patterns (including *cis*-element looping) were characterized by visualizing TF-CRE-TF triads (Fig. 5G), where lineage-specific TFs were clustered hierarchically. In *trans*-regulation (long-range interactions), HSC stemness factors FOXO1 and ERG (implicated in leukemia initiation) [48, 49] regulated growth factor AREG and vascular secreted protein MMRN1 via distal CREs. In Myeloid_CLP, WNT pathway effector TCF4 and pioneer factor CEBPA controlled membrane transporters SPNS3 and RNF150 through trans-acting elements (Fig. 5H). In summary, by resolving the cis- and trans-regulatory logic within HRNet, SMOGT establishes a mechanistic link between HRNet and the precise identification of driver TFs.

### SMOGT elucidates the hierarchical causality of driver TFs and CREs in shaping cell fate plasticity via perturbation Modeling

To validate the functional roles of identified regulators in cell fate, we employed BioStreamNet module to simulate the knocking out and overexpression of specific TFs and CREs, observing the alterations in the direction and magnitude of cell fate (see details in Supplementary Note S5).

**Figure 6:**
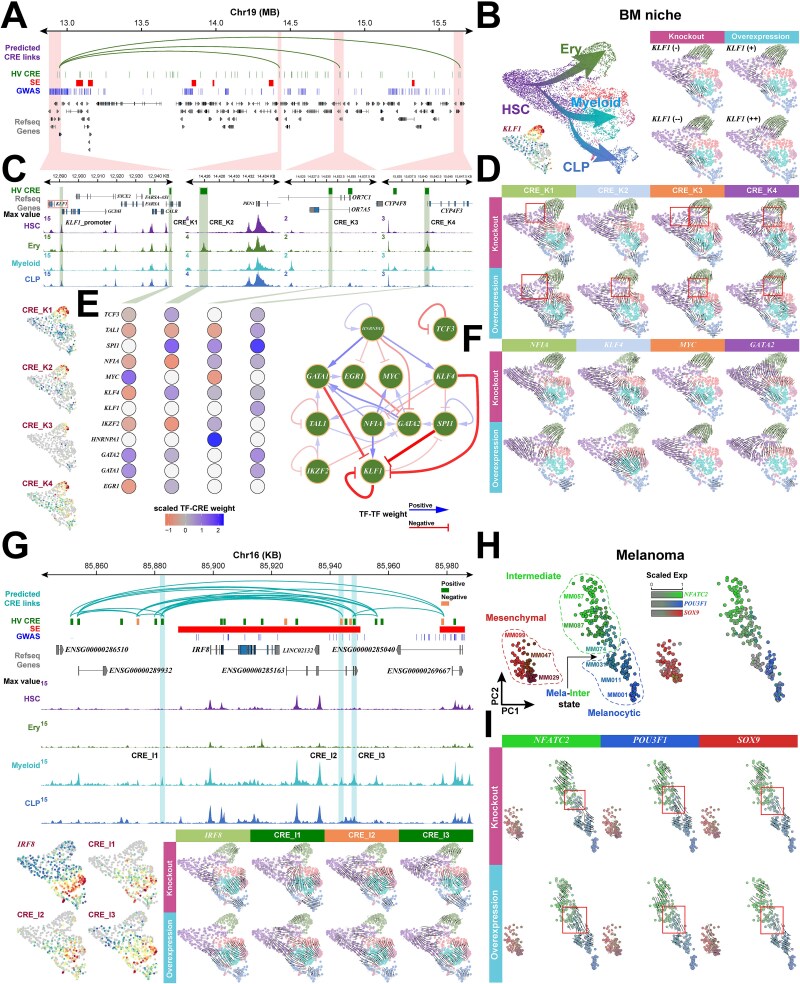
SMOGT perturbation modeling of driver regulators in hematopoietic trajectories and melanoma EMT. (A) Genomic visualization of the CRE-TG and CRE-CRE layer in KLF1-specific HRNet. (B) Perturbation vector field plots illustrating predicted cell fate shifts in the BM niche following *in silico* knockout and overexpression of KLF1. (C) A detailed view of the KLF1 locus showing four distal CREs (CRE_K1–4) and their chromatin accessibility across hematopoietic lineages. (D) Perturbation vector field plots for the simulated knockout and overexpression of each of the CRE_K1–4. (E) The regulatory subnetwork of 12 TFs predicted to act on CRE_K1–4, showing TF-CRE weights (heatmap) and the TF-TF interaction network (diagram). (F) Perturbation vector field plots for the perturbation of four key TFs within the KLF1 regulatory circuit. (G) Perturbation analysis of the myeloid regulator IRF8. The top part shows its genomic locus with associated CREs. The bottom part shows the vector field plots from perturbing IRF8 and its CREs. (H) The UMAP plot shows melanoma cells with three states and a transitional Mela-Interstate. Below are the perturbation vector field plots of three marker TFs.

In the BM niche, KLF1 is a key regulator of the Ery lineage (see details in Supplementary Note S14). We constructed the KLF1-specific HRNet using connections with an absolute weight > 0.15 from its expression prediction model (Fig. 6A). In our perturbation modeling, progressively decreasing KLF1 expression from 0.5-fold to complete knocking out increasingly inhibited HSC differentiation towards the Ery fate, biasing it towards the Myeloid-CLP fate. Conversely, increasing its expression from 2-fold to 3-fold progressively attenuated the potential for Myeloid-CLP differentiation while enhancing the commitment to the Ery fate (Fig. 6B). Further investigation revealed that KLF1’s differential expression was not directly associated with its promoter’s accessibility (Fig. 6C), but was distally regulated by four CREs within a 2Mbp upstream region (chr19–12947685-12948558, chr19–14425413-14426334, chr19–14830478-14831287 and chr19–15640334-15641191, termed CRE_K1–4). Among these, CRE_K1 acts as a hub, connecting to CRE_K2–4 (Fig. 6C). Perturbation modeling showed that while knocking out or overexpression of any of the four CREs caused the Ery lineage to exit its original fate, their impacts on the entire HSC lineage tree were markedly different (Fig. 6D). knocking out the hub CRE (CRE_K1) relocated the lineage root to an Ery progenitor state, which then differentiated towards HSC, Proliferation, and Myeloid fates (Fig. 6D, CRE_K1 knocking out). However, overexpressing CRE_K1 did not drive differentiation towards any specific lineage; instead, it created a circular vector field at the Ery progenitor stage. This suggests that CRE_K1 overexpression does not converge to a single attractor but induces a periodic oscillation in an undetermined progenitor state (Fig. 6D, CRE_K1 Overexpression, red box), an effect similarly observed upon CRE_K3 knocking out. In contrast, knocking out CRE_K2 strongly biased HSCs towards proliferation. Yet, overexpressing CRE_K2 or perturbing CRE_K3 and CRE_K4 attracted cells to the junction of the HSC, Proliferation and Ery lineages (Fig. 6D, red boxes). The perturbations of CRE_K1-4 revealed functional specialization among them, suggesting cooperative and distinct roles for their binding TFs. These four CREs are predicted to be regulated by 12 upstream TFs. Intriguingly, the pattern of TF regulation shifts from predominantly inhibitory on CRE_K1 to predominantly activating on CRE_K4, forming a complex circuit with multiple positive and negative feedback loops (Fig. 6E). Within this circuit, NFIA directly activated while KLF4 directly inhibited KLF1. As expected, knocking out NFIA suppressed Ery differentiation, biasing HSCs toward the Myeloid lineage, whereas perturbing KLF4 had the opposite effect (Fig. 6F). Perturbation of MYC, a downstream target activated by NFIA, yielded effects similar to NFIA perturbation. Furthermore, because GATA2 exerted an indirect inhibitory effect on KLF1 via GATA1, its overexpression did not fully commit HSCs to the Ery fate but instead drove them to an Ery progenitor stage.

To further resolve the functions of CREs in cell fate, we focused on IRF8, a key regulator of the Myeloid lineage located within a SE region (see details in Supplementary Note S14, Fig. 6G top). Silico perturbation modeling showed that IRF8 knockout caused Myeloid lineage to exit their fate and arrest in a proliferative state. Conversely, its overexpression significantly enhanced HSC commitment toward the Myeloid lineage (Fig. 6G bottom). We then investigated specific CREs within the IRF8 HRNet (constructed as with KLF1): two downstream CREs located within the SE (chr16–85943448-85944255 and chr16–85947801-85948725, termed CRE_I2 and CRE_I3) and the nearest upstream CRE (chr16–85882146-85883060, termed CRE_I1). Based on the weights in the CRE-TG layer, we classified these CREs as either positive or negative regulators (Fig. 6G middle). Knocking out the positive CREs (CRE_I1 and I3) led to a loss of Myeloid fate with a bias toward proliferation, whereas their overexpression maintained the Myeloid fate and guided the proliferation cells toward it. However, both knockout and overexpression of the negative regulator CRE_I2 produced lineage alterations similar to overexpression of the positive CREs, reflecting a logic dominated by positive feedback in the regulatory network during fate specification [37443338]. This deconstruction of the IRF8-specific HRNet dissected the distinct functions of its internal positive and negative regulatory CREs in driving cell fate.

Cell fate plasticity is critically governed at transitional states. We therefore leveraged SMOGT to dissect the influence of fate regulators on the epithelial-to-mesenchymal transition (EMT) in melanoma. The melanoma dataset was composed of three clusters: Intermediate, Melanocytic, and Mesenchymal cells, with a ‘Mela-Interstate’ subpopulation, representing cells in early EMT, forming at the junction between the Melanocytic and Intermediate cells (Fig. 6H). Silico perturbation modeling revealed that knocking out the Intermediate marker TF, NFATC2, significantly reduced the vector field strength at the Mela-Interstate, indicating a weakened transition from the Melanocytic to the Intermediate state. Conversely, overexpressing NFATC2 enhanced this transition (Fig. 6H, red box for NFATC2). Perturbing the Melanocytic marker TF, POU3F1, produced an opposite trend, although the effect was weaker than that of NFATC2 perturbation (Fig. 6H, red box for POU3F1). In contrast, perturbing the Mesenchymal marker TF, SOX9, did not cause any significant change in the vector field strength (Fig. 6H, red box for SOX9). These results suggest that the optimal therapeutic window for intervening in tumor EMT is during the initial transition; intervention at the terminal mesenchymal state can no longer reverse the process.

In summary, SMOGT’s perturbation modeling not only validates the causal functions of driver regulators but also reveals the functional specialization and hierarchical regulatory mechanisms of different elements within their regulatory modules in both normal development and cancer.

## Conclusion

This study introduces SMOGT to address a key limitation in regulatory networks modeling: inferring the causal logic that governs cell fate. We move beyond conventional single-layer regulatory network by constructing a HRNet that models the multi-layer regulation in epigenetics. This causal framework facilitates the transition from mechanistic interpretation to predictive modulation of cell fate, thereby delineating the logic that governs lineage commitment, identifying therapeutic windows and assigning prognostic value to noncoding elements. The intra-cellular causal networks constructed by SMOGT offer a valuable framework for exploring future research frontiers. These include the integration of cell–cell communication models and the incorporation of spatial multi-omics data, which are critical steps toward the construction of a mechanistic virtual cell.

**Key Points**

- We introduce SMOGT, a Heterogeneous Graph Transformer that constructs a Hierarchical Regulatory Network (HRNet) by embedding the multi-layered flow of epigenetic information.
- SMOGT demonstrates superior accuracy in predicting both transcriptional regulation (TF-CRE) and long-range chromatin interactions (CRE-CRE) when validated against benchmark datasets.
- The framework’s HRNet enables robust identification of driver regulators and *in silico* perturbation modeling, providing a mechanistic tool to dissect and predict cell fate.

## Supplementary Material

Supplementary_Material_bbaf664

Supplementary_Figure_S1_bbaf664

Supplementary_Figure_S2_bbaf664

Supplementary_Figure_S3_bbaf664

Supplementary_Figure_S4_bbaf664

Supplementary_Figure_S5_bbaf664

Supplymentary_Table1_bbaf664

Supplymentary_Table2_bbaf664

Supplymentary_Table3_bbaf664

Supplymentary_Table4_bbaf664

Supplymentary_Table5_bbaf664

Supplymentary_Table6_bbaf664

## Data Availability

The detailed descriptions of data types, sources, and related information are provided in the Materials and Methods section. The source code and application cases have been uploaded to GitHub (https://github.com/YuHongHuang-lab/SMOGT).

## References

[1] Noutahi E, Hartford J, Tossou P. et al. Virtual cells: Predict, Explain, *Discover*. *arXiv* 2025;2505.14613. https://arxiv.org/abs/2505.14613

[2] Bunne C, Roohani Y, Rosen Y. et al. How to build the virtual cell with artificial intelligence: Priorities and opportunities. *Cell* 2024;187:7045–63. 10.1016/j.cell.2024.11.015 39672099 PMC12148494

[3] Loers JU, Vermeirssen V. A single-cell multimodal view on gene regulatory network inference from transcriptomics and chromatin accessibility data. *Brief Bioinform* 2024;25:bbae382. 10.1093/bib/bbae382 PMC11359808 39207727

[4] Badia IMP, Wessels L, Muller-Dott S. et al. Gene regulatory network inference in the era of single-cell multi-omics. *Nat Rev Genet* 2023;24:739–54. 10.1038/s41576-023-00618-5 37365273

[5] Kim D, Tran A, Kim HJ. et al. Gene regulatory network reconstruction: Harnessing the power of single-cell multi-omic data. *NPJ Syst Biol Appl* 2023;9:51. 37857632 10.1038/s41540-023-00312-6 PMC10587078

[6] Liu C, Ding S, Kim HJ. et al. Multitask benchmarking of single-cell multimodal omics integration methods. *Nat Methods* 2025;22:2449–60. 10.1038/s41592-025-02856-3 PMC12615258 41083898

[7] Lee MYY, Li M. Integration of multi-modal single-cell data. *Nat Biotechnol* 2024;42:190–1. 10.1038/s41587-023-01826-4 37231264

[8] Gayoso A, Steier Z, Lopez R. et al. Joint probabilistic modeling of single-cell multi-omic data with totalVI. *Nat Methods* 2021;18:272–82. 10.1038/s41592-020-01050-x 33589839 PMC7954949

[9] Ashuach T, Gabitto MI, Koodli RV. et al. MultiVI: Deep generative model for the integration of multimodal data. *Nat Methods* 2023;20:1222–31. 10.1038/s41592-023-01909-9 37386189 PMC10406609

[10] Argelaguet R, Arnol D, Bredikhin D. et al. MOFA+: A statistical framework for comprehensive integration of multi-modal single-cell data. *Genome Biol* 2020;21:111. 32393329 10.1186/s13059-020-02015-1 PMC7212577

[11] Gao X, Wang Y, Hou W. et al. Multi-view clustering for integration of gene expression and methylation data with tensor decomposition and self-representation learning. *IEEE/ACM Trans Comput Biol Bioinform* 2023;20:2050–63. 10.1109/TCBB.2022.3229678 37015414

[12] Hao Y, Stuart T, Kowalski MH. et al. Dictionary learning for integrative, multimodal and scalable single-cell analysis. *Nat Biotechnol* 2024;42:293–304. 10.1038/s41587-023-01767-y 37231261 PMC10928517

[13] Wu W, Zhang W, Ma X. Network-based integrative analysis of single-cell transcriptomic and epigenomic data for cell types. *Brief Bioinform* 2022;23:bbab546. 10.1093/bib/bbab546 35043143

[14] Kartha VK, Duarte FM, Hu Y. et al. Functional inference of gene regulation using single-cell multi-omics. *Cell Genom* 2022;2:100166. 10.1016/j.xgen.2022.100166 36204155 PMC9534481

[15] Jiang Y, Harigaya Y, Zhang Z. et al. Nonparametric single-cell multiomic characterization of trio relationships between transcription factors, target genes, and cis-regulatory regions. *Cell Syst* 2022;13:e734. 10.1016/j.cels.2022.08.004 PMC9509445 36055233

[16] Bravo Gonzalez-Blas C, De Winter S, Hulselmans G. et al. SCENIC+: Single-cell multiomic inference of enhancers and gene regulatory networks. *Nat Methods* 2023;20:1355–67. 10.1038/s41592-023-01938-4 37443338 PMC10482700

[17] Trimbour R, Deutschmann IM, Cantini L. Molecular mechanisms reconstruction from single-cell multi-omics data with HuMMuS. *Bioinformatics* 2024;40:btae143. 10.1093/bioinformatics/btae143 PMC11065476 38460192

[18] Chen X, Wang Y, Cappuccio A. et al. Mapping disease regulatory circuits at cell-type resolution from single-cell multiomics data. *Nat Comput Sci* 2023;3:644–57. 10.1038/s43588-023-00476-5 37974651 PMC10653299

[19] Zhang S, Pyne S, Pietrzak S. et al. Inference of cell type-specific gene regulatory networks on cell lineages from single cell omic datasets. *Nat Commun* 2023;14:3064. 37244909 10.1038/s41467-023-38637-9 PMC10224950

[20] Li Y, Ma A, Wang Y. et al. Enhancer-driven gene regulatory networks inference from single-cell RNA-seq and ATAC-seq data. *Brief Bioinform* 2024;25:bbae369. 10.1093/bib/bbae369 PMC11289686 39082647

[21] Yang Y, Pe'er D. REUNION: Transcription factor binding prediction and regulatory association inference from single-cell multi-omics data. *Bioinformatics* 2024;40:i567–75. 10.1093/bioinformatics/btae234 38940155 PMC11211829

[22] Yuan Q, Duren Z. Inferring gene regulatory networks from single-cell multiome data using atlas-scale external data. *Nat Biotechnol* 2025;43:247–57. 10.1038/s41587-024-02182-7 38609714 PMC11825371

[23] Xi X, Li J, Jia J. et al. A mechanism-informed deep neural network enables prioritization of regulators that drive cell state transitions. *Nat Commun* 2025;16:1284. 10.1038/s41467-025-56475-9 39900922 PMC11790924

[24] Tang T, Zhang X, Liu Y. et al. Machine learning on protein-protein interaction prediction: Models, challenges and trends. *Brief Bioinform* 2023;24:bbad076. 10.1093/bib/bbad076 36880207

[25] Szklarczyk D, Kirsch R, Koutrouli M. et al. The STRING database in 2023: Protein-protein association networks and functional enrichment analyses for any sequenced genome of interest. *Nucleic Acids Res* 2023;51:D638–46. 10.1093/nar/gkac1000 36370105 PMC9825434

[26] Novakovsky G, Dexter N, Libbrecht MW. et al. Obtaining genetics insights from deep learning via explainable artificial intelligence. *Nat Rev Genet* 2023;24:125–37. 10.1038/s41576-022-00532-2 36192604

[27] Hao M, Gong J, Zeng X. et al. Large-scale foundation model on single-cell transcriptomics. *Nat Methods* 2024;21:1481–91. 10.1038/s41592-024-02305-7 38844628

[28] Consens ME, Dufault C, Wainberg M. et al. Transformers and genome language models. *Nature Machine Intelligence* 2025;7:346–62. 10.1038/s42256-025-01007-9

[29] Wang P, Wen X, Li H. et al. Deciphering driver regulators of cell fate decisions from single-cell transcriptomics data with CEFCON. *Nat Commun* 2023;14:8459. 38123534 10.1038/s41467-023-44103-3 PMC10733330

[30] Ma A, Wang X, Li J. et al. Single-cell biological network inference using a heterogeneous graph transformer. *Nat Commun* 2023;14:964. 10.1038/s41467-023-36559-0 36810839 PMC9944243

[31] Xu J, Lu C, Jin S. et al. Deep learning-based cell-specific gene regulatory networks inferred from single-cell multiome data. *Nucleic Acids Res* 2025;53:gkaf138. 10.1093/nar/gkaf138 PMC11879466 40037709

[32] Zitnik M, Li MM, Wells A. et al. Current and future directions in network biology. *Bioinform Adv* 2024;4:vbae099. 10.1093/bioadv/vbae099 39143982 PMC11321866

[33] Kamimoto K, Stringa B, Hoffmann CM. et al. Dissecting cell identity via network inference and in silico gene perturbation. *Nature* 2023;614:742–51. 10.1038/s41586-022-05688-9 36755098 PMC9946838

[34] De Domenico M . More is different in real-world multilayer networks. *Nat Phys* 2023;19:1247–62. 10.1038/s41567-023-02132-1

[35] Baptista A, Gonzalez A, Baudot A. Universal multilayer network exploration by random walk with restart. *Commun Phys* 2022;5:170. 10.1038/s42005-022-00937-9

[36] Lin H, Ye X, Chen W. et al. Modular organization of enhancer network provides transcriptional robustness in mammalian development. *Nucleic Acids Res* 2025;53:gkae1323. 10.1093/nar/gkae1323 PMC11736433 39817516

[37] Persad S, Choo ZN, Dien C. et al. SEACells infers transcriptional and epigenomic cellular states from single-cell genomics data. *Nat Biotechnol* 2023;41:1746–57. 10.1038/s41587-023-01716-9 36973557 PMC10713451

[38] Setty M, Kiseliovas V, Levine J. et al. Characterization of cell fate probabilities in single-cell data with Palantir. *Nat Biotechnol* 2019;37:451–60. 10.1038/s41587-019-0068-4 30899105 PMC7549125

[39] Tan Y, Xie L, Yang H. et al. BioDSNN: A dual-stream neural network with hybrid biological knowledge integration for multi-gene perturbation response prediction. *Brief Bioinform* 2024;26:bbae617. 10.1093/bib/bbae617 PMC11586784 39584702

[40] Bai D, Ellington CN, Mo S. et al. AttentionPert: Accurately modeling multiplexed genetic perturbations with multi-scale effects. *Bioinformatics* 2024;40:i453–61. 10.1093/bioinformatics/btae244 38940174 PMC11211811

[41] Replogle JM, Norman TM, Xu A. et al. Combinatorial single-cell CRISPR screens by direct guide RNA capture and targeted sequencing. *Nat Biotechnol* 2020;38:954–61. 10.1038/s41587-020-0470-y 32231336 PMC7416462

[42] Puente XS, Bea S, Valdes-Mas R. et al. Non-coding recurrent mutations in chronic lymphocytic leukaemia. *Nature* 2015;526:519–24. 10.1038/nature14666 26200345

[43] Elliott K, Larsson E. Non-coding driver mutations in human cancer. *Nat Rev Cancer* 2021;21:500–9. 10.1038/s41568-021-00371-z 34230647

[44] Chen H, Li C, Peng X. et al. A pan-cancer analysis of enhancer expression in nearly 9000 patient samples. *Cell* 2018;173:386–399.e12. https://doi:10.1016/j.cell.2018.03.027 10.1016/j.cell.2018.03.027 PMC5890960 29625054

[45] Boutzen H, Murison A, Oriecuia A. et al. Identification of leukemia stem cell subsets with distinct transcriptional, epigenetic and functional properties. *Leukemia* 2024;38:2090–101. 10.1038/s41375-024-02358-9 39169113 PMC11436360

[46] Chen Z, Snetkova V, Bower G. et al. Increased enhancer-promoter interactions during developmental enhancer activation in mammals. *Nat Genet* 2024;56:675–85. 10.1038/s41588-024-01681-2 38509385 PMC11203181

[47] Faure L, Soldatov R, Kharchenko PV. et al. scFates: A scalable python package for advanced pseudotime and bifurcation analysis from single-cell data. *Bioinformatics* 2023;39:btac746. 10.1093/bioinformatics/btac746 PMC9805561 36394263

[48] Subramanian S, Thoms JAI, Huang Y. et al. Genome-wide transcription factor-binding maps reveal cell-specific changes in the regulatory architecture of human HSPCs. *Blood* 2023;142:1448–62. 10.1182/blood.2023021120 37595278 PMC10651876

[49] Peng L, Tang S, Li H. et al. Angelica sinensis polysaccharide suppresses the aging of hematopoietic stem cells through Sirt1/FoxO1 Signaling. *Clin Lab* 2022;68. 10.7754/Clin.Lab.2021.210731 35536062

