# Supplementary material for: Deciphering hierarchical regulatory network of cell fate via an epigenetics-informed heterogeneous graph transformer on single-cell multi-omics data
Source: Brief Bioinform. 2025 Dec 12;26(6):bbaf664. doi: 10.1093/bib/bbaf664 (PMC12875533; doi:10.1093/bib/bbaf664)
Supplement: Supplementary_Material_bbaf664 [file supplementary_material_bbaf664.docx]

# **Supplementary Material**

**Supplementary Table S1. Summary of Datasets used in SMOGT**

**Supplementary Table S2. The true TF-CRE relationships recorded in the ENCODE and ChIP-Altas databases**

**Supplementary Table S3. The true CRE-CRE relationships recorded in the ENCODE**

**Supplementary Table S4. The numbers of gene and CRE involved in the computation within the benchmark dataset**

**Supplementary Table S5. Proportions of prior edges versus true edges under different PCC thresholds**

**Supplementary Table S6. Statistical Characterization of co-CRE Subnetworks Computed by SMOGT, Cicero, and ArchR**

**Supplementary Figure S1.** The performance of SMOGT on TF-CRE accuracy and TG construction across different PCC thresholds. **(A)** SMOGT’s average AUPR values across different PCC thresholds. The average AUPR refers to the mean AUPR calculated across all TFs. **(B)** Similarity between SMOGT-reconstructed TG profile and original TG expression profile across different PCC thresholds, measured by average PCC of all TG. The left panel shows the metrics over all epochs, while the right panel displays the trend of Metrics as epochs increase.

**Alt Text:** The figure consists of two panels. Panel A contains two line graphs. The left graph plots the Average AUPR on the y-axis against different PCC thresholds on the x-axis. The right graph plots the Average AUPR on the y-axis against the number of Epochs on the x-axis. Panel B contains two line graphs. The left graph plots the Average PCC on the y-axis against different PCC thresholds on the x-axis. The right graph plots the PCC on the y-axis against the number of Epochs on the x-axis.

**Supplementary Figure S2.** Attention scores of edges in the prior network versus edges in the true network across different datasets.

**Alt Text:** The figure is a panel of ten boxplots arranged in two rows (labeled TF-CRE and CRE-CRE) across five datasets. Each plot compares a blue box (left) with a pink box (right). In all ten graphs, the pink boxes are positioned consistently higher than the blue boxes, with brackets and asterisks indicating a significant difference between the two distributions.

**Supplementary Figure S3.** Comparison between the homogeneous and heterogeneous model versions of SMOGT.

**Alt Text:** The figure consists of two panels. Panel A is a boxplot comparing two categories labeled homogeneous and heterogeneous. The pink boxplot is positioned significantly higher on the y-axis. Panel B is a line graph tracking performance over 70 epochs. The pink line consistently remains at a high level throughout the x-axis span.

**Supplementary Figure S4.** Comparison of SMOGT to other algorithms in predicting TF-CRE interactions using AUPR. Each point represents the prediction performance for an individual TF, with the x-axis showing AUPR values of other algorithm and the y-axis displaying SMOGT's AUPR.

**Alt Text:** The figure is a grouped box plot displaying AUPR values on the y axis across five different datasets. Each dataset compares the performance of the SMOGT against other methods. The visual data shows that SMOGT consistently exhibits higher median AUPR values compared to other methods across five datasets.

**Supplementary Figure S5.** BioStreamNet performance in threshold-dependent HRN configurations.

**Alt Text:** The figure consists of two panels. Panel A plots the PCC on the y axis against varying TF-CRE interaction thresholds on the x axis, visually demonstrating a flat trend with no significant fluctuation in accuracy. Panel B displays PCC across a range of CRE-CRE thresholds, similarly showing a stable distribution where performance metrics remain consistent despite increasing threshold percentages.

## Supplementary Note S1: Data preprocessing and Model Input

**1) *Data preprocessing***

To mitigate the effects of noise and data sparsity, we first performed data quality control, dimensionality reduction, and clustering following the Seurat pipeline (https://satijalab.org/seurat/). Quality control parameters were set as nFeature_RNA > 200 & percent.mt < 5. Dimensionality reduction used PCA and UMAP with default parameters, followed by cell clustering via FindNeighbors and FindClusters (resolution = 0.3-0.4). Since scATAC-seq is derived from the same cells as scRNA-seq, no QC was performed. Then, we used SEACells [1] to convert Seurat objects into metacells: for BM, A549, and GM12878 (7,000-10,000 filtered cells), metacell counts were set to 600-800 following SEACell recommendations, K562 and HCT116 (scCAT-seq) data omit this step. For unpaired scRNA-seq and scATAC-seq data, such as the Melanoma dataset used in this study, we utilized the LinearOT algorithm to perform modal alignment of the SEACells. scATAC-seq matrices (Genomic coordinates of all identified CREs are standardized to the GRCh38 reference genome using liftOver chain files) were integrated to generate multi-omics metacell matrices. Each metacell cluster ID was assigned by the most frequent cluster ID within it.

Highly variable genes (HVGs) and DEGs were identified using metacell matrices. FindAllMarkers in Seurat was used to detect cluster-specific DEGs and CREs, retaining 2,000 HVGs and top 300 DEGs per cluster, alongside 15,000 highly variable CREs (HVCREs) and top 3,000 differentially active CREs per cluster. BM cell annotation refers to [2], followed by Monocle3 (https://cole-trapnell-lab.github.io/monocle3/) and scVelo (https://scvelo.readthedocs.io/en/stable/) analyses with HSC as the root node.

We followed a multi-source data integration strategy to construct the initial hierarchical prior network. Specifically, for the TF-CRE and CRE-CRE layers, we downloaded high-quality TF ChIP-seq and Hi-C data (including intact, in situ, dilution, and capture Hi-C) from the ENCODE database, supplemented with data from ChIP-Atlas (specific IDs are listed in the Supplementary Table S2 and S3). Given the data redundancy in the 4D_project, we used it only as an auxiliary validation for the K562 cell line. For TF motif information, we obtained a comprehensive TF motif set using the JASPAR2024 R package with the collection parameter set to c("CORE", "CNE", "PHYLOFACTS", "SPLICE", "POLII", "FAM", "PBM", "PBM_HOMEO", "PBM_HLH", "UNVALIDATED"). For the TF-TF and TG-TG layers, we integrated three major protein-protein interaction databases, applying strict filtering criteria: we retained high-confidence links (combined_score > 600) from the STRING database [3], used version 4.4.231 of the BIOGRID database [4], and version 2 of the TRRUST database [5].

**2) *Model Input***

The feature matrices of TFs, TGs and CREs are represented, respectively, as:$\text{X}^{\text{TF}}\text{=}\left\{ \text{x}_{\text{ij}}^{\text{TF}}\text{|}\text{i}\text{=1,2,…,}\text{N}^{\text{TF}}\text{;}\text{j}\text{=1,2,…,}\text{M} \right\}$, $\text{X}^{\text{TG}}\text{=}\left\{ \text{x}_{\text{ij}}^{\text{TG}}\text{|}\text{i}\text{=1,2,…,}\text{N}^{\text{TG}}\text{;}\text{j}\text{=1,2,…,}\text{M} \right\}$, $\text{X}^{\text{CRE}}\text{=}\left\{ \text{x}_{\text{ij}}^{\text{CRE}}\text{|}\text{i}\text{=1,2,…,}\text{N}^{\text{CRE}}\text{;}\text{j}\text{=1,2,…,}\text{M} \right\}$, where $\text{N}^{\text{TF}}$, $\text{N}^{\text{TG}}$, $\text{N}^{\text{CRE}}$, $\text{M}$ refer to the quantities of TFs, TGs, CREs, and metacells, respectively.

Prior knowledge network: (1) TF-CRE regulatory pairs validated by ChIP-seq were obtained from JASPAR (core module), ChIP-Altas, and ENCODE; (2) CRE-CRE interactions validated by Hi-C were acquired from ENCODE and 4D-Nucleome; (3) Literature-documented protein-protein interactions (PPIs) and transcriptional regulatory relationships were collected from BIOGRID, TRRUST and STRING; (4) CRE-TG relationships were defined as CREs located within 2.5 Mbp upstream/downstream around transcription start sites (TSS). The resulting adjacency matrices for TF-TF, TF-CRE, CRE-CRE, and CRE-TG are represented, respectively, as: $\text{A}^{\text{TF-TF}}\text{∈}\text{R}^{\text{N}^{\text{TF}}\text{×}\text{N}^{\text{TF}}}$, $\text{A}^{\text{TF-CRE}}\text{∈}\text{R}^{\text{N}^{\text{TF}}\text{×}\text{N}^{\text{CRE}}}$, $\text{A}^{\text{CRE-CRE}}\text{∈}\text{R}^{\text{N}^{\text{CRE}}\text{×}\text{N}^{\text{CRE}}}$, $\text{A}^{\text{CRE-TG}}\text{∈}\text{R}^{\text{N}^{\text{CRE}}\text{×}\text{N}^{\text{TG}}}$.

## Supplementary Note S2: Some Concepts in Heterogeneous Graph and Detailed Description of HGT

**1) *Heterogeneous Graphs***

*A heterogeneous graph is defined as a directed graph* $\text{G}\text{=}\left( \text{V}\text{, }\text{E} \right)$ *associated with a node type mapping* $\text{φ}\left( \text{ν} \right)\text{:}\text{V}\text{→}\text{B}$ *and a edge type mapping* $\text{ψ}\left( \text{e} \right)\text{:}\text{E}\text{→}\text{Y}$*, where* $\nu\in V$ *and* $e\in E$ *denote the node set and edge set,* $\text{B}$ *and* $\text{Y}$ *are the node type set and edge type set, with the constrain of* $\left| \text{B} \right|\text{+}\left| \text{Y} \right|\text{>2}$. *There are three node types (TF, CRE and TG), and four edge types (TF-TF, TF-CRE, CRE-CRE and CRE-TG) in the HRN.*

**2) *Neighborhood set of target node***

*A neighborhood of a target node* $\text{v}_{\text{t}}$ *is a set of its source nodes edge and denoted as* $\text{N}\left( \text{v}_{\text{t}} \right)\text{=}\left\{ \text{v}_{\text{s}}\text{∈}\text{V}\text{:}\left( \text{v}_{\text{s}}\text{,}\text{v}_{\text{t}} \right)\text{∈}\text{E} \right\}$*, then the embedding update of the target node is achieved by using an encoder to aggregate information from the source node.*

**3) *Meta Path***

*For an edge* $e\in E\left( s,t \right)$*, where* $E\left( s,t \right)=\left\{ e\in E|v_{s}\in N(v_{t}) \right\}$*, its meta path is denoted as* $\left\langle\text{φ}\left( \text{v}_{\text{s}} \right)\text{,}\text{ψ}\left( \text{ }\text{e} \right)\text{, }\text{φ}\left( \text{v}_{\text{t}} \right) \right\rangle$*，and the embedding update of the target node is achieved by aggregating information from the source node along meta paths.*

**4) *Hierarchical Graph Transformer***

The process of HGT can be decomposed into three components, namely “Heterogeneous Mutual Attention”, “Heterogeneous Message Passing” and “Target-Specific Aggregation”.

*Heterogeneous mutual attention*

A multi-head mechanism is adopted to jointly attend to information from different embedding subspaces, and each head can run through an attention mechanism in parallel to reduce computational time. Here, we use the Heterogeneous Mutual Attention mechanism, where for given a target node $\text{v}_{\text{t}}$, and all its neighbors might belong to different distributions (TF, CRE and TG). Their mutual attention is calculated through their meta paths, i.e., the $\left\langle\text{φ}\left( \text{s} \right)\text{,}\text{ψ}\left( \text{ }\text{e} \right)\text{, }\text{φ}\left( \text{t} \right) \right\rangle$ triplets. According to the architecture design of Transformer, the target node $\text{v}_{\text{t}}$ and source node $\text{v}_{\text{s}}$ are mapped into Query, Key and Value vectors by linear projections respectively, and then the attention is calculated as dot product of these vectors. The $\text{i}$-th head in the $\text{l}$-th HGT layer is calculated as following:

$K_{s}^{i}={K-Linear}_{\varphi\left( s \right)}^{i}\left( H_{s}^{l-1} \right)$ (1)

$$Q_{t}^{i}={Q-Linear}_{\varphi\left( t \right)}^{i}\left( H_{t}^{l-1} \right)$$

$$V_{t}^{i}={V-Linear}_{\varphi\left( t \right)}^{i}\left( H_{t}^{l-1} \right)$$

$${ATT-head}_{\left( s,e,t \right)}^{i}=\left( K_{s}^{i}{{W_{\psi\left( e \right)}^{ATT}Q}_{t}^{i}}^{T} \right)\cdot\frac{\mu_{\left\langle\varphi\left( s \right),\psi\left( e \right), \varphi\left( t \right) \right\rangle}}{\sqrt{d}}$$

Where, $W_{\psi\left( e \right)}^{ATT}\in R^{\frac{d}{h}\times\frac{d}{h}}$ is a distinct edge-based matrix, which allows the model to capture different semantic relations even between the same node type pairs. $\mu\in R^{\left| A \right|\times\left| R \right|\times\left| A \right|}$ is a prior tensor to denote the general significance of each meta relation triplet, serving as an adaptive scaling to the attention. Then, Heterogeneous Mutual Attention is calculated as:

${Att-HGT}_{(s,e,t)}=_{{\forall v}_{s}\in N\left( t \right)}^{Softmax}\left( {}_{i\in\left[ 1,h \right]}^{||}{{ATT-head}_{\left( s,e,t \right)}^{i}} \right)$ (2)

*Heterogeneous message passing*

Heterogeneous information pass from source nodes to target nodes along the meta path by multi-head Message:

${MSG-head}_{\left( s,e,t \right)}^{i}={M-Linear}_{\varphi\left( s \right)}^{i}\left( H_{s}^{l-1} \right)W_{\psi\left( e \right)}^{MSG}$ (3)

$${MSG-HGT}_{(s,e,t)}={}_{i\in\left[ 1,h \right]}^{||}{{MSG-head}_{\left( s,e,t \right)}^{i}}$$

Where, $W_{\psi\left( e \right)}^{MSG}\in R^{\frac{d}{h}\times\frac{d}{h}}$ is a distinct edge-based matrix for incorporating the edge dependency.

*Target specific aggregation*

The attention vector is used as the weight to average the corresponding messages from the source nodes and the updated vector ${\tilde{\text{H}}}_{\text{t}}^{\text{l}}$ is obtained by aggregating information to the target node $\text{v}_{\text{t}}$ from all its neighbors (source nodes) of different feature distributions:

${\tilde{\text{H}}}_{\text{t}}^{\text{l}}\text{=}{}_{{\text{∀}\text{v}}_{\text{s}}\text{∈}\text{N}\left( \text{t} \right)}^{\text{⊕}}\left( {{\text{Att}\text{-}\text{HGT}}_{\left( \text{s}\text{,}\text{e}\text{,}\text{t} \right)}\text{∙}\text{MSG}\text{-}\text{HGT}}_{\left( \text{s}\text{,}\text{e}\text{,}\text{t} \right)} \right)$ (4)

Then the final embedding of $\text{v}_{\text{t}}$ is obtained by stacking information via all $\text{L}$ HGT layers ($\text{L}$ is set to be 2~4 in SMOGT for difference datasets through hyperparameters optimization):

${\text{H}_{\text{t}}^{\text{l}}\text{=}\text{θ}\left( \text{ReLU}\left( {\tilde{\text{H}}}_{\text{t}}^{\text{l}} \right) \right)\tilde{\text{H}}}_{\text{t}}^{\text{l}}\text{+}\left( \text{θ}\text{-1} \right)\text{H}_{\text{t}}^{\text{l}\text{-1}}$ (5)

where $\text{θ}$ is a trainable parameter and *ReLU* is the activation function.

## Supplementary Note S3: Loss Function and Training detail

During training, the BLCE employs a semi-supervised approach to select TF-CRE and CRE-CRE edges from the prior network with PCC (Pearson correlation coefficients) exceeding a specific threshold as pseudo-positive edges. To capture TF-specific regulatory patterns, pseudo-negative TF-CRE edges are generated through independent 1:1 random sampling of non-pseudo-positive and non-prior network pairs for each individual TF, with subsequent aggregation across all TF. For CRE-CRE interactions, reflecting their characteristic intra-chromosomal localization [6-8] , pseudo-negative edges are constructed via a dual sampling approach: (i) 1:1 random selection of qualified pairs within each chromosome, and (ii) additional 0.2-ratio sampling from inter-chromosomal pairs, followed by comprehensive integration of all chromosome-specific negative sets.

For MSE, TG embeddings are obtained via meta-paths, decod ed to match TG expression dimensions, and the squared error between predicted and original expression is minimized. The training strategy uses 5-fold cross-validation. Finally, the average of all node embeddings is taken as the final node embedding.

## Supplementary Note S4: The framework of BioStreamNet

The BioStreamNet module of SMOGT is developed for TG expression prediction. Inspired by the masking mechanism in scGO [9]. BioStreamNet constructs a sparse FCNN by an attention mask mechanism, which uses the mask matrix $\text{M}$ multiplied by the adjacency (or attention) matrix $\text{A}$ to set non-existing TF-to-TG connections to zero. The output of each layer is calculated as $H_{t}^{l}=\sigma\left[ \left( M*A \right)^{T}H_{s}^{l-1}+b \right]$, where $\text{b}$ is the bias vector, and $\text{* }$is the Hadamard product. Specifically: (1) Retain only CRE-TG links within 12 Mbp of each TG, CRE-CRE edges among these CREs and their neighbors, and all corresponding TF-CRE connections; (2) Use expression profiles of these CREs and TFs as inputs, propagating through non-zero connections to output predicted TG expression values.

## Supplementary Note S5: BioStreamNet-based Perturbation Modeling

To validate the causal effects of TFs and CREs on cell fate, we implemented an in silico perturbation module based on the trained, gene-specific BioStreamNet models. This module simulates the results of regulator knockout or overexpression by predicting genome-wide expression changes.

Specifically, for a given set of perturbations (e.g., gene1, gene2, CRE1...), the module first modifies the input expression matrix accordingly. For TF or CRE knockout simulations, the expression or accessibility value of the entity is set to zero across all metacells. Notably, for a CRE knockout, we also perform a structural perturbation: in any downstream gene model that includes the knocked-out CRE, all incoming and outgoing connection weights associated with that CRE are masked to zero during prediction. This dual-perturbation approach simulates both the loss of the CRE's activity and its removal from the HRNets. For TF or CRE overexpression, its expression/accessibility value in each metacell is multiplied by a specified factor. The modified expression/accessibility matrix is then fed into the pre-trained BioStreamNet models of the affected downstream TGs to predict their new expression levels, generating a post-perturbation transcriptome profile.

To visualize the causal impact of the perturbations, we adopted a framework inspired by SCENIC+ and scVelo. We calculate a delta embedding, which represents the predicted displacement of each cell in a low-dimensional space (such as UMAP or PCA). This displacement is then visualized as a vector field using a streamplot, thereby illustrating the direction and magnitude of the cell state transitions induced by the perturbation.

## Supplementary Note S6: Transition probabilities in MRWR_Pert

MRWR_Pert uses TFs as seed nodes, performs random walks on the HRN ending at TG nodes, and incorporates a TG-TG layer to simulate long-range interference and uncover potential perturbed relationships. Transition probabilities for TF-TF and TG-TG edges are determined by prior networks. Transition probabilities for CRE-TG are determined by prior networks. The transition probability for CRE-TG is set to 1 if the CRE lies within 12 Mbp of the TG; otherwise, it is set to 0. For TF-CRE and CRE-CRE edges, transition probabilities are set as: (1) Without cell specificity: the attention weight ${\text{Att}\text{-}\text{Wt}}_{\left( \text{i}\text{,}\text{j} \right)}$; (2) With cell specificity: ${\text{Att}\text{-}\text{Wt}}_{\left( \text{i}\text{,}\text{j} \right)}\text{×}\left( \left| \text{log}_{\text{2}}\text{FC}_{\text{i}} \right|\text{+}\left| \text{log}_{\text{2}}\text{FC}_{\text{j}} \right| \right)$. MRWR_Pert was implemented based on the HuMMuS Python library. The differential gene threshold was set as $\text{log}_{\text{2}}\text{FC>0.25}$ and $\text{p}\text{<0.01}$, and genes with scores greater than $\text{10}^{\text{-4}}$ were identified as perturbed.

The rationale for weighting transition probabilities by $\text{log}_{\text{2}}\text{FC}$ is rooted in Waddington's epigenetic theory and the post-Darwinian hard-wired network framework [10] , which posits that locally specific regulatory networks represent context-dependent activation or deactivation within global networks. Thus, highly expressed genes in the same cell subpopulation exhibit stronger regulatory relationships within the background network [11, 12], meaning that transition probabilities between regulatory nodes are higher.

## Supplementary Note S7: Evaluation metrics

We intersected the CREs identified by snATAC-seq with those recorded in the epigenetic databases, retaining only overlapping CREs. Based on ChIP-seq data from ENCODE and ChIP-Atlas for specific tissues/cells, TF-CRE edges with experimental evidence were labeled as positive, while those without were labeled as negative. We retained only TFs with >200 validated positive edges. Similarly, CRE-CRE interactions supported by HiC-seq data from ENCODE and 4D-project for specific tissues/cells were labeled as positive, while others were labeled as negative. Edge prediction performance was evaluated using AUPR, AUC, precision, recall, and Max F1 score (calculated by treating each TF-CRE pair's score as a threshold and selecting the maximum F1 value).

Perturb gene Enrichment：

$\text{Enrichment\_score\_preturb=}\left( \text{c}/\text{d} \right)/\left( \text{P}/\text{H} \right)$ (9)

where $\text{c}$ is the number of DEGs predicted as perturbed genes, $\text{d}$ is the total number of DEGs, $\text{P}$ is the total number of perturbed genes predicted from HVGs $\text{H}$ is the total number of HVGs. The biological meaning of enrichment score is that if a TF perturbation is biologically effective, the genes we predict to be affected should be significantly enriched within the set of genes that are actually differentially expressed. An enrichment score close to 1 implies that our prediction is no better than random chance (i.e., randomly selecting from highly variable genes). Therefore, a higher enrichment score indicates that our constructed network more accurately captures the true downstream gene expression changes induced by the TF perturbation.

SNP, eQTL and SE enrichment scores (consistent with eNet 2.0):

$\text{Enrichment\_score=}\left( \text{m}/\text{n} \right)/\left( \text{M}/\text{N} \right)$ (10)

where $\text{m}$ is the number of hub CREs containing disease loci, $\text{n}$ is the total number of hub CREs, $\text{M}$ is the number of HVCREs (preprocessed via snATAC) containing disease loci, $\text{N}$ is the total number of HVCREs. $\text{M}\text{/}\text{N}$ represents the background mutation rate.

## Supplementary Note S8: Parameter sensitivity analysis of PCC thresholds in SMOGT

To evaluate the learning performance of SMOGT under different PCC thresholds, we first analyzed the number of node types (Supplementary Table S4) and edge counts (Supplementary Table S5) between the prior network and the true network across 5 datasets (BM, K562, HCT116, A549 and GM12878) at varying thresholds. As the threshold increased, the proportion of true edges also rose, suggesting that the context-specific regulatory networks can be learned from omics profile.

Using the K562 dataset as an example, we selected 10 PCC thresholds (0.05, 0.1, 0.15, 0.2, 0.3, 0.4, 0.5, 0.6, 0.7 and 0.8) to test the model's performance, measuring the average TF-CRE AUPR and the PCC between predicted and actual TG values. SMOGT exhibited an upward trend with less variance in average TF-CRE AUPR at lower thresholds (0.05, 0.1, and 0.15; here, we focused on TF-CRE results, as CRE-CRE showed similar trends and were not shown in the manuscript), with the highest average AUPR observed at the 0.15 threshold. At higher correlation thresholds, the performance declined with large variance as the threshold increased (Supplementary Figure S1A).

These results indicate that at low thresholds, the model's performance declines due to confusion between the prior and true networks, whereas at high thresholds, the scarcity of semi-supervised edges leads to overfitting and eventual failure (evidenced by the stabilization and subsequent drop in average AUPR with increasing epochs). To achieve optimal learning performance, we selected the following dataset-specific TF-CRE and CRE-CRE thresholds based on their impact on the results across different datasets: BM (0.3–0.3), K562 (0.2–0.2), HCT116 (0.2–0.2), A549 (0.15–0.1), and GM12878 (0.1–0.1).

Notably, the PCC of TG predictions stabilized progressively with epochs across all thresholds (Supplementary Figure S1B). This observation suggests that TG expression prediction and edge prediction operate as decoupled tasks, enabling their joint learning within a unified framework.

## Supplementary Note S9: Architectural principles and parameter settings of the TF-CRE prediction algorithm benchmarked in our comparative analysis

**1) *regX***

regX [13] is the first algorithm to integrate cell fate regulator identification and cell lineage conversion within a unified graph neural networks (GNNs) framework in paired single-cell multi-omics data. Its core lies in the initial construction of a transcriptional activity matrix (TAM)—where TF-CRE weight learning is achieved via a 3-layer fully connected neural network (with TFs as the first layer, CREs as the second layer, and their summed output as the predicted TG value). Although regX incorporates GNNs (for modeling cell lineage conversion), its TAM learning relies solely on simplistic linear modeling, limiting its capability for mining deep regulatory relationships.

For fair comparison, we follow regX pipeline ([https://github.com/xixi-cathy/regX/](https://github.com/xixi-cathy/regX/)), using the SEACell metacell input data as SMOGT while retaining all TFs, TGs, and CREs. All other training parameters were set to default values.

**2) *LINGER***

LINGER [12], developed slightly earlier than regX, is the first deep learning model employing a continual learning framework to simultaneously predict both cis-regulatory patterns (CRE-TG) and trans-regulatory patterns (TF-TG). Its basic architecture consists of a 3-layer fully connected network: the first layer matches the number of TFs and CREs, the second contains 64 neurons, and the third corresponds to the number of TGs. LINGER implements two constraint mechanisms for TF-CRE modeling.

The first constraint utilizes a Laplacian matrix based on TF-CRE relationships identified through motif scanning:

$L_{L\text{a}place}(E_{TF},O,E_{i},\theta_{l})=tr((\theta_{l}^{(1)})^{T}L^{Norm}\theta_{l}^{(1)})$ (6)

$$\begin{matrix} \theta_{\text{l}}^{1}\in R^{(N_{TF}+N_{CRE})*64}\text{represents the node embedding matrix learned in the first layer，} \\ L^{N\text{or}m}\text{is the normalized Laplacian transform of the TF-CRE matrix identified } \\ \text{through motif scanning } \end{matrix}$$

The core principle of the first constraint is to leverage prior TF-CRE relationships to regularize the learned node embeddings

The second approach employs the Elastic Weight Consolidation (EWC) algorithm from continual learning. This method transfers pre-trained parameters from BulkNN (a model pre-trained on ENCODE bulk epigenomic sequencing data) to single-cell data for fine-tuning. By leveraging the Fisher Information Matrix, EWC ensures that the model maintains "respect" for the old parameters of BulkNN during adaptation, thereby preventing catastrophic forgetting and enhancing model stability and interpretability.

$\begin{matrix} L_{EWC}(E_{TF},O,E_{i},\theta_{l})=\frac{1}{(N_{TF}+N_{CRE})*64}\sum_{i=1}^{N_{TF}+N_{CRE}} \sum_{i=1}^{64} F_{ij}(\theta_{l}^{(1)})_{i,j}(\theta_{b}^{(1)})_{i,j} \\ F_{ij}=E[(\frac{\partial}{\partial(\theta_{b}^{(1)})_{i,j}})L_{MSE}(\mathop{E}\limits^{\sim} _{TF},\mathop{O}\limits^{\sim} ,\mathop{E}\limits^{\sim} _{i},\theta_{b})^{2}] \end{matrix}$ (7)

$$\begin{matrix} \theta_{b}^{(1)}\text{represents the node embeddings of the first layer in BulkNN}; \\ L_{MSE}(\mathop{E}\limits^{\sim} _{TF},\mathop{O}\limits^{\sim} ,\mathop{E}\limits^{\sim} _{i},\theta_{b})^{2}\text{represents BulkNN's MSE loss.} \end{matrix}$$

Furthermore, LINGER employs Shapley values to quantify both cis-regulatory strength (CRE-TG) and trans-regulatory strength (TF-TG) in the deep learning network.

While the aforementioned dual constraints endow LINGER's inferred TF-CRE relationships with high interpretability, the model suffers from limited learning capacity and poor generalization performance on novel datasets due to excessively rigid constraints and an oversimplified architecture (only 3 layers).

For fair comparison, we follow LINGER protocol ([https://github.com/Durenlab/LINGER](https://github.com/Durenlab/LINGER)) with default parameters, except for replacing pseudo-bulk data with SEACell-generated metacells to maintain consistency with our pipeline

**3) *TRIPOD***

TRIPOD [14] employs a control-matching strategy to establish conditional associations (Level 1) or interaction models (Level 2) for constructing TF-CRE-TG tripartite regulatory relationships. The specific workflow is as follows:

(1) Cell Pair Matching, for a fixed TF (or CRE), identify cell pairs with the most similar TF (or CRE) expression levels:

$Y_{\text{t}i}\approx Y_{\text{t}j}$ (8)

(2) Differential Expression Calculation, Compute the differential expression values for CRE and TG across matched cell pairs;

$\begin{matrix} \Delta X_{C}=X_{C\text{i}}-X_{C\text{j}} \\ \Delta Y_{g}=X_{g\text{i}}-X_{g\text{j}} \end{matrix}$ (9)

(3) For Level 1, Spearman correlation analysis are performed between$\Delta X_{C}\text{ and }\Delta X_{\text{g}}$ ;

(4) For Level 2, fit an interaction model:

$\Delta Y_{\text{g}}=\alpha\Delta X_{C}+\gamma(\mathop{Y}\limits^{-} _{\text{t}}*\Delta X_{C})+\varepsilon$ (10)

$$\begin{matrix} \text{where}: \\ \mathop{Y}\limits^{-} _{\text{t}}\text{is the mean TF expression in the matched cell pairs}; \\ \text{The interaction term coefficient }\gamma\text{ qantifies the TF-CRE regulatory strength } \\ \text{under the given TG condition}. \end{matrix}$$

In this study, we follow the TRIPOD pipeline ([https://github.com/yuchaojiang/TRIPOD](https://github.com/yuchaojiang/TRIPOD)), with the following 2 modifications:

(1) Replaced Seurat WNN algorithm with SEACell-generated metacells;

(2) Adopted the TF-expression-controlled matching strategy and Level 2 interaction modeling to derive TF-CRE scores.

**4) *REUNION***

REUNION [15] is the first method to employ a pseudo-label semi-supervised learning strategy for predicting TF-CRE binding in paired single-cell multi-omics data. The negative sampling approach in our study is derived from this work. REUNION consists of a two-stage training process:

(1) Complementary Scoring Module (Unify) for TF-CRE Scoring, this module evaluates the association between TFs and CRE-TG pairs using the following scoring function

 (11)

(2) Semi-Supervised Learning Framework (Rediscover), based on the TF-CRE labels identified by Unify, Rediscover performs the following steps:

(a) Singular Value Decomposition (SVD) is applied to both the motif scan-derived CRE-TF matrix and the CRE expression matrix. The top NN singular vectors from each are extracted and concatenated to generate the CRE embeddings;

(b) CRE embeddings are clustered using PhenoGraph or k-means, with cluster labels assigned;

(c) Positive samples were selected from high TF-CRE scores in Unify with motif scan confirmation (motif scan = 1), while negative samples were sampled based on motif presence/absence and cluster labels;

(d) A logistic regression model is trained for the final TF-CRE interaction prediction.

In our study, we follow the REUNION pipeline ([https://github.com/yangymargaret/REUNION](https://github.com/yangymargaret/REUNION)) with modifications: the Unify module was replaced with TF-CRE Spearman correlation analysis using SEACell-derived metacells, motif scanning was performed using MEME (threshold p-value < 1×10⁴), the top 100 SVD components were retained, k-means clustering was applied to CRE embeddings, and logistic regression was used as the classifier, with other parameters kept at default settings.

**5) *STREAM***

STREAM [16] is the first method to model TF, CRE, and TG as leaf nodes in a Steiner Forest Problem Model, enabling the identification of tree-shaped regulatory networks (topological clusters of CREs). The framework operates through four stages:

(1) Functional Gene Module Prediction. The QUBIC biclustering algorithm is employed to simultaneously identify both gene and cell modules;

(2) Steiner Forest Optimization, the minimum-cost forest is identified within TF-CRE networks constructed by Signac and Cicero:

$F^{(k)}=\bigcup_{s=1}^{t} T_{s}^{(k)}$ (12)

$$\text{where},\text{ }T_{s}^{(k)}\text{ is defined as subtrees connecting all terminal nodes}$$

(3) Hybrid biclustering is performed through vertical expansion, where co-expressed CREs or TF/TGs are incorporated, followed by horizontal expansion in which cells matching expression patterns are added to refine CRE-TG/TF relationships;

(4) Submodular Optimization, Redundant interactions are eliminated to form enhancer regulons (eRegulons: TF-CRE-TG triplets).

we follow STREAM protocol ([https://github.com/OSU-BMBL/STREAM](https://github.com/OSU-BMBL/STREAM)), with SEACell-derived metacell used as input data, JASPAR core PWM obtained from STREAM's repository for motif analysis, and all other parameters maintained at default configurations.

**6) *Pando***

Pando [17] employs multivariate regression modeling with interaction terms to characterize TF-CRE relationships. In this study, we follow the Pando protocol ([https://github.com/quadbio/Pando](https://github.com/quadbio/Pando)), with SEACell-derived metacell used as input data and all other parameters maintained at default configurations.

**7) *Configuration of TG-associated CREs***

In regX and LINGER, CREs surrounding TGs/TFs were defined as genomic regions within ±100 kb and ±1 kb of transcription start sites (TSS), respectively, as fixed parameters inherent to their frameworks. All other algorithms, including SMOGT, uniformly adopted a ±25 kb window around TSS to designate TG/TF-associated CREs.

## Supplementary Note S10: Architectural principles and parameter settings of the CRE-CRE prediction algorithm benchmarked in our comparative analysis

For comparative analysis, we selected two established scATAC-seq analytical frameworks - Cicero and ArchR - as benchmark methods. Following their standard pipelines, we used ATAC-seq raw counts as input with the default 50KB genomic window size.

## Supplementary Note S11: Sensitivity analysis of network weight constraints in BioStreamNet for target gene expression prediction

For TF-CRE, the threshold was determined by the dot product of TF and CRE embeddings. To remove chromosome size effects on CRE-CRE, we employed a chromosome-specific normalization approach wherein edge thresholds were defined as the top N CRE embedding dot products per chromosome. Model performance was quantified using PCC between predicted and TG expression.

When the CRE-CRE threshold was fixed at 2%, TF-CRE thresholds were systematically evaluated at values of 0.75, 0.80, 0.85, and 0.90. No significant performance variation was observed across the tested threshold values (Supplementary Figure S4A).

When the TF-CRE threshold was fixed at 0.8, CRE-CRE thresholds were systematically examined at 2%, 3%, 4%, 5%, 6%, 8%, and 10%. Only minimal performance enhancement was detected with increasing threshold values, with no statistically significant differences observed (Supplementary Figure S4B).

These findings collectively indicate that optimal learning in BioStreamNet can be achieved through a limited number of core network parameters.

## Supplementary Note S12: Architectural principles and parameter settings of the TG expression prediction algorithm benchmarked in our comparative analysis

SCARlink employs regularized Poisson regression to predict TG expression values using CREs within ±250 kb of TSS

$\text{loss}=\frac{1}{N}\sum_{i=1}^{N} (e^{X_{i}w+\varepsilon}-Y_{i}(X_{i}w+\varepsilon))+\alpha||w||_{2}^{2}$ (13)

$$\text{where}:\text{w represents the regression coefficient}$$

To ensure comparability across methods, we replaced SCARlink's original ArchR normalization pipeline with SAECell metacells (https://github.com/snehamitra/SCARlink), while keeping all other parameters consistent.

The definition of regX is provided in Supplementary Note S3.

## Supplementary Note S13: Architectural principles and parameter settings of the Driver regulator identification algorithm benchmarked in our comparative analysis

**1) *ScenicPlus***

ScenicPlus [18] is a single-cell multi-omics regulatory network modeling framework for enhancer-driven gene regulatory networks (eGRNs) that identifies enhancer regulons (eRegulons). The core workflow consists of four key analytical steps:

(1) PycisTopic-based CRE module identification;

(2) Cell cluster-specific differential features were detected in find_diff_features module;

(3) PycisTarget was employed to identify transcription factors (TFs) whose binding motifs are significantly enriched in the identified CRE modules;

(4) Regression modeling of CRE-TG and TF-TG interactions;

(5) AUCell Enrichment analysis for eRegulon characterization.

In this study, we implemented the ScenicPlus analytical pipeline (https://scenicplus.readthedocs.io/en/latest/) using BM datasets processed through Cell Ranger ARC. Primary input files included the ATAC-seq fragment file (atac_fragments.tsv.gz) and filtered feature-barcode matrix (filtered_feature_bc_matrix.h5). The find_diff_features function was configured with a log2 fold-change threshold (log2FC_thr) of 0.5, while all other parameters remained at their default settings.

**2) *CEFCON***

CEFCON [19] is a GNN framework designed for identifying cell fate Driver Regulator from scRNA-seq data, comprising two core modules:

(1) TG/TF expression profiles were projected into a low-dimensional space through two-layer graph attention networks (GATs), with a graph contrastive learning strategy being employed

 (14)

$$\text{ where: }\mathop{\text{X}}\limits^{\text{\textasciitilde}} \text{ }and\mathop{A}\limits^{\text{\textasciitilde}} \text{ represent shuffled node features and perturbed graph data X, respectively }$$

(2) Driver genes are identified by the MDS and MFVS algorithms, with the driver score being defined as

$\lambda(\ln(1+\sum_{j\in N_{j}^{p}} \beta_{ji}^{out}))+(1-\lambda)(\ln(1+\sum_{i\in N_{j}^{s}} \beta_{ij}^{in}))$ (15)

$$\text{where}: \beta represents attention score$$

We follow the CEFCON pipeline (https://github.com/WPZgithub/CEFCON). All parameters are maintained at their default settings, and the prior network is obtained from the CEFCON repository (https://github.com/WPZgithub/CEFCON/blob/main/prior_data/network_human.zip).

## Supplementary Note S14: Biomedical Evidence for Driver Genes in Bone Marrow Stem Cell Lineages

1 HSC

|  | ***PBX1*** | ***FOXO1*** | ***THRB*** | ***MECOM*** | ***L3MBTL4*** |
| --- | --- | --- | --- | --- | --- |
| Protein Family | TALE Homeodomain Protein | Forkhead Box O | Nuclear Hormone Receptor | Zinc Finger/SET Domain Protein | Polycomb (MBT Domain |
| Core Physiological Function in HSCs | Maintains Quiescence/Self-renewal | Antioxidative Stress | Regulates Erythroid Differentiation [20] | Drives Self-renewal/Inhibits Differentiation [21] | Chromatin Silencing |
| Primary Role in Hematologic Malignancies | Oncogene (Fusion Protein) | Context-dependent (Oncogene/Tumor Suppressor) | Tumor Suppressor | Oncogene | Tumor Suppressor |
| Key Molecular Alterations | t(1;19) E2A-PBX1 Fusion [22, 23] | Activating Point Mutations/Expression Dysregulation | v-erbA Model/Expression Silencing | inv(3)/t(3;3) MECOM Rearrangement [24] | del(20q) |
| Major Associated Diseases | B-ALL | DLBCL/AML/BCP-ALL | Erythroleukemia/AEL | AML/MDS | MDS/AML |
| Clinical Biomarker Value | MRD Target/Prognosis [25] | Prognostic/Resistance Marker [26] | Differentiation Status Marker | High-Risk Prognostic Marker [24, 27] | Prognosis (Dependent on Co-occurring Aberrations) |
| Primary Targeting Strategies |  | AKT/FOXO1 Inhibitors [28, 29] | TRβ Agonists/Epigenetic Drugs [30] | BET Inhibitors/Targeting EVI1 Pathway [31] | Synthetic Lethality Targets |

2 Ery

|  | ***GATA2*** | ***GATA1*** | ***MYC*** | ***KLF1*** |
| --- | --- | --- | --- | --- |
| Protein Family | GATA zinc finger protein | GATA zinc finger protein | Basic helix-loop-helix transcription factor | Krüppel-like zinc finger protein |
| Core Physiological Function in HSCs | Maintains HSC self-renewal and survival | Drives erythroid/megakaryocytic differentiation | Drives cell proliferation and metabolism | Regulates terminal erythroid differentiation and globin switching |
| Primary Role in Hematologic Malignancies | Tumor Suppressor | Context-dependent (Oncogene/Tumor Suppressor) | Oncogene |  |
| Key Molecular Alterations | Germline mutation (haploinsufficiency, gene at 3q21.3), often with monosomy 7 | Germline/somatic mutation (loss/change of function, gene at Xp11.23) | Gene rearrangement/amplification (e.g., translocation with Ig locus [14q32, etc.], gene at 8q24) | Germline mutation (haploinsufficiency/dominant-negative, gene at 19p13.2) |
| Major Associated Diseases | GATA2 deficiency syndrome (MDS/AML) | ML-DS, DBA, Congenital Dyserythropoietic Anemia (CDA) | High-risk leukemia/lymphoma | CDA type IV, HPFH, β-thalassemia |
| Clinical Biomarker Value | Allogeneic hematopoietic stem cell transplantation (allo-HSCT) [32, 33] | Diagnostic and MRD monitoring marker for ML-DS; pathomechanism marker for DBA [34] | High-risk prognostic marker [35, 36] | Diagnostic marker for CDA type IV; phenotypic modifier for hemoglobinopathies [37][<https://www.orpha.net/en/disease/detail/293825> ] |
| Primary Targeting Strategies | Allogeneic hematopoietic stem cell transplantation (allo-HSCT) [30, 38] | "Universal" GATA1 gene therapy (DBA) [39][https://answers.childrenshospital.org/universal-gene-therapy-diamond-blackfan-anemia/ | BET inhibitors, PROTACs [40, 41] | Targeting BCL11A/KLF1 axis, gene editing to induce HbF [42, 43] |

3 Myeloid

|  | ***IRF8*** | ***CEBPD*** | ***RFX8*** | ***KLF4*** |
| --- | --- | --- | --- | --- |
| Protein Family | Interferon Regulatory Factor 8 | CCAAT/Enhancer Binding Protein Delta | Regulatory Factor X8 | Krüppel-like Factor 4 |
| Core Physiological Function | Dose-dependent regulation of monocyte and dendritic cell lineage specification | Inducible transcription factor in macrophage inflammatory responses |  | Acts as a downstream effector of PU.1 to drive monocyte differentiation |
| Primary Role in Hematologic Malignancies | Context-dependent: Tumor suppressor (in CML/some AML) or oncogene dependency factor (in some AML subtypes) | Tumor suppressor | Oncogene | Context/Hierarchy-dependent: Tumor suppressor (in bulk AML cells) or oncogene dependency factor (in leukemia stem cells) |
| Key Molecular Alterations | Downregulation/loss of expression, or upregulation via super-enhancer | Epigenetic silencing via promoter hypermethylation | Significant transcriptional upregulation; may be associated with t(2;2)(p11;q11) chromatin rearrangement in some cases (gene at 2q11.2) | Downregulation, or upregulation in MLL-rearranged AML |
| Major Associated Diseases | AML (subtype-specific), CML, Myeloid Sarcoma, BPDCN | AML | AML | AML (especially MLL-rearranged AML) |
| Clinical Biomarker Value | Diagnostic: MS, BPDCN; Prognostic: High expression correlates with poor RFS; Predictive: May predict response to ATRA/LSD1i [44] | Prognostic: Low expression correlates with poor prognosis; can predict HMA efficacy [45] | Prognostic: High expression correlates with poor prognosis [46] | Prognostic (subtype-dependent): High expression is a feature of high-risk MLL-rearranged AML [47] |
| Primary Targeting Strategies | Inhibition of IRF8/downstream pathways (for high-expression AML) or epigenetic reactivation (for low-expression AML) [48] | Reactivation of its expression via hypomethylating agents (e.g., azacitidine) [45] | Inhibition. Chelerythrine is a potential drug, providing leads for new drug development [46] | Targeting LSC maintenance function. Eradicate leukemia stem cells by modulating KLF4 activity |

4 CLP

|  | ***EBF1*** | ***PAX5*** | ***FOXO1*** | ***AFF3*** |
| --- | --- | --- | --- | --- |
| Protein Family | COE (Collier/Olf-1/EBF) | Paired Box (PAX) | Forkhead Box O (FOXO) | AF4/FMR2 (ALF) |
| Core Physiological Function | B-cell lineage commitment and differentiation | B-cell identity maintenance and lineage locking | Cellular stress response, metabolism, and survival | Involved in transcriptional elongation |
| Primary Role in Hematologic Malignancies | Tumor suppressor | Tumor suppressor | Context-dependent: Tumor suppressor or oncogene | Oncogene (only when fused with KMT2A) |
| Key Molecular Alterations | Gene deletion/inactivating mutation (tumor suppressor) (5q34); Gene fusion (oncogene) (e.g., t(5;5)(q33;q31-32)) | Gene deletion, inactivating mutation, fusion (impaired function) (9p13) | Activating point mutation (oncogene) (13q14.11); Expression downregulation (tumor suppressor) | Gene fusion (oncogene) (t(2;11)(q11.2;q23)) |
| Major Associated Diseases | B-ALL (especially relapsed/high-risk) | B-ALL | DLBCL, Burkitt lymphoma, BCP-ALL, AML | KMT2A (MLL)-rearranged B-ALL |
| Clinical Biomarker Value | Poor prognosis (deletion) ; Therapeutic target (fusion) [49, 50] | Subtype classification ; Prognosis (depends on specific alteration) [51] | Independent poor prognosis (DLBCL mutation) ; Drug resistance marker [26] | High-risk prognosis ; Diagnostic marker [52] |
| Primary Targeting Strategies | Targeting EBF1-PDGFRB fusion kinase (TKI) [53] | Targeting downstream pathways of fusion proteins (e.g., LCK, JAK) [54] | AKT/FOXO1 inhibitors [55] | Targeting MLL fusion complex (Menin, DOT1L inhibitors) |

**References**

1. Persad S, Choo ZN, Dien C et al. SEACells infers transcriptional and epigenomic cellular states from single-cell genomics data. Nat Biotechnol 2023;41:1746-1757.

2. Setty M, Kiseliovas V, Levine J et al. Characterization of cell fate probabilities in single-cell data with Palantir. Nat Biotechnol 2019;37:451-460.

3. Szklarczyk D, Kirsch R, Koutrouli M et al. The STRING database in 2023: protein-protein association networks and functional enrichment analyses for any sequenced genome of interest. Nucleic Acids Res 2023;51:D638-D646.

4. Oughtred R, Rust J, Chang C et al. The BioGRID database: A comprehensive biomedical resource of curated protein, genetic, and chemical interactions. Protein Sci 2021;30:187-200.

5. Han H, Cho JW, Lee S et al. TRRUST v2: an expanded reference database of human and mouse transcriptional regulatory interactions. Nucleic Acids Res 2018;46:D380-D386.

6. Chen Z, Snetkova V, Bower G et al. Increased enhancer-promoter interactions during developmental enhancer activation in mammals. Nat Genet 2024;56:675-685.

7. Oberbeckmann E, Quililan K, Cramer P et al. In vitro reconstitution of chromatin domains shows a role for nucleosome positioning in 3D genome organization. Nat Genet 2024;56:483-492.

8. Pollex T, Rabinowitz A, Gambetta MC et al. Enhancer-promoter interactions become more instructive in the transition from cell-fate specification to tissue differentiation. Nat Genet 2024;56:686-696.

9. Wu Y, Xu P, Wang L et al. scGO: interpretable deep neural network for cell status annotation and disease diagnosis. Brief Bioinform 2024;26.

10. Huang S. The molecular and mathematical basis of Waddington's epigenetic landscape: a framework for post-Darwinian biology? Bioessays 2012;34:149-157.

11. Elliott K, Larsson E. Non-coding driver mutations in human cancer. Nat Rev Cancer 2021;21:500-509.

12. Yuan Q, Duren Z. Inferring gene regulatory networks from single-cell multiome data using atlas-scale external data. Nat Biotechnol 2025;43:247-257.

13. Xi X, Li J, Jia J et al. A mechanism-informed deep neural network enables prioritization of regulators that drive cell state transitions. Nat Commun 2025;16:1284.

14. Jiang Y, Harigaya Y, Zhang Z et al. Nonparametric single-cell multiomic characterization of trio relationships between transcription factors, target genes, and cis-regulatory regions. Cell Syst 2022;13:737-751.e734.

15. Yang Y, Pe'er D. REUNION: transcription factor binding prediction and regulatory association inference from single-cell multi-omics data. Bioinformatics 2024;40:i567-i575.

16. Li Y, Ma A, Wang Y et al. Enhancer-driven gene regulatory networks inference from single-cell RNA-seq and ATAC-seq data. Brief Bioinform 2024;25.

17. Fleck JS, Jansen SMJ, Wollny D et al. Inferring and perturbing cell fate regulomes in human brain organoids. Nature 2023;621:365-372.

18. Bravo González-Blas C, De Winter S, Hulselmans G et al. SCENIC+: single-cell multiomic inference of enhancers and gene regulatory networks. Nat Methods 2023;20:1355-1367.

19. Wang P, Wen X, Li H et al. Deciphering driver regulators of cell fate decisions from single-cell transcriptomics data with CEFCON. Nat Commun 2023;14:8459.

20. Gao X, Lee HY, Li W et al. Thyroid hormone receptor beta and NCOA4 regulate terminal erythrocyte differentiation. Proc Natl Acad Sci U S A 2017;114:10107-10112.

21. Kataoka K, Sato T, Yoshimi A et al. Evi1 is essential for hematopoietic stem cell self-renewal, and its expression marks hematopoietic cells with long-term multilineage repopulating activity. J Exp Med 2011;208:2403-2416.

22. Kager L, Lion T, Attarbaschi A et al. Treatment Response and Outcome in Childhood t(1;19)/TCF3-PBX1 Positive Acute Lymphoblastic Leukemia: A Report from the Austrian BFM Group. Blood 2005;106:1458-1458.

23. Liu M, Xing Y, Tan J et al. Comprehensive summary: the role of PBX1 in development and cancers. Front Cell Dev Biol 2024;12:1442052.

24. Moscvin M, Schwede M, Mannis G et al. Acute Myeloid Leukemia with Inv(3) or t(3;3): A Clinical and Cytogenetic Characterization of 40 Patients. Blood 2023;142:4231-4231.

25. Hu Y, He H, Lu J et al. E2A-PBX1 exhibited a promising prognosis in pediatric acute lymphoblastic leukemia treated with the CCLG-ALL2008 protocol. Onco Targets Ther 2016;9:7219-7225.

26. Trinh DL, Scott DW, Morin RD et al. Analysis of FOXO1 mutations in diffuse large B-cell lymphoma. Blood 2013;121:3666-3674.

27. Ottema S, Mulet-Lazaro R, Beverloo HB et al. Atypical 3q26/MECOM rearrangements genocopy inv(3)/t(3;3) in acute myeloid leukemia. Blood 2020;136:224-234.

28. Lin S, Ptasinska A, Chen X et al. A FOXO1-induced oncogenic network defines the AML1-ETO preleukemic program. Blood 2017;130:1213-1222.

29. Wang F, Demir S, Gehringer F et al. Tight regulation of FOXO1 is essential for maintenance of B-cell precursor acute lymphoblastic leukemia. Blood 2018;131:2929-2942.

30. Zhu X, Zhao L, Doolittle WKL et al. Reactivated thyroid hormone receptor beta attenuates anaplastic thyroid cancer (ATC) stem cell activity. Endocr Relat Cancer 2023;30.

31. Birdwell CE, Fiskus W, Kadia TM et al. Preclinical efficacy of targeting epigenetic mechanisms in AML with 3q26 lesions and EVI1 overexpression. Leukemia 2024;38:545-556.

32. Kotmayer L, Romero-Moya D, Marin-Bejar O et al. GATA2 deficiency and MDS/AML: Experimental strategies for disease modelling and future therapeutic prospects. Br J Haematol 2022;199:482-495.

33. Wlodarski MW, Hirabayashi S, Pastor V et al. Prevalence, clinical characteristics, and prognosis of GATA2-related myelodysplastic syndromes in children and adolescents. Blood 2016;127:1387-1397; quiz 1518.

34. Queiroz LB, Lima BD, Mazzeu JF et al. Analysis of GATA1 mutations and leukemogenesis in newborns with Down syndrome. Genet Mol Res 2013;12:4630-4638.

35. Ohanian M, Rozovski U, Kanagal-Shamanna R et al. MYC protein expression is an important prognostic factor in acute myeloid leukemia. Leuk Lymphoma 2019;60:37-48.

36. Rodrigues JM, Hollander P, Schmidt L et al. MYC protein is a high-risk factor in mantle cell lymphoma and identifies cases beyond morphology, proliferation and TP53/p53 - a Nordic Lymphoma Group study. Haematologica 2024;109:1171-1183.

37. Catapano R, Sessa R, Trombetti S et al. Identification and Functional Analysis of Known and New Mutations in the Transcription Factor KLF1 Linked with beta-Thalassemia-like Phenotypes. Biology (Basel) 2023;12.

38. Hickstein D. HSCT for GATA2 deficiency across the pond. Blood 2018;131:1272-1274.

39. Voit RA, Liao X, Caulier A et al. Regulated GATA1 expression as a universal gene therapy for Diamond-Blackfan anemia. Cell Stem Cell 2025;32:38-52 e36.

40. Abedin SM, Boddy CS, Munshi HG. BET inhibitors in the treatment of hematologic malignancies: current insights and future prospects. Onco Targets Ther 2016;9:5943-5953.

41. Li Z, Lim SL, Tao Y et al. PROTAC Bromodomain Inhibitor ARV-825 Displays Anti-Tumor Activity in Neuroblastoma by Repressing Expression of MYCN or c-Myc. Front Oncol 2020;10:574525.

42. Peralta R, Low A, Kim A et al. Targeting BCL11A and KLF1 For The Treatment Of Sickle Cell Disease and β-Thalassemia In Vitro using Antisense Oligonucleotides. Blood 2013;122:1022-1022.

43. Wienert B, Martyn GE, Kurita R et al. KLF1 drives the expression of fetal hemoglobin in British HPFH. Blood 2017;130:803-807.

44. Hartung EE, Singh K, Coopersmith S et al. The Transcription Factor IRF8 Regulates the Sensitivity of AML Cells to LSD1 Inhibition and All-Trans Retinoic Acid. Blood 2024;144:2764-2764.

45. Prajapati SC, Meydan C, Neelamraju Y et al. CCAAT-enhancer binding protein delta functions as a tumor suppressor gene in acute myeloid leukemia. bioRxiv 2025.

46. Cui Z, Fu Y, Zhou M et al. Pan-cancer investigation of RFX family and associated genes identifies RFX8 as a therapeutic target in leukemia. Heliyon 2024;10:e35368.

47. Yadav SS, Kalia P, Kaur N et al. KLF4 in cancer chemoresistance: molecular mechanisms and therapeutic implications. Discov Oncol 2025;16:1690.

48. Cao Z, Budinich KA, Huang H et al. ZMYND8-regulated IRF8 transcription axis is an acute myeloid leukemia dependency. Mol Cell 2021;81:3604-3622 e3610.

49. Ramirez-Komo JA, Delaney MA, Straign D et al. Spontaneous loss of B lineage transcription factors leads to pre-B leukemia in Ebf1(+/-)Bcl-x(L)(Tg) mice. Oncogenesis 2017;6:e355.

50. Schwab C, Ryan SL, Chilton L et al. EBF1-PDGFRB fusion in pediatric B-cell precursor acute lymphoblastic leukemia (BCP-ALL): genetic profile and clinical implications. Blood 2016;127:2214-2218.

51. Ratti S, Lonetti A, Follo MY et al. B-ALL Complexity: Is Targeted Therapy Still A Valuable Approach for Pediatric Patients? Cancers (Basel) 2020;12.

52. Stutterheim J, Van der Sluis IM, Vrenken KS et al. KMT2A-rearranged acute lymphoblastic leukemia in infants: current progress and challenges. Haematologica 2025;110:1951-1961.

53. Weston BW, Hayden MA, Roberts KG et al. Tyrosine kinase inhibitor therapy induces remission in a patient with refractory EBF1-PDGFRB-positive acute lymphoblastic leukemia. J Clin Oncol 2013;31:e413-416.

54. Schinnerl D, Fortschegger K, Kauer M et al. The role of the Janus-faced transcription factor PAX5-JAK2 in acute lymphoblastic leukemia. Blood 2015;125:1282-1291.

55. Gehringer F, Weissinger SE, Swier LJ et al. FOXO1 Confers Maintenance of the Dark Zone Proliferation and Survival Program and Can Be Pharmacologically Targeted in Burkitt Lymphoma. Cancers (Basel) 2019;11.
