# Supplementary figures and images for: Deciphering hierarchical regulatory network of cell fate via an epigenetics-informed heterogeneous graph transformer on single-cell multi-omics data
Source: Brief Bioinform. 2025 Dec 12;26(6):bbaf664. doi: 10.1093/bib/bbaf664 (PMC12875533; doi:10.1093/bib/bbaf664)

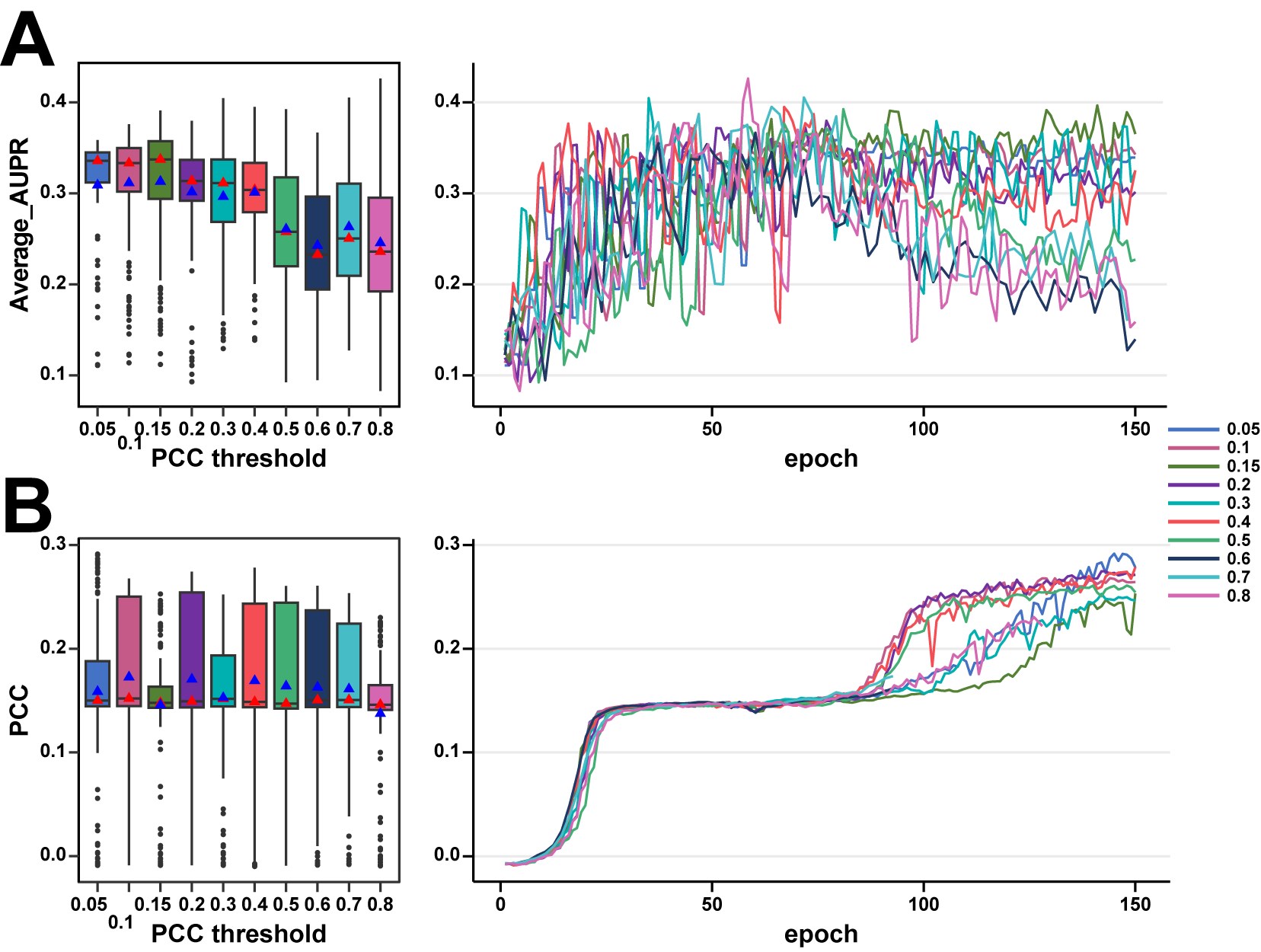

Supplement: Supplementary_Figure_S1_bbaf664 [file supplementary_figure_s1_bbaf664.jpeg]

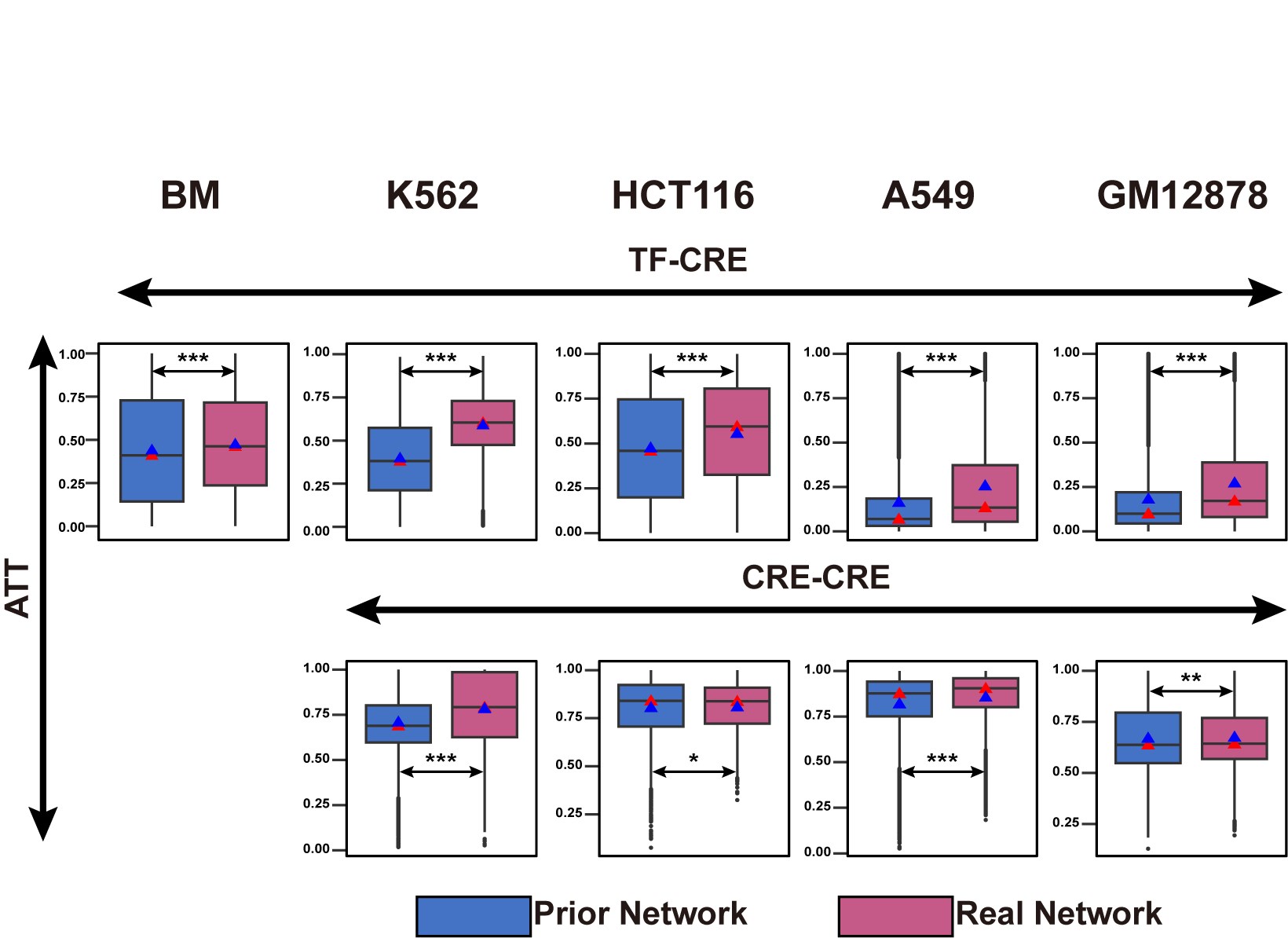

Supplement: Supplementary_Figure_S2_bbaf664 [file supplementary_figure_s2_bbaf664.jpeg]

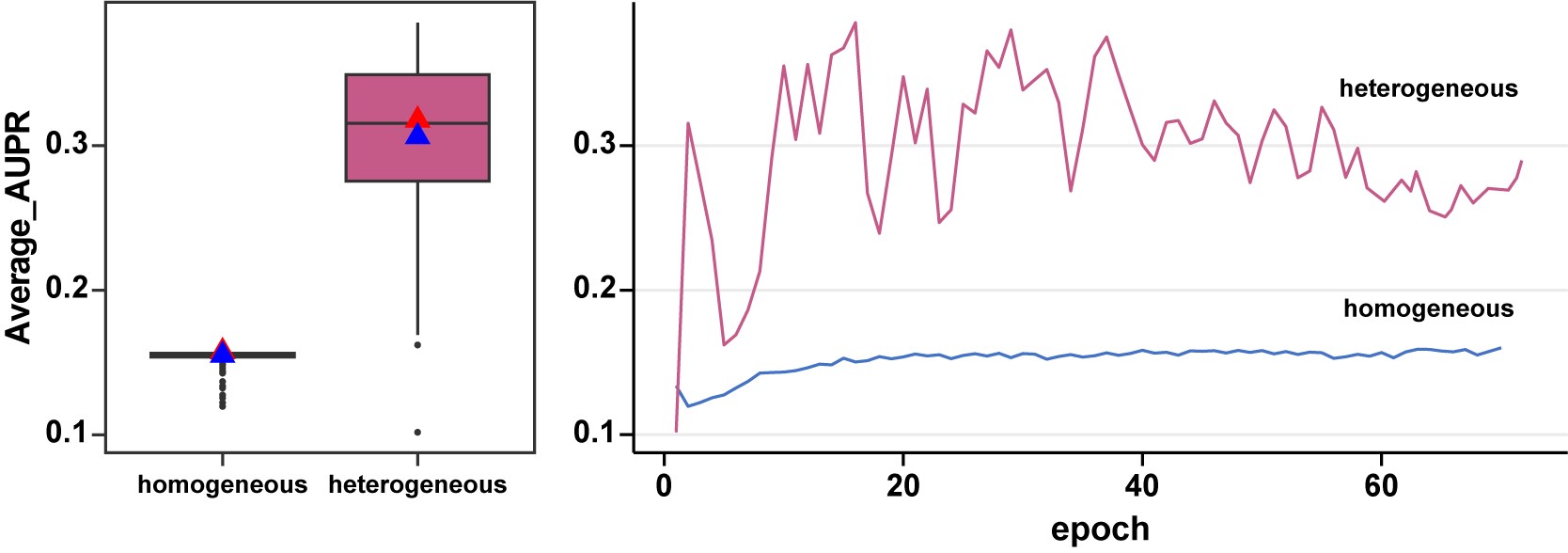

Supplement: Supplementary_Figure_S3_bbaf664 [file supplementary_figure_s3_bbaf664.jpeg]

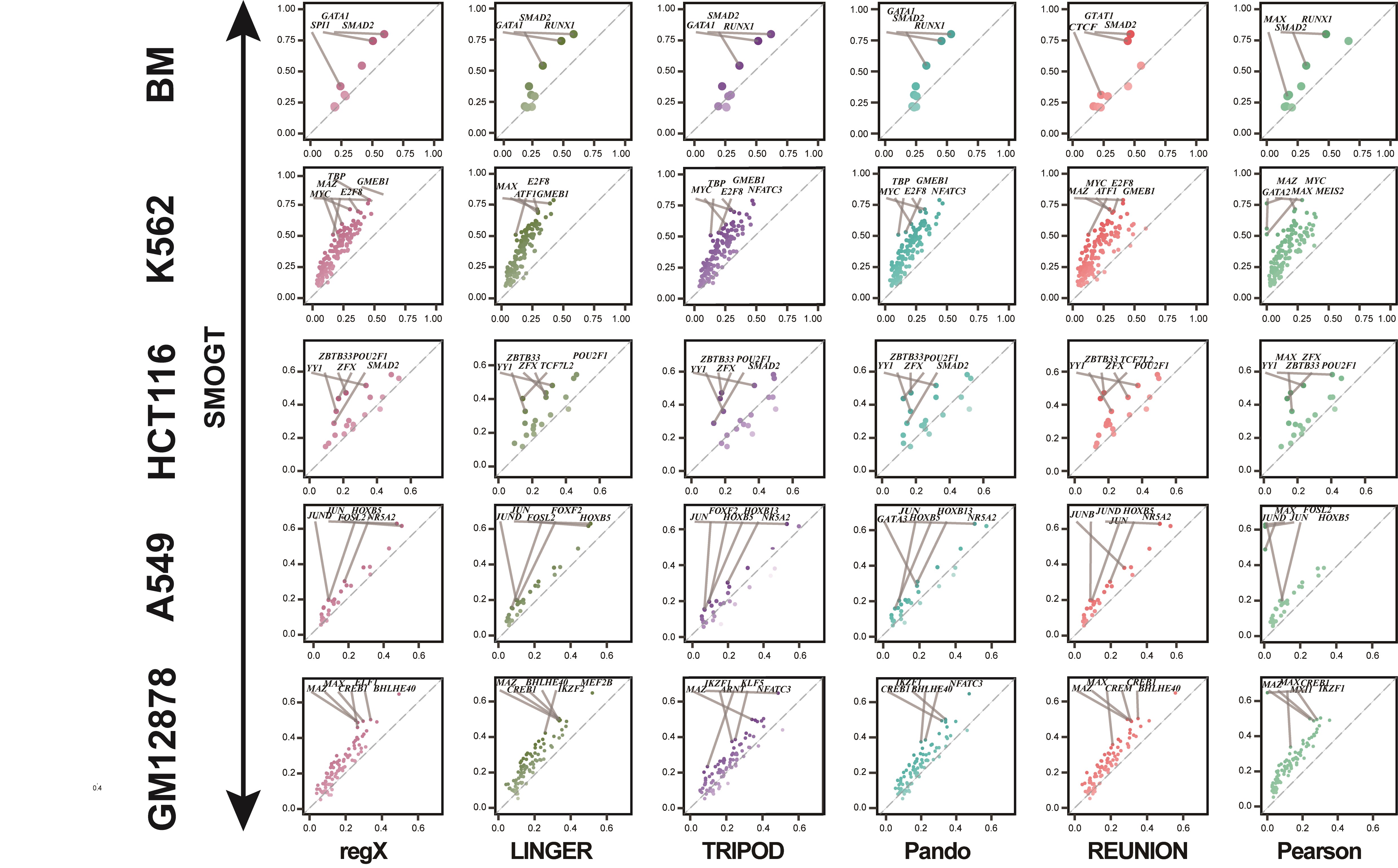

Supplement: Supplementary_Figure_S4_bbaf664 [file supplementary_figure_s4_bbaf664.jpeg]

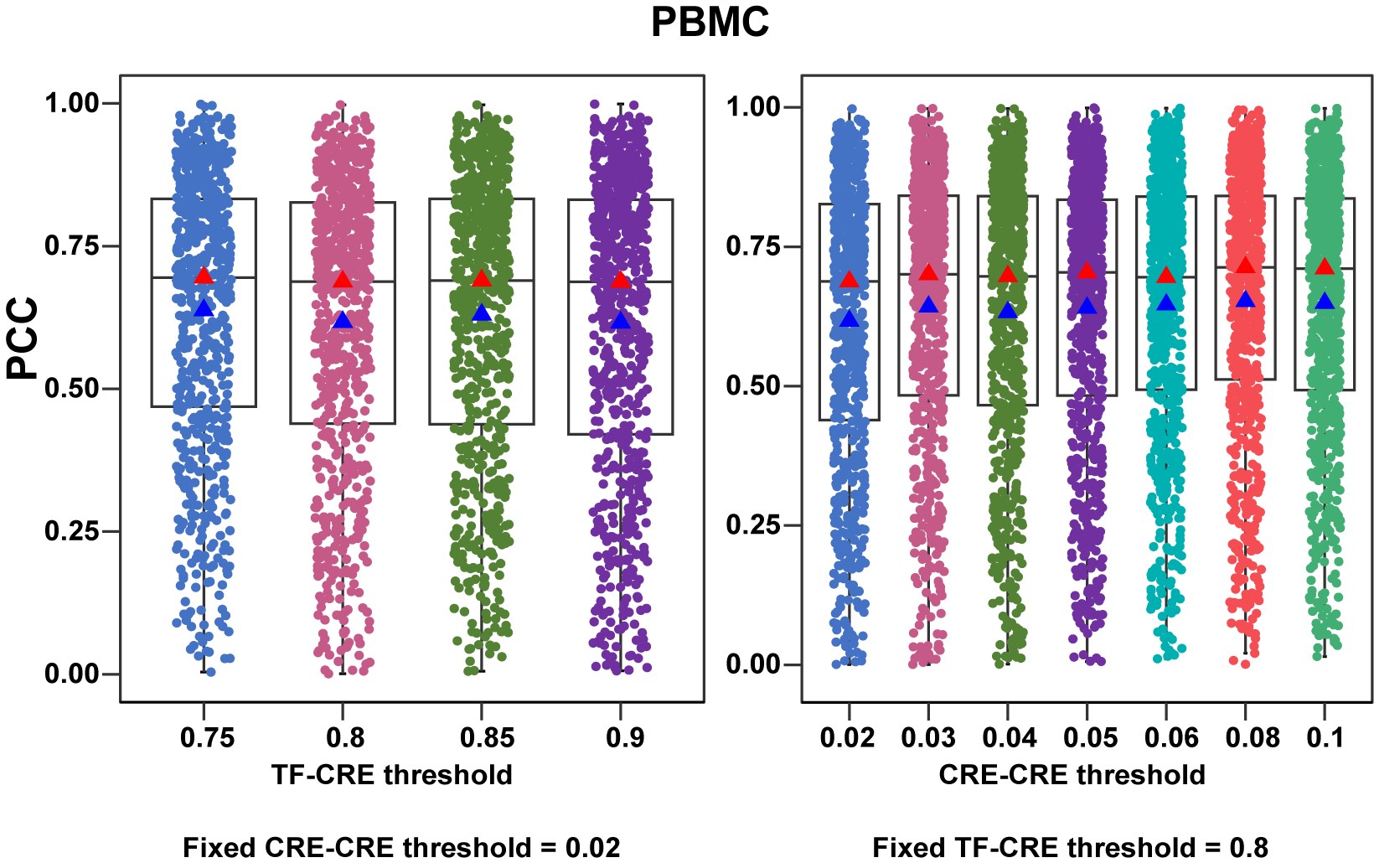

Supplement: Supplementary_Figure_S5_bbaf664 [file supplementary_figure_s5_bbaf664.jpeg]
