# Supplementary material for: Deciphering hierarchical regulatory network of cell fate via an epigenetics-informed heterogeneous graph transformer on single-cell multi-omics data
Source: Brief Bioinform. 2025 Dec 12;26(6):bbaf664. doi: 10.1093/bib/bbaf664 (PMC12875533; doi:10.1093/bib/bbaf664)
Supplement: Supplymentary_Table1_bbaf664 [file supplymentary_table1_bbaf664.docx]

# Summary of Datasets used in SMOGT

| **Dataset** | **Sequencing Platform** | **Tissue or cell type** | **Cell numbers**  **(processed)** | **Accession number/Link** |
| --- | --- | --- | --- | --- |
| SEACells infers transcriptional and epigenomic cellular states from single-cell genomics data  [Sitara Persad](https://pubmed.ncbi.nlm.nih.gov/?term=Persad+S&cauthor_id=36973557) 2023 | 10X  Genomics  droplet  experiments | Human bone marrow cells | 7548 (Remove immune cells) | GSE200046 |
| PBMC from a Healthy Donor - Granulocytes Removed Through Cell Sorting (10k) | 10X  Genomics  droplet  experiments | Human peripheral blood mononuclear cells | 11403 | https://www.10xgenomics.com/datasets/pbmc-from-a-healthy-donor-granulocytes-removed-through-cell-sorting-10-k-1-standard-2-0-0 |
| Multi-omic profiling of the developing human cerebral cortex at the single-cell level  Kaiyi Zhu etal.2023 | 10X  Genomics  droplet  experiments | Human Prefrontal cortex cells | 23745 | GSE204684 |
| OCI-AML22 multiome datasets (scRNA-Seq/scATAC-Seq) | 10X  Genomics  droplet  experiments | human primary AML cell derived OCI-AML22 | 7988 | GSE272187 |
| K562 | scCAT-seq | Human chronic myeloid leukemia cells | 74 | PMID: 30692544 |
| HCT116 | scCAT-seq | Human colorectal carcinoma cells | 90 | PMID: 30692544 |
| A549 | SNARE-seq2 | Human lung cancer cells | 1107 | GSE157660 |
| GM12878 | SNARE-seq2 | Human lymphoblastoid cells | 8986 | GSE157660 |
| K562 | direct-capture Perturb-seq | Human chronic myeloid leukemia cells | 162751 | https://zenodo.org/records/10044268 |
| Melanoma | scRNA-seq | Melanoma patient-derived cell line | 3754 | PMID: 32753671 |
| Melanoma | scATAC-seq | Melanoma patient-derived cell line | 936 | PMID: 37443338 |
