# Supplementary material for: Deciphering hierarchical regulatory network of cell fate via an epigenetics-informed heterogeneous graph transformer on single-cell multi-omics data
Source: Brief Bioinform. 2025 Dec 12;26(6):bbaf664. doi: 10.1093/bib/bbaf664 (PMC12875533; doi:10.1093/bib/bbaf664)
Supplement: Supplymentary_Table2_bbaf664 [file supplymentary_table2_bbaf664.docx]

# **The true TF-CRE relationships recorded in the ENCODE and ChIP-Altas databases**

| dataset | TF | Data source |
| --- | --- | --- |
| A549 | RAD21 NPAS2 NFE2L2 RCOR1 TEAD4 RAD21 POLR2AphosphoS2 GATA3 PBX3 MAX POLR2A CEBPB MYC BHLHE40 HEYL CTCF CEBPB MAX ZSCAN20 ZNF302 NR3C1 EP300 RAD21 FOSL2 JUN SMC3 ZC3H11A ZFP36 JUN MAZ MAFK SREBF1 SMC3 CTCF FOXF2 HOXA7 HOXB5 CTCF EP300 REST RNF2 CTCF KDM5A ESRRA E2F6 CHD2 SIN3A FOXS1 JUNB ELK1 JUN CEBPB CHD4 EHF HES2 ZNF624 TFCP2L1 ATOH8 HOXB13 CBX2 BCL3 RFX5 PBX1 RARB FOSL2 HDAC2 EHMT2 PRDM1 KDM1A TP63 CBX8 FOSB PHF8 NR2E3 HMGXB4 HMGA2 TP53 NR5A2 SREBF2 USF2 | ENCFF562UOF ENCFF678QTH ENCFF136LON ENCFF232TVP ENCFF329CUQ ENCFF146MNV ENCFF390AZA ENCFF761QLX ENCFF352MAF ENCFF652DIB ENCFF129FEV ENCFF486BXB ENCFF152LBQ ENCFF467CVV ENCFF109ADF ENCFF670UIE ENCFF634TZG ENCFF723DCX ENCFF143OQP ENCFF814JWH ENCFF469DNJ ENCFF624ZSR ENCFF702XIF ENCFF039TWK ENCFF786NAO ENCFF757QNZ ENCFF639RSC ENCFF016TWL ENCFF988QUL ENCFF486IUH ENCFF280RQS ENCFF155LGO ENCFF669UYG ENCFF507RLU ENCFF209ZVL ENCFF439SWX ENCFF679WUD ENCFF520POQ ENCFF641ZFM ENCFF341HVV ENCFF008SZM ENCFF796TMZ ENCFF992LDJ ENCFF201DBE ENCFF976HNC ENCFF169BSN ENCFF399ZUJ ENCFF719BHI ENCFF693LFI ENCFF669RAT ENCFF552VXR ENCFF133ZSB ENCFF307JCM ENCFF404ASR ENCFF708HLM ENCFF835PIH ENCFF699UTZ ENCFF507JZI ENCFF716VWY ENCFF455TWM |
| GM12878 | PRDM15 ATF3 RAD21 USF2 ETS1 MAX ZNF274 EP300 HCFC1 HSF1 CEBPB TARDBP ZFP36 ETV6 STAT1 TCF7 MYB NR2C2 FOXM1 BCL3 CREB1 SIN3A WRNIP1 PAX5 CHD1 REST SRF BRCA1 ESRRA EBF1 SRF STAT1 EP300 E2F4 MXI1 NRF1 JUND NFATC1 PBX3 NFIC PAX5 KAT2A CTCF EBF1 IKZF1 BATF CHD2 CTCF SP1 NFYA POLR2AphosphoS5 EGR1 MEF2A TBP CTCF MAZ RCOR1 BCL11A POLR2A ZZZ3 ELK1 POU2F2 SPI1 NFYB POLR3G STAT5A CEBPB USF1 MTA3 POLR2AphosphoS2 ELF1 PML MEF2C TCF12 FOS BCLAF1 EZH2 MAZ ZBTB4 RELA IRF3 GABPA ETV6 SUPT20H UBTF IRF3 RFX5 IRF4 TRIM22 ZNF207 EP300 ATF2 POLR2A RXRA YY1 TBL1XR1 ZEB1 YY1 SREBF2 MYC SMC3 JUND ATF7 PAX8 RELB IKZF2 EGR1 TCF3 TARDBP REST SIX5 TBX21 BHLHE40 CBX5 BACH1 BHLHE40 STAT3 SREBF1 CHD4 TAF1 CUX1 RUNX3 ZBTB33 ZBTB33 SUZ12 CBX3 JUNB IKZF1 MTA2 MLLT1 POLR2A CREM KLF5 IKZF2 BMI1 E2F8 HDGF ZBTB40 ZNF622 EED ATF2 YBX1 FOXK2 CEBPZ TRIM22 NBN ARNT ZBED1 E4F1 ASH2L ASH2L ASH2L SRF RAD51 SKIL KDM1A NFATC3 RAD21 IRF5 ZNF384 CTCF MAFK HDAC6 ZSCAN29 DPF2 ZNF687 MEF2B HDAC2 PKNOX1 RBBP5 ARID3A CBFB RB1 ZNF592 GATAD2B LARP7 NR2F1 NKRF SMARCA5 TCF12 SMAD5 ZNF217 ZNF24 ZBTB11 IKZF1 NFXL1 BCLAF1 ELF1 SMAD1 NR2C1 CTCF GABPA REST EP300 EP300 EP300 EP300 EP300 EP300 EP300 SPI1 ZBTB33 GABPA GABPA GABPA GABPA REST REST REST REST REST REST REST REST REST REST REST REST REST REST REST REST EP300 EP300 EP300 EP300 SPI1 SPI1 SPI1 SPI1 ZBTB33 ZBTB33 ZBTB33 ZBTB33 SUZ12 HDAC6 ZBTB33 ZBTB33 SRF SRF ARNT ARNT ZNF622 ZNF622 MEF2B MEF2B PAX8 PAX8 SKIL SKIL E2F8 E2F8 SMAD1 SMAD1 ZFP36 ZFP36 ATF2 ATF2 KLF5 KLF5 NFATC3 NFATC3 E4F1 E4F1 BRD4 RUVBL2 RUVBL2 TRIM28 TRIM28 TP53 | ENCFF157HQD ENCFF882AEU ENCFF940IMA ENCFF196JQW ENCFF568AZT ENCFF361EVH ENCFF034UEJ ENCFF707DWY ENCFF479QQB ENCFF663ODL ENCFF864EON ENCFF847AAV ENCFF627JQX ENCFF151UJT ENCFF973ACG ENCFF900GTF ENCFF705CGM ENCFF782KRV ENCFF549GKZ ENCFF452NTN ENCFF053UZX ENCFF013QNV ENCFF855UKX ENCFF827VVQ ENCFF753HYW ENCFF262MRD ENCFF114CWH ENCFF303GOQ ENCFF660PIB ENCFF895MHN ENCFF089YRX ENCFF896SEI ENCFF926AKK ENCFF744QAC ENCFF376AEL ENCFF864EBU ENCFF134BQO ENCFF172KBM ENCFF402DQD ENCFF980SDE ENCFF192WNJ ENCFF333PDI ENCFF827JRI ENCFF996MFL ENCFF432HLX ENCFF728KFD ENCFF351VXQ ENCFF796WRU ENCFF038AVV ENCFF937QTP ENCFF107UMF ENCFF637UJN ENCFF826GQU ENCFF099PYR ENCFF797SDL ENCFF467VSF ENCFF585BRC ENCFF824QXX ENCFF712JXT ENCFF535WUH ENCFF164MPE ENCFF934JFA ENCFF492ZRZ ENCFF156MUM ENCFF002CPJ ENCFF006GSY ENCFF955YFB ENCFF879TPT ENCFF475GKK ENCFF643YBS ENCFF146SYU ENCFF242KVU ENCFF238UKB ENCFF068YYR ENCFF571DGT ENCFF185BRL ENCFF339YTO ENCFF250FJC ENCFF880BEK ENCFF141SMI ENCFF887WLX ENCFF093KLR ENCFF342QUN ENCFF684BIN ENCFF014PHS ENCFF785MSW ENCFF601WHE ENCFF113VGD ENCFF519BGX ENCFF854NOE ENCFF718TWQ ENCFF973FKS ENCFF886PSD ENCFF063KLF ENCFF258UEW ENCFF276QRH ENCFF703XCL ENCFF446ZSX ENCFF896MPE ENCFF214XPD ENCFF837YJA ENCFF023QWA ENCFF277FJJ ENCFF934NVD ENCFF968MBN ENCFF511HZB ENCFF612EIU ENCFF700TAS ENCFF600LZY ENCFF975LZF ENCFF878FBM ENCFF170IZO ENCFF445XYV ENCFF864AYJ ENCFF631RZH ENCFF633YUQ ENCFF029PAA ENCFF397YRO ENCFF403JIP ENCFF502IPK ENCFF451AII ENCFF346JDW ENCFF917XFN ENCFF845VSZ ENCFF894BBO ENCFF670JSE ENCFF912OPT ENCFF819VMH ENCFF957SRJ ENCFF652GOE ENCFF535TAL ENCFF294IGP ENCFF992IBT ENCFF723ZRN ENCFF160PIE ENCFF961ZFW ENCFF626TYH ENCFF171FMR ENCFF359PUY ENCFF519PDZ ENCFF213LSA ENCFF488IGF ENCFF639XYN ENCFF639KLW ENCFF796RRA ENCFF511QTQ ENCFF596RIT ENCFF753UAX ENCFF855PXT ENCFF096XRG ENCFF917CXD ENCFF552EPA ENCFF432PLY ENCFF345VPL ENCFF506SOD ENCFF996NUD ENCFF002XEC ENCFF834GOT ENCFF478SRO ENCFF955FRU ENCFF951PEM ENCFF436SJS ENCFF588SBL ENCFF828NYA ENCFF003ZLG ENCFF734BGL ENCFF884QQW ENCFF581FMI ENCFF946JFA ENCFF474JTH ENCFF531CLQ ENCFF876CUT ENCFF844GNV ENCFF510ZBJ ENCFF963DVH ENCFF927IGC ENCFF118UKC ENCFF862GDZ ENCFF550FDV ENCFF154RAJ ENCFF485GWN ENCFF131ALE ENCFF664TYB ENCFF083GTV ENCFF448JWW ENCFF057NHJ ENCFF055QPX ENCFF479HJF ENCFF033MGN ENCFF626EEU SRX067503 SRX475743 SRX475747 SRX475748 SRX475749 SRX475750 SRX475751 SRX475752 SRX475753 SRX475754 SRX475755 SRX475756 SRX475757 SRX475758 SRX475759 SRX475760 SRX475773 SRX475774 SRX475775 SRX475776 SRX475777 SRX475778 SRX475779 SRX475780 SRX475781 SRX475782 SRX475783 SRX475784 SRX475785 SRX475786 SRX475787 SRX475788 SRX475789 SRX475790 SRX475791 SRX475792 SRX475793 SRX475794 SRX475795 SRX475796 SRX475797 SRX475798 SRX475799 SRX475800 SRX5090131 SRX5090184 SRX5457143 SRX5457144 SRX5457184 SRX5457185 SRX5457204 SRX5457205 SRX5457213 SRX5457214 SRX5457332 SRX5457333 SRX5457350 SRX5457351 SRX5457366 SRX5457367 SRX5457404 SRX5457405 SRX5457419 SRX5457420 SRX5457494 SRX5457495 SRX5457556 SRX5457557 SRX5457562 SRX5457563 SRX5457565 SRX5457566 SRX5457574 SRX5457575 SRX749133 SRX9424448 SRX9424449 SRX9823127 SRX9823128 ERX181469 |
| HCT116 | CTCF ZBTB33 ATF3 RAD21 REST POLR2A ZNF274 SIN3A POLR2AphosphoS5 YY1 TCF7L2 USF1 CTCF FOSL1 ELF1 CBX3 EGR1 EZH2phosphoT487 CTCF SRF MAX SP1 JUND TEAD4 ZFX EZH2 CEBPB MBD1 MBD2 MBD4 CBX3 CBX3 CBX3 CBX3 SUPT5H SUPT5H SUPT5H ATXN7L3 ATXN7L3 ATXN7L3 ATXN7L3 KAT2A KAT2A KAT2A KAT2A MED1 MED1 MED1 MED1 MED1 MED1 MED1 MED1 MED16 MED16 MED16 MED16 MED16 MED16 MED16 MED16 USP22 USP22 USP22 USP22 POU2F1 POU2F1 POU2F1 SMC2 SMC2 SMC2 SMC2 SMC2 SMC2 SMC2 SMC2 SMC2 SMC2 SMC2 SMC2 TP53 TP53 ZIC5 ZIC5 ZIC5 ZIC5 ZIC5 GFP ZIC5 ZIC5 ZIC5 ZIC5 TCF7L2 TCF7L2 TCF7L2 ZIC2 ZIC2 ZIC2 EPITOPE TAGS SUPT6H SUPT6H SUPT6H SUPT6H SUPT6H SUPT6H CTCF SMC1A RAD21 TP53 TP53 POLR2C POLR2C POLR2C POLR2C CDK9 CDK9 CDK9 CDK9 NELFE NELFE NELFE NELFE CBX5 CBX1 SUPT5H SUPT5H SUPT5H SUPT5H SUPT5H SUPT5H SUPT5H SUPT5H SUPT5H SUPT5H SUPT5H KAT7 KAT7 FANCD2 FANCD2 NEIL1 NEIL1 NEIL1 BACH1 BACH1 BMI1 BMI1 BRD1 BRD1 BRD1 BRD3 BRD3 BRD9 BRPF1 BRPF1 BRPF1 BRWD1 BRWD1 CBX1 CBX3 CBX5 CBX5 CIZ1 CXXC1 CXXC1 CXXC1 CXXC1 CXXC1 CXXC1 CXXC1 DNMT3A DNMT3L DPF1 DPF1 DPF1 DPF1 EZH2 FOS GTF2F2 GTF2F2 GTF2H2 GTF2H2C HMG20B HSF1 HSF1 HSF1 HSF1 HSF1 HSF1 HSF1 HSF1 HSF1 HSF1 HSF1 HSF1 HSF2 HSF2 JAZF1 JAZF1 JAZF1 JAZF1 KDM1A KDM1A KDM1A LDB1 MED1 MED1 MED21 MED21 MED22 MED22 MED30 MED30 MED4 MED6 MED7 MED7 MED8 MED8 MED9 MEN1 MEN1 NFE2L2 NFE2L2 NRF1 NRF1 NRF1 NRF1 NRF1 NRF1 NRF1 PCGF3 PCGF3 PCGF6 PCGF6 PRDM1 PRDM1 PRDM2 PRDM2 PRDM4 PRDM4 PRDM4 RCOR2 RCOR2 RCOR3 RCOR3 RNF20 SCML4 SCML4 SIRT1 SIRT1 SKIL SMAD2 SMAD2 SMAD4 SMAD4 SMAD4 SMAD4 SMAD4 SMAD4 SMAD4 SMAD4 SMAD4 SMARCAL1 SMARCAL1 SMARCAL1 SMARCB1 SMARCC1 SMARCC2 SMARCC2 SMARCD1 SMARCD1 SMARCE1 SMARCE1 SMARCE1 SNAPC4 SNAPC4 TCEAL8 TEAD3 TEAD4 TEAD4 TRIM24 USP7 WHSC1 WHSC1 YEATS2 YEATS4 BANP BANP MED1 MED1 MED1 MED1 RAD21 RUVBL2 RUVBL2 RAD51 POLQ POLH RAD51 POLQ POLH EPITOPE TAGS EPITOPE TAGS EPITOPE TAGS EPITOPE TAGS EPITOPE TAGS EPITOPE TAGS EPITOPE TAGS EZH2 EZH2 EPITOPE TAGS TP53 TP53 HNF4A TCF7L2 TCF7L2 TCF7L2 TCF7L2 HNF4A HNF4A TCF7L2 TCF7L2 TCF7L2 TCF7L2 HNF4A HNF4A EZH2 ZMYND8 ZMYND8 RUNX2 CNOT3 MED12 MED12 MED12 BRD4 BRD4 SREBF2 WDR5 MECP2 MECP2 CTCF SMC3 CTCF SMC3 | ENCFF832INR ENCFF141RIH ENCFF868BGR ENCFF391AAM ENCFF866PJC ENCFF848IHI ENCFF274VMH ENCFF493SWH ENCFF229YMU ENCFF451EDW ENCFF845TYG ENCFF725PAR ENCFF273SBR ENCFF936WPZ ENCFF420PYW ENCFF423XAH ENCFF822VKJ ENCFF760MKH ENCFF463FGL ENCFF502RNC ENCFF214KLO ENCFF600MKH ENCFF378LWF ENCFF008LFE ENCFF858YWR ENCFF809YPD ENCFF963FVR DRX021118 DRX021119 DRX021120 SRX055346 SRX055348 SRX055353 SRX055354 SRX4385550 SRX4385552 SRX4385554 SRX4937327 SRX4937328 SRX4937329 SRX4937330 SRX4937331 SRX4937332 SRX4937333 SRX4937334 SRX4937335 SRX4937336 SRX4937337 SRX4937338 SRX4937339 SRX4937340 SRX4937341 SRX4937342 SRX4937343 SRX4937344 SRX4937345 SRX4937346 SRX4937347 SRX4937348 SRX4937349 SRX4937350 SRX4937359 SRX4937360 SRX4937361 SRX4937362 SRX5123054 SRX5123055 SRX5123056 SRX5139483 SRX5139484 SRX5139485 SRX5139486 SRX5139487 SRX5139488 SRX5139489 SRX5139490 SRX5139491 SRX5139492 SRX5139493 SRX5139494 SRX5250886 SRX5250887 SRX5486412 SRX5486413 SRX5486414 SRX5486424 SRX5486425 SRX5486428 SRX5486429 SRX5486430 SRX5486433 SRX5486434 SRX5486437 SRX5486438 SRX5486439 SRX5486443 SRX5486444 SRX5486445 SRX5770333 SRX5771376 SRX5771377 SRX5771378 SRX5771379 SRX5771380 SRX5771381 SRX5880863 SRX5880864 SRX5880865 SRX603372 SRX603373 SRX6066490 SRX6066491 SRX6066492 SRX6066493 SRX6066502 SRX6066503 SRX6066504 SRX6066505 SRX6066510 SRX6066511 SRX6066512 SRX6066513 SRX6680405 SRX6680407 SRX6961665 SRX6961666 SRX6961667 SRX6961668 SRX6961669 SRX6961673 SRX6961674 SRX6961675 SRX6961678 SRX6961679 SRX6961680 SRX7057665 SRX7057667 SRX7228373 SRX7228374 SRX7415623 SRX7415624 SRX7415625 SRX8512723 SRX8512733 SRX8512734 SRX8512735 SRX8512736 SRX8512737 SRX8512738 SRX8512739 SRX8512740 SRX8512741 SRX8512742 SRX8512743 SRX8512744 SRX8512745 SRX8512746 SRX8512747 SRX8512748 SRX8512749 SRX8512750 SRX8512751 SRX8512752 SRX8512753 SRX8512754 SRX8512755 SRX8512756 SRX8512757 SRX8512758 SRX8512760 SRX8512761 SRX8512762 SRX8512763 SRX8512764 SRX8512765 SRX8512766 SRX8512767 SRX8512768 SRX8512769 SRX8512770 SRX8512771 SRX8512772 SRX8512773 SRX8512774 SRX8512775 SRX8512776 SRX8512777 SRX8512778 SRX8512779 SRX8512780 SRX8512781 SRX8512782 SRX8512783 SRX8512784 SRX8512785 SRX8512786 SRX8512787 SRX8512788 SRX8512789 SRX8512790 SRX8512791 SRX8512792 SRX8512793 SRX8512794 SRX8512795 SRX8512796 SRX8512797 SRX8512798 SRX8512799 SRX8512800 SRX8512801 SRX8512802 SRX8512803 SRX8512804 SRX8512805 SRX8512806 SRX8512807 SRX8512808 SRX8512809 SRX8512810 SRX8512811 SRX8512815 SRX8512816 SRX8512817 SRX8512818 SRX8512819 SRX8512820 SRX8512821 SRX8512822 SRX8512823 SRX8512824 SRX8512825 SRX8512826 SRX8512827 SRX8512828 SRX8512829 SRX8512830 SRX8512831 SRX8512832 SRX8512833 SRX8512834 SRX8512835 SRX8512836 SRX8512837 SRX8512838 SRX8512839 SRX8512840 SRX8512841 SRX8512842 SRX8512843 SRX8512844 SRX8512845 SRX8512846 SRX8512847 SRX8512848 SRX8512849 SRX8512850 SRX8512851 SRX8512852 SRX8512853 SRX8512854 SRX8512855 SRX8512856 SRX8512857 SRX8512858 SRX8512859 SRX8512860 SRX8512861 SRX8512862 SRX8512863 SRX8512864 SRX8512865 SRX8512866 SRX8512867 SRX8512868 SRX8512869 SRX8512870 SRX8512871 SRX8512872 SRX8512873 SRX8512874 SRX8512875 SRX8512876 SRX8512877 SRX8512878 SRX8512879 SRX8873455 SRX8873456 SRX8998070 SRX8998072 SRX8998074 SRX8998076 SRX9410586 SRX9424451 SRX9424452 DRX069273 DRX069274 DRX069275 DRX090760 DRX090761 DRX090762 SRX3781811 SRX3781812 SRX3781813 SRX3781814 SRX3781815 SRX3781816 SRX3781817 SRX5090126 SRX5090143 SRX527885 SRX5394956 SRX612780 SRX748420 SRX748421 SRX748422 SRX748423 SRX748424 SRX748425 SRX748426 SRX748427 SRX748428 SRX748429 SRX748430 SRX748433 SRX748434 SRX825988 SRX8470138 SRX8470139 SRX8655727 SRX886405 SRX9721691 SRX9721692 SRX9721693 SRX9721698 SRX9721699 SRX9912225 SRX5346136 SRX6747999 SRX6748000 SRX8582167 SRX8582170 SRX8582171 SRX8582174 |
| K562 | ZNF232 HDAC1 RBBP5 E2F4 E2F6 JUNB RFX5 USF1 YY1 UBTF SUZ12 SPI1 TAF1 ELK3 JUND POLR2AphosphoS2 CHD7 YY1 NFE2 DEAF1 ZNF75A GATA2 TFE3 TFCP2 MAZ ZNF57 HDAC2 CHD1 E2F4 FOXP4 AFF4 ZNF217 POLR2AphosphoS5 NFYA ZNF224 NCOR1 GTF2F1 TAF7 SP2 ZFP91 LCOR KAT7 TRIM28 ZFX ZNF184 SIRT6 ZNF282 SMAD4 IRF2 RAD21 MITF ZNF398 ZNF699 HSF4 CREM ZNF449 MEIS2 JUN SMAD1 TARDBP ETV6 BRF2 POU5F1 POLR3G REST RAD21 ZNF277 CREB3L1 KDM5B NFYB SMC3 BDP1 E2F6 CTCF POLR2A ZNF354C CTCFL TEAD4 CTCF ZNF274 ELK1 HDAC2 PREB ZBTB43 IRF2 HDAC6 ZBTB7A KAT2B USF2 ZNF274 POLR2A SIN3A SRF TAL1 CREBBP TBP NELFE SMARCB1 CEBPB FOS GATA1 MYC MAZ ZNF317 ELF4 ZNF263 TFDP1 GTF3C2 CCNT2 TBL1XR1 SMAD3 BHLHE40 ATF2 JUN HCFC1 SIX5 ATF3 TOE1 ETV6 ZBTB49 TGIF2 MYC BRF1 SMARCA4 CUX1 ZNF140 REST KDM2B CREB1 HMG20A KDM1A MYNN GATA2 IRF1 SAP30 ZNF384 NR2C2 PML JUND ZBTB34 POLR2A ZNF263 ZNF609 MAFF ELF1 SP1 REST HMGN3 MAX SP1 FOXP1 MTF1 CREB1 PHF8 EGR1 SIRT6 CBX8 ELF1 SETDB1 SIX5 POLR2AphosphoS2 WHSC1 BCLAF1 GATA2 CBX3 MAX CBX2 NRF1 EZH2 ZKSCAN8 ZNF124 ZNF740 MLX CEBPG ATF1 ZNF121 GABPB1 RERE ZNF431 ERF ZBTB33 MYBL2 NFYA HMGXB4 ETV6 NR2C2 DMTF1 ZNF512 PURB ZBTB1 NR3C1 STAT6 MXD1 FOXK1 E2F1 ATF3 MED1 ZFP30 SLC30A9 E2F5 ZNF143 POLR2A SETDB1 ZBTB40 MYNN ZNF639 POLR3A DLX4 TCFL5 ARID4B IFI16 USF1 TCF15 TFAP4 ID3 ZNF281 ARID4B TEAD4 MAX ZNF511 TEAD1 PPARD FOXA3 ZBTB11 THAP12 FOXA3 RAD21 USF2 ZNF561 CGGBP1 TBPL1 CHCHD3 ZNF707 TEAD1 ZNF778 SMAD3 RAD21 ZBTB17 ERF FOXO4 ZNF586 STAT5B SNAPC5 JUNB HMG20A ATF4 ZNF436 ZBTB9 ZNF347 ZNF212 REST ZNF695 ZNF257 ZFP1 ZC3H4 RNF2 ZKSCAN1 ZFP36 SMAD2 ZBED1 KLF10 NEUROD1 SMAD4 ZSCAN32 SMAD5 ZBTB33 CTCF ETS1 JUN ATF1 NFE2 SOX6 NR4A1 XRCC4 MXI1 SUZ12 EP300 UBTF STAT5A CHD4 HDAC8 ESRRA CBX3 MAX NRF1 MAFK NONO NONO NONO NR2F2 THAP1 YY1 TBL1XR1 E2F1 ZEB2 NRF1 BMI1 SIN3A SP1 CUX1 TCF7L2 PHB2 ARNT ELF1 ZMIZ1 ZNF512 TRIM24 MCM2 MCM7 SREBF1 MTA2 JUNB ETS2 HLTF ZNF740 SMARCC2 ZNF24 U2AF1 U2AF1 U2AF1 DDX20 RBM14 RBM14 RBM14 ZHX1 PYGO2 RNF2 MEF2A DEAF1 MYNN BRD9 ATF1 ZNF318 ARID1B MCM7 MYBL2 RFX1 ZNF407 RUNX1 ZNF24 HDGF HDAC1 LEF1 SKIL IRF1 IKZF1 PKNOX1 ASH1L FOXK2 CREB3 TARDBP YBX1 ZMYM3 NR4A1 E2F8 SNIP1 RAD51 ZSCAN29 SMARCE1 GABPB1 ARNT FOXK2 ZBTB33 ELK1 ZNF24 NFRKB MLLT1 HES1 SRSF3 SRSF3 SRSF3 TSHZ1 FIP1L1 FIP1L1 FIP1L1 E2F3 ZNF445 CLOCK ZNF583 HMG20B ZNF696 GTF2F1 GTF2F1 GTF2F1 SAFB2 SAFB2 SAFB2 ARID3B ZNF134 ZNF319 ZNF165 NRF1 CBX1 XRCC5 XRCC5 XRCC5 BCL6 KLF6 ZNF444 ZNF644 GABPA BRCA1 TFDP1 CEBPG VEZF1 RNF2 RBM25 RBM25 RBM25 E2F5 FOSL1 CEBPB EP300 ARID3A SETDB1 ILK GTF2B ATF3 MNT MNT MYC ZBTB11 HDAC8 CTCF CHAMP1 PBX2 ZNF589 USF2 PBX2 ZNF507 RUNX1 ZNF766 ZNF830 ZNF740 CSDE1 GTF2E2 NR2C2 PRMT5 NR3C1 ZNF184 ZC3H8 ZNF354B ZBTB5 MCM5 FOXJ2 POLR2A ZNF318 E4F1 MCM7 CTBP1 MGA TOE1 NKRF HDAC1 LARP7 TRIM25 TRIM25 TRIM25 NR2C1 HDGF CEBPZ HMBOX1 HMBOX1 HMBOX1 CHD2 GMEB1 CBX5 NFE2 KDM1A ZNF3 ZNF3 ZNF3 SMARCA4 THRA IRF9 PTTG1 TCF12 ATF1 XRCC3 COPS2 KDM4B TCF7 PCBP2 PCBP2 PCBP2 PCBP1 PCBP1 PCBP1 C11orf30 POLR2H GTF2A2 CDC5L ZC3H8 FOXA1 ZNF77 ZKSCAN3 ZNF316 ZNF24 SAFB SAFB SAFB NFRKB HIVEP1 BCOR TRIM24 KDM4B ZNF717 ZNF174 SRSF7 SRSF7 SRSF7 NCOR1 MCM5 TRIM28 NCOA2 NCOA2 ZNF215 ZNF184 DNMT1 ZBTB5 NR2F6 ZNF764 MCM3 MCM2 TCF12 ZNF239 ZNF639 RREB1 NR0B1 THAP7 ZBTB12 ELF4 AGO1 AGO1 AGO1 ZNF7 SRSF9 SRSF9 SRSF9 MLLT1 CCAR2 CCAR2 CCAR2 HNRNPH1 HNRNPH1 HNRNPH1 NR2F1 RLF HNRNPL HNRNPL HNRNPL ATF2 L3MBTL2 BCLAF1 NFATC3 ZEB2 RELA FOXM1 NCOR1 MAFG ZNF282 BCLAF1 NCOA1 ZBTB8A ZBTB8A ZBTB8A YBX3 ATF4 NCOA1 MIER1 EP400 ETV1 SUPT5H CXXC5 NFIC PRPF4 PRPF4 PRPF4 HOMEZ NFXL1 RNF2 ZNF639 ZNF316 POLR2G POLR2G POLR2G RBM39 RBM39 RBM39 TAF15 TAF15 TAF15 E2F7 RBFOX2 RBFOX2 RBFOX2 TRIP13 ZNF76 CBFA2T2 HNRNPUL1 HNRNPUL1 HNRNPUL1 NUFIP1 DMBX1 ATF3 ESRRB TEAD2 NCOA6 ZBTB40 ZNF639 NFIX ZNF83 RBM22 RBM22 RBM22 RBM17 RBM17 RBM17 RBM15 RBM15 RBM15 CHAMP1 DIDO1 ARNT ZFX MEF2D KDM1A GATAD2A EGR1 PHF20 ATF3 ZNF146 ZNF146 ZNF146 HDAC3 CSDE1 POLR2A BRD4 RBM34 RBM34 RBM34 ZBTB40 TAL1 NCOR1 ZNF197 SMARCA4 ZNF280A CEBPB RNF2 ZZZ3 ZBTB2 CC2D1A SMARCA5 ZMIZ1 CTCF IKZF1 RCOR1 RCOR1 BACH1 KLF16 GATA1 ZC3H11A GABPA KAT8 ZBTB11 U2AF2 U2AF2 U2AF2 ZSCAN29 NCOA1 ZNF407 FUS FUS FUS ZNF41 EHMT2 ZNF700 SIN3B ZNF408 HNRNPK MTA2 MBD2 DPF2 ZFP91 ZNF12 RNF219 MBD1 ZBTB26 MNT MITF RFX1 MAZ NBN ATF6 DPF2 AFF1 AFF1 POLR2B TRIM25 TCF3 ID3 KHSRP BACH1 HDAC2 THRAP3 EWSR1 E2F1 GMEB1 ZNF175 DDX20 ZNF79 ZKSCAN8 ZNF395 ZNF830 KLF13 CEBPG NR2C1 ADNP NONO NR4A1 ARID2 ELF1 NCOA4 ZNF354B HDAC2 ZNF785 ZNF23 HDAC1 HEY1 ZNF133 ARHGAP35 ZNF584 FOSL1 MTA3 ZNF655 GATA2 ZNF311 CBFA2T3 ZNF148 ZNF397 ZNF507 HNRNPLL HNRNPLL HNRNPLL TAF9B ZNF324 KLF1 MTA1 SNRNP70 SNRNP70 SNRNP70 RB1 GTF2F1 DACH1 PRDM10 TARDBP TARDBP TARDBP PHF21A PTBP1 PTBP1 PTBP1 NR1H2 RBPJ ZNF551 NR2F6 ZNF592 GATAD2B NFATC3 FOXJ3 ILF3 HOXB6 ZNF780A NR2F6 ETV5 SRF HBP1 NR3C1 ZNF668 NFXL1 PHB MLX PTRF ZNF518B ZBTB11 E4F1 ELF4 GTF2I TFAM CBFB BRCA1 RHOXF2B RFX7 TRIM28 ATF7 ZNF280B ZFPM2 HINFP TSC22D4 TAF7 NFE2L1 CAMTA2 PYGO2 PATZ1 SRF FOXM1 ZNF3 MYC SRSF1 SRSF1 SRSF1 EGR1 SFPQ SFPQ LEF1 TBX18 ZNF830 ZNF84 PHTF2 DDIT3 OTX1 ZNF253 SREBF2 GABPB2 ELF2 MECOM STAG1 THRB CREB5 TRIM28 TRIM28 CTCF CTCF CTCF CTCF CTCF CTCF CTCF CTCF CTCF CTCF CTCF CTCF MED1 CTCF CTCF CTCF CTCF CTCF CTCF ZNF263 ZNF263 GATA1 GATA2 JUN FOS MYC L3MBTL2 E2F6 PLAG1 PLAG1 USF2 USF2 NELFA NELFE NELFE NELFA NELFA NELFA NELFE NELFE MAPK14 MAPK14 MAPK14 MAPK14 SRSF1 SRSF3 SRSF4 SRSF7 SRSF9 U2AF1 U2AF2 HNRNPC HNRNPH1 HNRNPK HNRNPL HNRNPLL HNRNPUL1 PTBP1 PCBP1 PCBP2 FUS TAF15 SNRNP70 PRPF4 FIP1L1 RBFOX2 RBM22 RBM25 RBM39 AGO1 SAFB SAFB2 NONO GTF2F1 SFPQ TARDBP XRCC5 CCAR2 BRD2 BRD2 BRD2 BRD2 BRD2 BRD2 BRD2 BRD2 BRD3 BRD3 BRD3 BRD3 BRD3 BRD3 BRD3 BRD3 BRD4 BRD4 BRD4 BRD4 BRD4 BRD4 BRD4 BRD4 TBP CBX8 CBX8 GFP GFP HOXB8 GFP GFP SMARCC2 SMARCC2 HDAC3 HDAC3 ATF3 ATF3 MCM2 MCM2 DACH1 DACH1 ZNF830 ZNF830 SMAD1 SMAD1 ARHGAP35 ARHGAP35 NFATC3 NFATC3 NUFIP1 NUFIP1 MCM7 MCM7 PHF20 PHF20 ETS2 ETS2 TAF9B TAF9B MCM5 MCM5 ILF3 ILF3 ZSCAN29 ZSCAN29 ASH1L ASH1L NCOA4 NCOA4 NCOA1 NCOA1 PRDM10 KDM4B KDM4B TRIM25 TRIM25 MCM2 MCM2 GABPB1 GABPB1 ARNT ARNT EWSR1 EWSR1 ATF4 ATF4 TRIP13 TRIP13 NCOA1 NCOA1 ZNF146 RLF RLF E2F1 E2F1 E4F1 E4F1 EHMT2 EHMT2 ZNF282 ZNF282 BRD9 BRD9 GTF2F1 GTF2F1 NR0B1 NR0B1 ZNF3 ZFP36 ZFP36 DPF2 DPF2 AFF1 AFF1 BCLAF1 BCLAF1 ZBTB8A ZBTB8A MITF MITF NCOA2 NCOA2 CHAMP1 CHAMP1 NR3C1 NR3C1 MEIS2 MEIS2 ZNF830 ZNF830 NCOA2 NCOA2 POU5F1 POU5F1 ZNF280A ZNF280A HDAC2 HDAC2 ZFP91 ZFP91 ZBTB5 ZBTB5 GMEB1 GMEB1 ZNF639 ZNF639 E2F8 E2F8 AFF1 AFF1 PYGO2 PYGO2 NEUROD1 NEUROD1 ZNF407 ZNF407 NFRKB NFRKB TRIM28 TRIM28 NR3C1 NR3C1 ZNF639 ZNF639 ZNF184 ZNF184 XPO1 ZZZ3 SUPT20H ZZZ3 SUPT20H IRF1 IRF1 MLLT3 CTCF CTCF CTCF CTCF CTCF CTCF CTCF CTCF CTCF CTCF CTCF CTCF CTCF CTCF CTCF CTCF TP53 TP53 TP53 TP53 TP53 TP53 TP53 TP53 TP53 TP53 TP53 TP53 TP53 TP53 TP53 TP53 CDAN1 GATA1 EPITOPE TAGS EPITOPE TAGS CAS9 CAS9 CTCF CTCF GATA1 GATA1 TAL1 TAL1 CAS9 CAS9 CTCF CTCF GATA1 GATA1 TAL1 TAL1 CAS9 CAS9 CTCF CTCF GATA1 GATA1 TAL1 TAL1 CAS9 CAS9 CTCF CTCF GATA1 GATA1 TAL1 TAL1 CAS9 CAS9 CTCF CTCF GATA1 GATA1 TAL1 TAL1 DDX39B DDX39B ZNF148 ZNF148 SAFB SAFB ZBTB40 ZBTB40 VEZF1 VEZF1 ZNF395 ZNF395 XRCC5 XRCC5 HNRNPH1 HNRNPH1 CCAR2 CCAR2 RBM39 RBM39 SRSF9 SRSF9 CAS9 CAS9 CTCF CTCF CTCF CTCF CTCF BRD2 BRD2 BRD2 BRD2 BRD3 BRD3 BRD3 BRD3 BRD4 BRD4 BRD4 BRD4 BRD2 BRD3 BRD4 BRD4 BRD4 BRD4 BRD4 LDB1 CBFA2T3 LDB1 CBFA2T3 SMC1B SMC1B STAG3 STAG3 HIF1A HIF1A HIF1A HIF1A CDC73 CDC73 CDC73 CDC73 CDC73 CDK8 SUMO2 SUMO2 SUMO2 SUMO2 SUMO2 SUMO2 SUMO2 SUMO2 SUMO2 SUMO2 SUMO2 SUMO2 EPITOPE TAGS EPITOPE TAGS RUVBL2 RUVBL2 TBP TBP TBP TBP TBP TBP TBP TBP GTF2A2 GTF2A2 GTF2A2 GTF2A2 GTF2B GTF2B GTF2B GTF2B KAT2A KAT2A CTCF TOX4 TOX4 PHF19 PHF19 PHF19 MTF2 MTF2 SMARCA4 SMARCA4 SMARCA4 THRA | ENCFF672PJF ENCFF669MJX ENCFF942VGF ENCFF064CWA ENCFF085GHX ENCFF362UGH ENCFF661EUX ENCFF310CCS ENCFF589PZO ENCFF568ZPW ENCFF647TBC ENCFF888CKG ENCFF784BTI ENCFF198NGY ENCFF273KIA ENCFF951KHS ENCFF722UJW ENCFF074OAM ENCFF714MVQ ENCFF030AXK ENCFF307UZC ENCFF497ISV ENCFF592NJN ENCFF392MAR ENCFF389FLV ENCFF945IST ENCFF182MPT ENCFF408NUX ENCFF221TRR ENCFF974IPP ENCFF142MJD ENCFF515LWL ENCFF060MMW ENCFF908HSL ENCFF905RSN ENCFF816AEF ENCFF075BRS ENCFF792CKI ENCFF741FZG ENCFF718IGO ENCFF266MUT ENCFF555IKI ENCFF172CRL ENCFF840NZE ENCFF882XTP ENCFF217ACS ENCFF094FBP ENCFF934NEX ENCFF559ODJ ENCFF258VXX ENCFF605XYC ENCFF380KJB ENCFF854SNX ENCFF023JGT ENCFF324ELP ENCFF443BGE ENCFF861ZJL ENCFF865UPM ENCFF120PGJ ENCFF880BPX ENCFF584QFY ENCFF944LWS ENCFF724DNU ENCFF307GHJ ENCFF761YYL ENCFF930WPG ENCFF382QUI ENCFF118GMS ENCFF807FNB ENCFF718ZFY ENCFF289LLT ENCFF284FTY ENCFF831NDQ ENCFF660GHM ENCFF921FKB ENCFF604RBX ENCFF630YVJ ENCFF501XJP ENCFF736NYC ENCFF045CFL ENCFF586MZX ENCFF630HGY ENCFF927VIS ENCFF124KCE ENCFF430YXJ ENCFF736FGN ENCFF346AOR ENCFF349VSP ENCFF744HVD ENCFF742DPO ENCFF107SJD ENCFF884MNF ENCFF101SEZ ENCFF852ZRK ENCFF532VPN ENCFF370YGS ENCFF265KKE ENCFF217LXF ENCFF882ARK ENCFF258PLH ENCFF657CTC ENCFF566CTX ENCFF837NNR ENCFF653DNY ENCFF518EGY ENCFF264BDD ENCFF044IDV ENCFF496PIQ ENCFF642ZYO ENCFF034SWM ENCFF335ZTU ENCFF154IVU ENCFF121HYT ENCFF190CGV ENCFF139FMU ENCFF754ZWP ENCFF718VHT ENCFF917HUH ENCFF965PER ENCFF308VAI ENCFF238XIA ENCFF608CXN ENCFF577LSK ENCFF486QCV ENCFF838GFC ENCFF207XCM ENCFF539MIO ENCFF702THZ ENCFF193LLN ENCFF866ARI ENCFF054XCG ENCFF603EAX ENCFF165ZEP ENCFF093BBG ENCFF383IEP ENCFF864XZP ENCFF495XYS ENCFF051FNO ENCFF306SZL ENCFF683VXW ENCFF410CPY ENCFF295XBK ENCFF824EJC ENCFF119AHD ENCFF688TNZ ENCFF412ERW ENCFF707MDI ENCFF615AKB ENCFF493DCP ENCFF171NEU ENCFF491EEI ENCFF478MAJ ENCFF970QKS ENCFF981ISM ENCFF566PRZ ENCFF821XJU ENCFF522HZT ENCFF763MXW ENCFF319ZYX ENCFF742ZHT ENCFF950YZE ENCFF862UUR ENCFF070ZTX ENCFF242YZU ENCFF068OEJ ENCFF171FVU ENCFF258XBJ ENCFF410RJD ENCFF804RVA ENCFF109UVJ ENCFF881MMD ENCFF959SMC ENCFF128YWS ENCFF558AJI ENCFF793HSJ ENCFF650DWZ ENCFF009RFC ENCFF473NCJ ENCFF442RYH ENCFF330EGV ENCFF763XBV ENCFF227LKX ENCFF786CRW ENCFF185KMK ENCFF405QTW ENCFF946CTR ENCFF505INE ENCFF817EJA ENCFF500BWO ENCFF916CTU ENCFF201BGD ENCFF598VGY ENCFF559MTF ENCFF516ZWP ENCFF904RTN ENCFF246USC ENCFF882ZEN ENCFF141HPL ENCFF441QXF ENCFF173QUY ENCFF596HNP ENCFF634JRD ENCFF752EMN ENCFF132DPY ENCFF749GMT ENCFF645OCW ENCFF627DJP ENCFF832DBU ENCFF410SWS ENCFF528IDR ENCFF518SJY ENCFF255QDL ENCFF415MKA ENCFF438IYI ENCFF794SCQ ENCFF648ORA ENCFF086FAZ ENCFF303CVC ENCFF266YHW ENCFF653ABW ENCFF214SNH ENCFF893YKY ENCFF197SXI ENCFF227HVU ENCFF206LMB ENCFF504WWL ENCFF330SHG ENCFF289ZIR ENCFF701RWL ENCFF771XZZ ENCFF927NEU ENCFF645IVF ENCFF959ZKZ ENCFF692HUD ENCFF165JQS ENCFF718DSJ ENCFF057JFH ENCFF292HSS ENCFF462ZIG ENCFF750AGR ENCFF887ZDT ENCFF122FTW ENCFF656THP ENCFF932KRN ENCFF118ATD ENCFF250MUC ENCFF318ZUN ENCFF588CXX ENCFF843NBV ENCFF435MHH ENCFF118ECK ENCFF493LZB ENCFF738IQL ENCFF795AWO ENCFF270TMW ENCFF409SDU ENCFF560UGR ENCFF839GAS ENCFF730QQD ENCFF392INU ENCFF142ZTD ENCFF625QHR ENCFF232ZHD ENCFF537HHU ENCFF971YTJ ENCFF146GZZ ENCFF769AUF ENCFF886BDQ ENCFF589QXC ENCFF206BGR ENCFF474PRJ ENCFF125GZU ENCFF772UYI ENCFF540QPM ENCFF068IGH ENCFF889QYR ENCFF702XPO ENCFF326CIT ENCFF187BVL ENCFF985QBS ENCFF611FTI ENCFF588QRH ENCFF386ZWO ENCFF822FKQ ENCFF777PKJ ENCFF439TJM ENCFF823CQK ENCFF134NMW ENCFF211TTD ENCFF847ZHF ENCFF823RYG ENCFF398UQZ ENCFF967URJ ENCFF053HPQ ENCFF242AOL ENCFF762CYI ENCFF600KEF ENCFF469KAH ENCFF553GPK ENCFF136KLM ENCFF732ZNB ENCFF785QJM ENCFF728ITJ ENCFF350OMH ENCFF316IYH ENCFF184IOY ENCFF527MLG ENCFF239WEU ENCFF519LSA ENCFF029RBI ENCFF080IBW ENCFF328OCA ENCFF772QLT ENCFF614IBI ENCFF004YCK ENCFF908MWB ENCFF268YOM ENCFF730URE ENCFF482DRO ENCFF752DQV ENCFF746TUQ ENCFF465UMU ENCFF682SIY ENCFF092DLN ENCFF175YPM ENCFF335VOJ ENCFF060JVZ ENCFF031NTF ENCFF813TRY ENCFF516ZEQ ENCFF775WSL ENCFF627RSK ENCFF990SFN ENCFF879NTL ENCFF674ZQI ENCFF637PFI ENCFF768VRG ENCFF255HEF ENCFF374EFU ENCFF683VTL ENCFF621FPC ENCFF245OJC ENCFF591TLJ ENCFF067KCK ENCFF557FUM ENCFF711ILQ ENCFF099RDJ ENCFF553IUR ENCFF702AQS ENCFF950IYB ENCFF986BYT ENCFF286IPW ENCFF117XRE ENCFF852ZIK ENCFF925GLW ENCFF020XNM ENCFF536WBT ENCFF407STM ENCFF148YMC ENCFF764HIS ENCFF553BVI ENCFF768QMM ENCFF917RIN ENCFF715WGN ENCFF225GCF ENCFF057NNR ENCFF556MUV ENCFF671ZGP ENCFF722XRW ENCFF926XGK ENCFF048BKZ ENCFF144ZLB ENCFF883WYX ENCFF084DTV ENCFF285TMA ENCFF710UAZ ENCFF416TFM ENCFF648UJW ENCFF053ABJ ENCFF099OSI ENCFF899BJR ENCFF843UHP ENCFF246VJH ENCFF225ZPU ENCFF087DKT ENCFF624NUZ ENCFF176MDV ENCFF270TSN ENCFF169AWH ENCFF873VFI ENCFF169QYL ENCFF259YUE ENCFF470CSE ENCFF929TWP ENCFF998KKJ ENCFF708IYN ENCFF941EDY ENCFF563ZJM ENCFF295XCB ENCFF773XPT ENCFF889XXJ ENCFF258GDS ENCFF362JHQ ENCFF998LIR ENCFF433ERT ENCFF923GDR ENCFF102XVH ENCFF680WBN ENCFF849VEO ENCFF082QHP ENCFF004HXL ENCFF022KBK ENCFF821MKR ENCFF891OQP ENCFF267QRI ENCFF143MEF ENCFF987FJR ENCFF895RNS ENCFF461QQT ENCFF986SXD ENCFF675PHY ENCFF991VAW ENCFF566PEY ENCFF582SNT ENCFF541VYN ENCFF345BUG ENCFF345IHK ENCFF640ZIN ENCFF841RMH ENCFF072JDK ENCFF003LPE ENCFF466PKS ENCFF672NBD ENCFF219NIA ENCFF746OAD ENCFF564KIU ENCFF263VIC ENCFF018TNP ENCFF960SMD ENCFF266FUJ ENCFF230DZT ENCFF649UCX ENCFF460GXA ENCFF344LTH ENCFF957CVJ ENCFF842JME ENCFF545WAL ENCFF683WRK ENCFF239OHL ENCFF782PIZ ENCFF524ZER ENCFF563WUP ENCFF068MGV ENCFF507QLB ENCFF304PQQ ENCFF574LAO ENCFF514DDG ENCFF448LKL ENCFF469ZBB ENCFF633XFQ ENCFF588GNU ENCFF718DFX ENCFF558DSF ENCFF672ZQW ENCFF947AEO ENCFF863WFD ENCFF066OJB ENCFF023IFO ENCFF346UGW ENCFF510PVQ ENCFF570YIC ENCFF180XUM ENCFF793FMG ENCFF992HUS ENCFF724CHN ENCFF918BZG ENCFF504PFQ ENCFF924GPK ENCFF137OGC ENCFF942JZR ENCFF928UFW ENCFF736XUU ENCFF941XZW ENCFF881XQF ENCFF732IRT ENCFF467RYH ENCFF941RVL ENCFF956MGE ENCFF038WQY ENCFF084KHS ENCFF920CRL ENCFF408RSJ ENCFF045AOZ ENCFF497OQD ENCFF809JNC ENCFF697DRN ENCFF840SLB ENCFF649OPX ENCFF478MPX ENCFF411YVY ENCFF537RNU ENCFF321LMQ ENCFF449JMB ENCFF124FTW ENCFF597YMB ENCFF304PGC ENCFF159END ENCFF822FXR ENCFF059WVE ENCFF550VUN ENCFF217MBF ENCFF898CGY ENCFF282KHW ENCFF770JQM ENCFF872RQG ENCFF057SID ENCFF922RHM ENCFF058VGZ ENCFF412WOK ENCFF079EGS ENCFF835FPJ ENCFF049MDN ENCFF583XAW ENCFF147IAH ENCFF808QUD ENCFF569TJP ENCFF669VJB ENCFF057RJK ENCFF931LUC ENCFF372YFU ENCFF830MTX ENCFF616FCV ENCFF836VRV ENCFF100VYA ENCFF794IRP ENCFF018HWM ENCFF217HAW ENCFF137IBM ENCFF746IEZ ENCFF355OYM ENCFF704PGT ENCFF583GKE ENCFF657LFS ENCFF292JRY ENCFF844QFF ENCFF797TVO ENCFF746GDG ENCFF752VQB ENCFF010STZ ENCFF984ESZ ENCFF854WAP ENCFF192ASP ENCFF063NIH ENCFF941SRW ENCFF972OBR ENCFF475CIS ENCFF936WES ENCFF711ZED ENCFF321NXM ENCFF493DXA ENCFF110GDP ENCFF725JXZ ENCFF660DEP ENCFF589EVD ENCFF328SSL ENCFF423CWQ ENCFF877ZKU ENCFF086TAD ENCFF904YZI ENCFF562XKK ENCFF167CQF ENCFF065RZP ENCFF738XMN ENCFF798LLI ENCFF169MAE ENCFF785ACI ENCFF417RQZ ENCFF886UMM ENCFF601LMD ENCFF341CNM ENCFF271MJE ENCFF217EJG ENCFF451AEQ ENCFF273EYJ ENCFF283CUY ENCFF413LLO ENCFF209JJD ENCFF503DIK ENCFF983LFS ENCFF617CAZ ENCFF710LLF ENCFF547PES ENCFF938ZPZ ENCFF120IDE ENCFF232ASB ENCFF538ACI ENCFF955QCD ENCFF305XQM ENCFF543GET ENCFF768TJI ENCFF991ZSC ENCFF700VSW ENCFF669RLC ENCFF359ZQQ ENCFF556SMM ENCFF106ECV ENCFF209WPT ENCFF996OEL ENCFF803CYY ENCFF643AUL ENCFF726LLI ENCFF451LLC ENCFF420IBN ENCFF522JUV ENCFF987DPX ENCFF861YKK ENCFF056OIG ENCFF937QYU ENCFF563WDZ ENCFF971VJZ ENCFF091TCH ENCFF515GUE ENCFF928BHE ENCFF075OIT ENCFF910JTR ENCFF242ULW ENCFF829FZW ENCFF295YRT ENCFF136DDO ENCFF053PDX ENCFF107DBQ ENCFF742XBE ENCFF296DCP ENCFF932FVX ENCFF775PIY ENCFF079ERC ENCFF355MNE ENCFF130JVF ENCFF670ILH ENCFF782GWS ENCFF071GJH ENCFF624PTB ENCFF101DBG ENCFF836PGX ENCFF987UBO ENCFF267OGF ENCFF932ZCO ENCFF712ZNR ENCFF408XPG ENCFF797VEK ENCFF209DVO ENCFF156GHD ENCFF722RWS ENCFF881DAT ENCFF221SKA ENCFF637SIR ENCFF627PLM ENCFF224YBQ ENCFF026BDA ENCFF488OTN ENCFF509ZLE ENCFF528SIV ENCFF489EME ENCFF113LBV ENCFF554GSE ENCFF398EQF ENCFF134HBP ENCFF164CTH ENCFF151WYQ ENCFF514WJW ENCFF015FXW ENCFF688ARM ENCFF142CPK ENCFF581MLB ENCFF629EAD ENCFF189OHQ ENCFF208OME ENCFF257NOX ENCFF207MLR ENCFF505RNR ENCFF478LQC ENCFF788YHU ENCFF716PXH ENCFF835PDO ENCFF021JCJ ENCFF417FFL ENCFF313VEF ENCFF435SNP ENCFF440AQF ENCFF076GSK ENCFF820GPR ENCFF704UVS ENCFF670POK ENCFF863ZFH ENCFF542HJS ENCFF674XTY ENCFF195YGC ENCFF821QOS ENCFF110HVI ENCFF773RNU ENCFF375SIS ENCFF525XXS ENCFF678TXX ENCFF652ZZF ENCFF067FJF ENCFF924FYI ENCFF104VFF ENCFF365ETH ENCFF192MEM ENCFF748EAO ENCFF670RLH ENCFF908SNB ENCFF430IPX ENCFF896IUI ENCFF453MMH ENCFF456USL ENCFF199GFU ENCFF739AJO ENCFF318GYM ENCFF137TCL ENCFF913WRW ENCFF133TSU ENCFF067BCD ENCFF712NHB ENCFF150FJT ENCFF352NGK ENCFF063VRD ENCFF432KJA ENCFF180KAV ENCFF810OHB ENCFF952WKN ENCFF005MBI ENCFF256NXT ENCFF280AIK ENCFF156EZP ENCFF772OKO ENCFF080KWE ENCFF082DOH ENCFF283UWH ENCFF193BGB ENCFF878SVX ENCFF122QSN ENCFF662WPN ENCFF833ZNA ENCFF432HRL ENCFF265MQC ENCFF674KVR ENCFF058ZHN ENCFF033KXY ENCFF206MJS ENCFF306TAD ENCFF479SOJ ENCFF837ZJJ ENCFF729DNM ENCFF405BKB ENCFF905VXX ENCFF448YOS ENCFF564QOL ENCFF215JWS ENCFF694BIA ENCFF917HXV ENCFF835NOD ENCFF798KXN ENCFF691JZW ENCFF913YMX ENCFF647RGI ENCFF847QJI ENCFF388KGW ENCFF778UHP ENCFF567CPM ENCFF382CUT ENCFF285MHB ENCFF794OSY ENCFF529SRS ENCFF604LXR ENCFF716OTE ENCFF681YLP ENCFF961ADR ENCFF885HJI ENCFF201WFM ENCFF424OWM ENCFF171ZNN ENCFF491UBF ENCFF096AYV ENCFF539GWU ENCFF439KTZ ENCFF832HNJ ENCFF866OZW ENCFF269PAM ENCFF802NHC ENCFF620FIH ENCFF727JXN ENCFF774NZZ ENCFF634OOU ENCFF951BFN ENCFF253UAF ENCFF549NPY ENCFF826QOG ENCFF705SFR ENCFF199CQK ENCFF366XDW ENCFF123IHX ENCFF232TVE ENCFF743JJF ENCFF087EVW ENCFF820MJD ENCFF798AFV ENCFF114VAI ENCFF798URE ENCFF862DEO ENCFF792SXS ENCFF640AKG ENCFF757ODD ENCFF652ZEN ENCFF689HWD ENCFF405EGZ ENCFF150ZBY ENCFF014HYS ENCFF130WQT ENCFF869RFC ENCFF245JPK ENCFF019NGO ENCFF710YFH ENCFF103YZQ ENCFF695PDY ENCFF188PLS ENCFF921BXP ENCFF906PUB ENCFF875JMR DRX402530 DRX402531 ERX1149057 ERX1149058 ERX1149072 ERX1149073 ERX1149087 ERX1149088 ERX1149102 ERX1149103 ERX1149117 ERX1149118 ERX2868715 ERX2868716 ERX626785 ERX989278 ERX989280 ERX989288 ERX989290 ERX989298 ERX989300 SRX013323 SRX013324 SRX014810 SRX014811 SRX015140 SRX015141 SRX015142 SRX059371 SRX059373 SRX3768261 SRX3768262 SRX3768263 SRX3768264 SRX3852890 SRX3852891 SRX3852892 SRX3852895 SRX3852897 SRX3852898 SRX3852899 SRX3852900 SRX4643712 SRX4643713 SRX4643714 SRX4643715 SRX4708061 SRX4708062 SRX4708063 SRX4708064 SRX4708065 SRX4708066 SRX4708067 SRX4708068 SRX4708069 SRX4708070 SRX4708071 SRX4708072 SRX4708073 SRX4708074 SRX4708075 SRX4708076 SRX4708077 SRX4708078 SRX4708079 SRX4708080 SRX4708081 SRX4708082 SRX4708083 SRX4708084 SRX4708085 SRX4708086 SRX4708087 SRX4708088 SRX4708089 SRX4708090 SRX4708092 SRX4708093 SRX4708094 SRX4708095 SRX4781465 SRX4781466 SRX4781467 SRX4781468 SRX4781469 SRX4781470 SRX4781471 SRX4781472 SRX4781481 SRX4781482 SRX4781483 SRX4781484 SRX4781485 SRX4781486 SRX4781487 SRX4781488 SRX4781497 SRX4781498 SRX4781499 SRX4781500 SRX4781501 SRX4781502 SRX4781503 SRX4781504 SRX482859 SRX4872742 SRX4872743 SRX4872744 SRX4872745 SRX4882227 SRX5102886 SRX5102887 SRX5457133 SRX5457134 SRX5457145 SRX5457146 SRX5457149 SRX5457150 SRX5457152 SRX5457153 SRX5457169 SRX5457170 SRX5457174 SRX5457175 SRX5457176 SRX5457177 SRX5457187 SRX5457188 SRX5457189 SRX5457190 SRX5457192 SRX5457193 SRX5457202 SRX5457203 SRX5457209 SRX5457210 SRX5457219 SRX5457220 SRX5457237 SRX5457238 SRX5457239 SRX5457240 SRX5457243 SRX5457244 SRX5457250 SRX5457251 SRX5457252 SRX5457253 SRX5457259 SRX5457260 SRX5457261 SRX5457262 SRX5457263 SRX5457265 SRX5457266 SRX5457269 SRX5457270 SRX5457271 SRX5457272 SRX5457277 SRX5457278 SRX5457279 SRX5457280 SRX5457281 SRX5457282 SRX5457291 SRX5457292 SRX5457298 SRX5457299 SRX5457300 SRX5457301 SRX5457302 SRX5457307 SRX5457308 SRX5457312 SRX5457313 SRX5457316 SRX5457317 SRX5457324 SRX5457325 SRX5457330 SRX5457331 SRX5457336 SRX5457337 SRX5457342 SRX5457343 SRX5457352 SRX5457353 SRX5457356 SRX5457363 SRX5457364 SRX5457371 SRX5457372 SRX5457383 SRX5457384 SRX5457396 SRX5457397 SRX5457406 SRX5457407 SRX5457408 SRX5457409 SRX5457414 SRX5457415 SRX5457433 SRX5457434 SRX5457438 SRX5457439 SRX5457446 SRX5457447 SRX5457450 SRX5457451 SRX5457462 SRX5457463 SRX5457471 SRX5457472 SRX5457479 SRX5457480 SRX5457481 SRX5457482 SRX5457492 SRX5457493 SRX5457509 SRX5457510 SRX5457519 SRX5457520 SRX5457545 SRX5457546 SRX5457551 SRX5457552 SRX5457554 SRX5457555 SRX5457559 SRX5457560 SRX5457567 SRX5457568 SRX5457570 SRX5457571 SRX5457584 SRX5457585 SRX5457586 SRX5457587 SRX5457593 SRX5457594 SRX5457595 SRX5457596 SRX5457600 SRX5457601 SRX5491530 SRX5540807 SRX5540808 SRX5540810 SRX5540811 SRX5646088 SRX5646089 SRX5765527 SRX5848165 SRX5848166 SRX5848167 SRX5848171 SRX5848172 SRX5848173 SRX5848174 SRX5848175 SRX5848176 SRX5848177 SRX5848178 SRX5848179 SRX5848180 SRX5848181 SRX5848182 SRX5848183 SRX5865959 SRX5865960 SRX5865961 SRX5865962 SRX5865963 SRX5865964 SRX5865965 SRX5865966 SRX5865967 SRX5865968 SRX5865969 SRX5865970 SRX5865971 SRX5865972 SRX5865973 SRX5865974 SRX5869438 SRX5869439 SRX5974505 SRX5974506 SRX5974507 SRX5974508 SRX5974509 SRX5974510 SRX5974511 SRX5974512 SRX5974513 SRX5974514 SRX5974523 SRX5974524 SRX5974525 SRX5974526 SRX5974527 SRX5974528 SRX5974529 SRX5974530 SRX5974539 SRX5974540 SRX5974541 SRX5974542 SRX5974543 SRX5974544 SRX5974545 SRX5974546 SRX5974555 SRX5974556 SRX5974557 SRX5974558 SRX5974559 SRX5974560 SRX5974561 SRX5974562 SRX5974571 SRX5974572 SRX5974573 SRX5974574 SRX5974575 SRX5974576 SRX5974577 SRX5974578 SRX5974587 SRX5974588 SRX6686241 SRX6686243 SRX6783933 SRX6783934 SRX6783945 SRX6783946 SRX6783961 SRX6783962 SRX6783972 SRX6783973 SRX6784001 SRX6784002 SRX6784009 SRX6784010 SRX6784021 SRX6784022 SRX6784028 SRX6784029 SRX6784056 SRX6784057 SRX6784068 SRX6784069 SRX6793426 SRX6793427 SRX6828388 SRX6828389 SRX6828391 SRX6828392 SRX6828393 SRX6913072 SRX6913073 SRX6913074 SRX6913075 SRX6913076 SRX6913077 SRX6913078 SRX6913079 SRX6913080 SRX6913081 SRX6913082 SRX6913083 SRX6913085 SRX6913086 SRX6913087 SRX6913088 SRX6913089 SRX6913090 SRX6913091 SRX7398280 SRX7398281 SRX7398283 SRX7398284 SRX7478532 SRX7478534 SRX7478536 SRX7478538 SRX7494161 SRX7494162 SRX7494163 SRX7494164 SRX8299777 SRX8299778 SRX8299779 SRX8299780 SRX8299781 SRX849404 SRX895417 SRX895418 SRX895419 SRX895420 SRX895427 SRX895428 SRX895429 SRX895430 SRX895431 SRX895432 SRX895433 SRX895434 SRX9298963 SRX9298964 SRX9424509 SRX9424510 SRX9498683 SRX9498684 SRX9498685 SRX9498686 SRX9498730 SRX9498731 SRX9498732 SRX9498733 SRX9498734 SRX9498735 SRX9498736 SRX9498737 SRX9498738 SRX9498739 SRX9498740 SRX9498741 SRX9498742 SRX9498743 SRX974395 SRX9785101 SRX9785103 SRX9849092 SRX9849093 SRX9849094 SRX9849095 SRX9849096 SRX9898532 SRX9898534 SRX9898536 SRX9975166 |
| BM | Cebpa CTCF EZH2 EZH2 GATA1 GATA2 GATA3 MAX MYC POLR2A TP53 RUNX1 GATA1 TAL1 GFI1B SUPT5H CHD4 KDM6A SMAD2 SMAD2 ZBTB16 ZBTB16 SUZ12 SUZ12 RNF2 RNF2 GFI1B RUNX1 RUNX1 SPI1 SPI1 SPI1 SPI1 SPI1 TAL1 TAL1 EGR1 HDAC1 GATA2 CTCF KMT2A AFF1 NOTCH1 NETO2 MLLT3 GATA1 TAL1 GFI1B NETO2 EED SUZ12 EZH1 EZH2 TAL1 STAG1 CTCF | ENCFF484VKG ENCFF860LFC ENCFF871CHN ENCFF642TXF ENCFF155OGU ENCFF951MPF ENCFF684MET ENCFF416QKI ENCFF366KTJ ENCFF413OWL ERX181469 SRX3789540 SRX386202 SRX386203 SRX386204 SRX4385554 SRX5167211 SRX5167212 SRX5186323 SRX5186323 SRX5254847 SRX5254848 SRX5254849 SRX5254850 SRX5254851 SRX5254852 SRX5258180 SRX5567179 SRX5567180 SRX5574342 SRX5574343 SRX5574344 SRX5574345 SRX5574350 SRX658606 SRX658607 SRX6763478 SRX687554 SRX687556 SRX7626336 SRX8418557 SRX8418558 SRX751541 SRX5409709 SRX3768736 SRX386202 SRX386203 SRX386204 SRX5409709 SRX646125 SRX646126 SRX646127 SRX646129 SRX658606 SRX995497 SRX995496 |
