# Supplementary material for: Deciphering hierarchical regulatory network of cell fate via an epigenetics-informed heterogeneous graph transformer on single-cell multi-omics data
Source: Brief Bioinform. 2025 Dec 12;26(6):bbaf664. doi: 10.1093/bib/bbaf664 (PMC12875533; doi:10.1093/bib/bbaf664)
Supplement: Supplymentary_Table3_bbaf664 [file supplymentary_table3_bbaf664.docx]

# **The true CRE-CRE relationships recorded in the ENCODE dataset**

| dataset | Data source |
| --- | --- |
| K562 | ENCFF271, ENCFF693, ENCFF256, ENCFF126 |
| HCT116 | ENCFF215, ENCFF308 |
| A549 | ENCFF372, ENCFF372, ENCFF689, ENCFF689, ENCSR662, ENCSR662 |
| GM12878 | ENCFF661, ENCFF788, ENCFF041, ENCFF531, ENCFF203, ENCFF781, ENCFF378, ENCFF560, ENCFF253, ENCFF098, ENCFF083, ENCFF045 |
