# Supplementary material for: Deciphering hierarchical regulatory network of cell fate via an epigenetics-informed heterogeneous graph transformer on single-cell multi-omics data
Source: Brief Bioinform. 2025 Dec 12;26(6):bbaf664. doi: 10.1093/bib/bbaf664 (PMC12875533; doi:10.1093/bib/bbaf664)
Supplement: Supplymentary_Table4_bbaf664 [file supplymentary_table4_bbaf664.docx]

# The number of Gene and CRE

BM: TF: 355, CRE: 33152, TG: 1507

K562: TF: 715, CRE: 17182, TG: 1843

HCT116: TF:357, CRE: 14111, TG: 593

A549: TF:418, CRE: 13858, TG: 1571

A549: TF:534, CRE: 17564, TG: 1892
