# Supplementary material for: Deciphering hierarchical regulatory network of cell fate via an epigenetics-informed heterogeneous graph transformer on single-cell multi-omics data
Source: Brief Bioinform. 2025 Dec 12;26(6):bbaf664. doi: 10.1093/bib/bbaf664 (PMC12875533; doi:10.1093/bib/bbaf664)
Supplement: Supplymentary_Table5_bbaf664 [file supplymentary_table5_bbaf664.docx]

# **Proportions of prior edges vs true edges under different PCC thresholds**

| **Data** | Edge_types | | Prior network | | Pearson correlation coefficient threshold | | | | | | | | | |
| --- | --- | --- | --- | --- | --- | --- | --- | --- | --- | --- | --- | --- | --- | --- |
|  | Start | End | Prior | Truth | Prior | Truth | Prior | Truth | Prior | Truth | Prior | Truth | Prior | Truth |
| **BM** | TF | TF | 37773 |  |  |  |  |  |  |  |  |  |  |  |
|  | TF | CRE | 974684 | 44622 | >0.15 | | >0.2 | | >0.3 | | >0.4 | | >0.6 | |
|  |  |  |  |  | 236775 | 16866 | 151075 | 13538 | 62158 | 8863 | 24481 | 5381 | 1495 | 991 |
|  | CRE | CRE | 410158 |  |  |  |  |  |  |  |  |  |  |  |
|  | CRE | TG | 2618 |  |  |  |  |  |  |  |  |  |  |  |
|  | CRE | TF | 396 |  |  |  |  |  |  |  |  |  |  |  |
| **K562** | TF | TF | 103501 |  |  |  |  |  |  |  |  |  |  |  |
|  | TF | CRE | 1019951 | 327272 | >0.15 | | >0.2 | | >0.3 | | >0.4 | | >0.6 | |
|  |  |  |  |  | 238220 | 79418 | 164907 | 54938 | 75424 | 24803 | 33964 | 11033 | 5343 | 1882 |
|  | CRE | CRE | 606018 | 220486 | >0.4 | | >0.5 | | >0.6 | | >0.7 | | >0.8 | |
|  |  |  |  |  | 75526 | 33384 | 22023 | 10046 | 5467 | 2277 | 1101 | 487 | 99 | 50 |
|  | CRE | TG | 7171 |  |  |  |  |  |  |  |  |  |  |  |
|  | CRE | TF | 1910 |  |  |  |  |  |  |  |  |  |  |  |
| **HCT116** | TF | TF | 34582 |  |  |  |  |  |  |  |  |  |  |  |
|  | TF | CRE | 438870 | 20269 | >0.15 | | >0.2 | | >0.3 | | >0.4 | | >0.6 | |
|  |  |  |  |  | 167410 | 7742 | 117874 | 5773 | 57662 | 3382 | 25562 | 2104 | 3418 | 469 |
|  | CRE | CRE | 79083 | 15449 | >0.2 | | >0.3 | | >0.4 | | >0.5 | | >0.6 | |
|  |  |  |  |  | 47422 | 10479 | 29388 | 6877 | 15408 | 3920 | 7427 | 2140 | 3418 | 469 |
|  | CRE | TG | 1149 |  |  |  |  |  |  |  |  |  |  |  |
|  | CRE | TF | 171 |  |  |  |  |  |  |  |  |  |  |  |
| **A549** | TF | TF | 55013 |  |  |  |  |  |  |  |  |  |  |  |
|  | TF | CRE | 628974 | 46412 | >0.1 | | >0.15 | | >0.2 | | >0.3 | | >0.4 | |
|  |  |  |  |  | 120537 | 5618 | 46412 | 1746 | 15956 | 432 | 790 | 26 | 8 | 1 |
|  | CRE | CRE | 174522 | 17332 | >0.2 | | >0.3 | | >0.4 | | >0.5 | | >0.6 | |
|  |  |  |  |  | 7353 | 5602 | 6495 | 5018 | 6019 | 4664 | 5827 | 4528 | 5785 | 4498 |
|  | CRE | TG | 5303 |  |  |  |  |  |  |  |  |  |  |  |
|  | CRE | TF | 935 |  |  |  |  |  |  |  |  |  |  |  |
| **GM12878** | TF | TF | 59892 |  |  |  |  |  |  |  |  |  |  |  |
|  | TF | CRE | 738829 | 137570 | >0.1 | | >0.15 | | >0.2 | | >0.3 | | >0.4 | |
|  |  |  |  |  | 120537 | 30194 | 46412 | 12791 | 15956 | 4872 | 790 | 301 | 9 | 3 |
|  | CRE | CRE | 207327 | 105899 | >0.2 | | >0.3 | | >0.4 | | >0.5 | | >0.6 | |
|  |  |  |  |  | 7353 | 6967 | 6495 | 6198 | 6019 | 5758 | 5827 | 5580 | 5785 | 5544 |
|  | CRE | TG | 5813 |  |  |  |  |  |  |  |  |  |  |  |
|  | CRE | TF | 1298 |  |  |  |  |  |  |  |  |  |  |  |
