# Supplementary material for: Deciphering hierarchical regulatory network of cell fate via an epigenetics-informed heterogeneous graph transformer on single-cell multi-omics data
Source: Brief Bioinform. 2025 Dec 12;26(6):bbaf664. doi: 10.1093/bib/bbaf664 (PMC12875533; doi:10.1093/bib/bbaf664)
Supplement: Supplymentary_Table6_bbaf664 [file supplymentary_table6_bbaf664.docx]

# Statistical Characterization of co-CRE Subnetworks Computed by SMOGT, Cicero, and ArchR

| Dataset | algorithms | The number of co-CRE module |  | The number of node in co-CRE module | The number of edges in co-CRE module | Density | Dinstance(KB) | Avarege of Chromatin Accessibility | Sd of Chromatin Accessibility |
| --- | --- | --- | --- | --- | --- | --- | --- | --- | --- |
| BM | Cicero | 813 | Avarege | 50 | 889 | 0.893 | 172.485 | 0.446 | 0.368 |
|  |  |  | 25% | 20 | 218 | 0.507 | 162.781 | 0.378 | 0.350 |
|  |  |  | 75% | 70 | 1253 | 1.181 | 188.185 | 0.502 | 0.389 |
|  | ArchR | 748 | Avarege | 43 | 894 | 0.887 | 172.760 | 0.447 | 0.368 |
|  |  |  | 25% | 14 | 231 | 0.502 | 163.037 | 0.378 | 0.350 |
|  |  |  | 75% | 68 | 1260 | 1.175 | 188.565 | 0.502 | 0.389 |
|  | our | 130 | Avarege | 59 | 1259 | 0.647 | 40891.647 | 0.550 | 0.430 |
|  |  |  | 25% | 29 | 227 | 0.429 | 23369.886 | 0.466 | 0.379 |
|  |  |  | 75% | 78 | 1453 | 0.778 | 55834.899 | 0.623 | 0.493 |
| CC | Cicero | 730 | Avarege | 34 | 412 | 0.822 | 179.119 | 0.446 | 0.783 |
|  |  |  | 25% | 16 | 116 | 0.497 | 171.343 | 0.590 | 0.685 |
|  |  |  | 75% | 48 | 573 | 1.031 | 193.159 | 0.684 | 0.878 |
|  | ArchR | 724 | Avarege | 31 | 370 | 0.907 | 176.875 | 0.447 | 0.368 |
|  |  |  | 25% | 15 | 110 | 0.570 | 166.662 | 0.588 | 0.785 |
|  |  |  | 75% | 39 | 492 | 1.162 | 192.243 | 0.686 | 0.688 |
|  | our | 150 | Avarege | 99 | 4483 | 0.842 | 34643.330 | 0.710 | 0.756 |
|  |  |  | 25% | 46 | 1019 | 0.444 | 16574.434 | 0.545 | 1.026 |
|  |  |  | 75% | 143 | 5596 | 1.230 | 50642.943 | 0.805 | 0.896 |
| PBMC | Cicero | 626 | Avarege | 23 | 272 | 1.180 | 163.344 | 0.446 | 0.783 |
|  |  |  | 25% | 13 | 94 | 0.797 | 148.520 | 0.584 | 0.534 |
|  |  |  | 75% | 28 | 344 | 1.556 | 184.023 | 0.464 | 0.497 |
|  | ArchR | 623 | Avarege | 23 | 273 | 1.166 | 163.393 | 0.690 | 0.574 |
|  |  |  | 25% | 30 | 96 | 0.776 | 148.589 | 0.464 | 0.497 |
|  |  |  | 75% | 13 | 352 | 1.536 | 183.874 | 0.691 | 0.575 |
|  | our | 132 | Avarege | 86 | 2649 | 0.325 | 38353.666 | 0.669 | 0.571 |
|  |  |  | 25% | 37 | 405 | 0.633 | 20869.327 | 0.486 | 0.529 |
|  |  |  | 75% | 129 | 3917 | 0.847 | 52183.905 | 0.766 | 0.642 |
| A549 | Cicero | 408 | Avarege | 19 | 348 | 1.545 | 141.023 | 0.105 | 0.197 |
|  |  |  | 25% | 11 | 87 | 1.211 | 114.460 | 0.071 | 0.164 |
|  |  |  | 75% | 23 | 356 | 1.944 | 172.367 | 0.125 | 0.221 |
|  | ArchR | 473 | Avarege | 22 | 389 | 1.380 | 144.078 | 0.097 | 0.184 |
|  |  |  | 25% | 11 | 90 | 0.986 | 120.730 | 0.067 | 0.152 |
|  |  |  | 75% | 28 | 395 | 1.857 | 174.494 | 0.114 | 0.206 |
|  | Our | 198 | Avarege | 34 | 801 | 1.112 | 30924.856 | 0.103 | 0.192 |
|  |  |  | 25% | 17 | 161 | 0.736 | 9634.287 | 0.059 | 0.138 |
|  |  |  | 75% | 47 | 1117 | 1.469 | 47740.877 | 0.090 | 0.205 |
| GM12878 | Cicero | 408 | Avarege | 19 | 348 | 1.545 | 141.023 | 0.105 | 0.197 |
|  |  |  | 25% | 11 | 87 | 1.211 | 114.460 | 0.071 | 0.164 |
|  |  |  | 75% | 23 | 356 | 1.944 | 172.367 | 0.125 | 0.221 |
|  | ArchR | 472 | Avarege | 22 | 389 | 1.380 | 144.078 | 0.097 | 0.184 |
|  |  |  | 25% | 11 | 90 | 0.986 | 120.730 | 0.067 | 0.152 |
|  |  |  | 75% | 28 | 395 | 1.857 | 174.494 | 0.067 | 0.206 |
|  | our | 68 | Avarege | 21 | 176 | 0.862 | 41389.216 | 0.137 | 0.231 |
|  |  |  | 25% | 13 | 64 | 0.433 | 18222.028 | 0.074 | 0.159 |
|  |  |  | 75% | 25 | 266 | 1.238 | 59336.347 | 0.202 | 0.306 |
| HCT116 | Cicero | 468 | Avarege | 26 | 559 | 1.269 | 160.190 | 0.260 | 0.229 |
|  |  |  | 25% | 12 | 101 | 0.904 | 145.669 | 0.215 | 0.195 |
|  |  |  | 75% | 29 | 410 | 1.679 | 181.374 | 0.291 | 0.254 |
|  | ArchR | 463 | Avarege | 26 | 567 | 1.251 | 160.347 | 0.260 | 0.229 |
|  |  |  | 25% | 12 | 105 | 0.878 | 145.811 | 0.215 | 0.195 |
|  |  |  | 75% | 30 | 423 | 1.649 | 181.397 | 0.291 | 0.254 |
|  | our | 99 | Avarege | 100 | 2931 | 0.579 | 26366.907 | 0.323 | 0.571 |
|  |  |  | 25% | 46 | 567 | 0.331 | 3622.332 | 0.259 | 0.280 |
|  |  |  | 75% | 144 | 4395 | 0.745 | 41635.270 | 0.365 | 0.228 |
| K562 | Cicero | 498 | Avarege | 26 | 759 | 1.167 | 163.344 | 0.265 | 0.241 |
|  |  |  | 25% | 12 | 90 | 0.777 | 148.520 | 0.217 | 0.204 |
|  |  |  | 75% | 29 | 358 | 1.556 | 185.872 | 0.464 | 0.271 |
|  | ArchR | 498 | Avarege | 26 | 760 | 1.154 | 162.498 | 0.264 | 0.241 |
|  |  |  | 25% | 12 | 90 | 0.769 | 146.267 | 0.217 | 0.204 |
|  |  |  | 75% | 29 | 358 | 1.512 | 185.941 | 0.299 | 0.217 |
|  | our | 112 | Avarege | 64 | 2842 | 1.099 | 41758.042 | 0.256 | 0.233 |
|  |  |  | 25% | 30 | 485 | 0.826 | 20522.758 | 0.139 | 0.138 |
|  |  |  | 75% | 87 | 3593 | 1.368 | 62947.764 | 0.358 | 0.328 |
